# Global, regional, and national burden of neuroblastoma and peripheral nervous system tumours in individuals aged over 60 from 1990 to 2021: a trend analysis of global burden of disease study

**DOI:** 10.1186/s41043-025-00810-9

**Published:** 2025-03-17

**Authors:** Zihan Ding, Yun Chen, Genbo Huang, Rongbo Liao, Houting Zhang, Shifa Zhou, XuKai Liu

**Affiliations:** 1 Department of Neurosurgery, Zhuzhou Hospital Affiliated to Xiangya School of Medicine, Central South University, Zhuzhou, Hunan Province 412000 People’s Republic of China; 2 Department of Trauma and Acute Care Surgery, the 1st Affiliated Hospital, Jiangxi Medical College, Nanchang University, 17 Yongwai Street, Nanchang, 330006 Jiangxi China; 3 Department of Neurosurgery, the 1st Affiliated Hospital, Jiangxi Medical College, Nanchang University, 17 Yongwai Street, Nanchang, 330006 Jiangxi China

**Keywords:** Neuroblastoma and peripheral nervous system tumours, Disease burden, The elderly, Sociodemographic level, GBD 2021

## Abstract

**Purpose:**

Elderly individuals diagnosed with neuroblastoma and peripheral nervous system tumours often have a poor prognosis. However, there is currently a lack of comprehensive analysis on these conditions in older adults. This study aims to determine the global epidemiological trends of neuroblastoma and peripheral nervous system tumours (in individuals aged 60 and above).

**Methods:**

We obtained cross-sectional data from the 2021 Global Burden of Disease, Injuries, and Risk Factors Study (GBD) (https://vizhub.healthdata.org/gbd-results/). We assessed the burden of neuroblastoma and peripheral nervous system tumours in the elderly from 1990 to 2021 using indicators such as prevalence and incidence. These indicators were classified by global, national, and regional levels, further stratified by Socio-Demographic Index (SDI), age, and gender. The results are organized by SDI, age, and gender categories.

**Results:**

From 1990 to 2021, the global age-standardised prevalence and incidence rates of neuroblastoma and peripheral nervous system tumours among the elderly increased from 0.06 (95% UI 0.05, 0.08) and 0.12 (95% UI 0.09, 0.15) per 100,000 to 0.11 (95% UI 0.09, 0.13) and 0.22 (95% UI 0.17, 0.26) per 100,000, respectively. Age-standardised mortality and DALY rates also rose. Central Europe had the highest age-standardised prevalence and incidence rates in 2021, while Eastern Europe had the highest DALY rate. East Asia reported the highest number of total cases and experienced the fastest growth, with significant increases in prevalence, incidence, mortality, and DALY rates. Gender disparities were evident, with elderly men showing higher rates than women, and greater EAPC values indicating a higher increase in disease burden over time. The highest age-specific rates were found in the 90–94 age group, while the 70–74 age group had the highest DALY burden.

**Conclusion:**

The continuous rise in the incidence of neuroblastoma and peripheral nervous system tumours among the elderly highlights a pressing the necessity for focused public health measures and improved treatment approaches. Addressing the regional, gender, and age-related disparities requires a comprehensive approach that integrates medical advancements, social support, and public health policies. Future research should explore potential risk factors and innovative therapies to mitigate this growing global health challenge.

**Supplementary Information:**

The online version contains supplementary material available at 10.1186/s41043-025-00810-9.

## Introduction

Although neuroblastoma and peripheral nervous system tumours are relatively rare, their impact on the global cancer burden cannot be ignored, particularly among children [1]. However, the influence of these tumours on the elderly population has often been overlooked. Neuroblastoma is an embryonal tumour of the sympathetic nervous system that typically occurs in early childhood, while peripheral nervous system tumours encompass a diverse range of conditions, including schwannomas, neurofibromas, and other malignancies that can affect individuals across various age groups [2, 3]. In the context of elderly individuals, these tumours are rarely studied, and the available data on their epidemiology in this age group is limited [4].

As global ageing accelerates, the healthcare demands of older populations are increasing, making it critical to assess the burden of rare diseases such as neuroblastoma and peripheral nervous system tumours in this demographic [5, 6]. The elderly population faces unique challenges, including a higher prevalence of comorbidities, a weakened immune system, and the need for specialised care [7, 8]. These factors can complicate the diagnosis, treatment, and prognosis of neuroblastoma and peripheral nervous system tumours in older adults, which warrants closer attention. Although these diseases are rare in this age group, their occurrence in elderly individuals requires focused investigation due to the potential for more severe outcomes and the specific healthcare challenges they present. Additionally, gender differences in malignancies are observed among senior populations, and this may influence the presentation and outcomes of neuroblastoma and peripheral nervous system tumours in older adults [9, 10]. These differences, though less pronounced, can still provide important insights into the disease burden and healthcare needs of elderly men and women.

Despite ongoing demographic changes, studies focusing on the burden of neuroblastoma and peripheral nervous system tumours in those aged 60 and above are relatively limited. Existing research has largely concentrated on paediatric cases, given the higher incidence of these tumours in children [11, 12]. However, the incidence of neuroblastoma and peripheral nervous system tumours in the elderly is not well documented, and the impact of these conditions on older individuals has been understudied. This represents a critical research gap. Understanding how these diseases affect elderly populations is crucial for effective healthcare planning, particularly in resource-limited settings where specialized care may be less accessible. Addressing this gap can lead to better-targeted healthcare policies, improved resource allocation, and better health outcomes for elderly individuals living with these complex diseases.

The Global Burden of Disease (GBD) study offers a comprehensive dataset that provides valuable insights into the epidemiological trends of various cancers and other diseases across different age groups and regions. However, specific data on neuroblastoma and peripheral nervous system tumours in the elderly has yet to be thoroughly investigated. This study seeks to address this gap by offering a global, regional, and national examination of the epidemiological trends of neuroblastoma and peripheral nervous system tumours in individuals aged 60 and above from 1990 to 2021. By using data from the 2021 GBD study, we will examine temporal trends, regional variations, and the epidemiological characteristics of these tumours in older adults. This will allow us to identify regions with the highest disease burden and to provide evidence-based recommendations for healthcare strategies, resource allocation, and future research directions. Ultimately, the results of this research will provide insights into improving healthcare services and health outcomes for the ageing global population.

## Methods

### Study population and data collection

This study used data sourced from the GBD 2021 dataset, which offers detailed insights into the global and regional impacts of 371 diseases and injuries, in addition to 88 risk factors, across 204 nations and territories spanning from 1990 to 2021. According to the International Classification of Diseases, 10th Revision (ICD-10), neuroblastoma and peripheral nervous system tumours are defined as malignant neoplasms of the adrenal gland, unspecified (C74.9), malignant neoplasms of peripheral nerves and autonomic nervous systems (C47), and benign neoplasms of peripheral and autonomic nervous systems (C36.1). The elderly population was defined as individuals aged 60 and above, in accordance with the World Health Organization (WHO) [13]. This study specifically focused on patients aged 60 and over with neuroblastoma and peripheral nervous system tumours and further segmented them into seven age ranges (60–64, 65–69, 70–74, 75–79, 80–84, 85–89, and 90–94) to more accurately depict the burden of these tumours within this population. This segmentation helps to capture more specific age-related trends, given the heterogeneity in health and disease presentation among older adults. The age cutoff of 60+ was chosen based on WHO’s classification and demographic studies indicating a higher incidence of age-related diseases starting at this age. We accessed and downloaded data on prevalence, incidence, mortality, and DALYs related to neuroblastoma and peripheral nervous system tumours among the elderly from the Global Health Data Exchange (GHDx) platform (http://ghdx.healthdata.org/gbd-results-tool). Data were available at global, regional (21 regions), and national (204 countries) levels. We also obtained Socio-Demographic Index (SDI) data to evaluate the impact of socioeconomic factors on the disease burden. The data used in this study are publicly available; therefore, the Ethics Committee of the First Affiliated Hospital of Nanchang University determined that ethical approval was not required. This study adhered to the guidelines for accurate and transparent reporting of cross-sectional studies as outlined in health estimation reporting standards [14].

### Statistical analysis

R (version 4.3.2) was employed for the statistical analysis, employing the ‘ggplot2’ and ‘sf’ packages for data visualisation, and the ‘broom’ and ‘dplyr’ packages for regression output processing. The 95% Uncertainty Intervals (UIs) were calculated using Monte Carlo simulations. This method was chosen due to its robustness in estimating uncertainty in complex models by repeatedly drawing random samples from the data. These simulations allow for a more comprehensive understanding of the variability in disease burden estimates.

The EAPC was calculated using linear regression models. Both univariate and multivariate regression models were used. The univariate model analyzed each condition independently, while the multivariate model included adjustments for potential confounders such as geographic region, SDI, and age group. This approach ensures that the results reflect the true trends in disease burden, accounting for external factors that could influence the data. The multivariate model was particularly important for controlling for the impact of socioeconomic and demographic factors, which may vary across regions and populations.

Given the extensive data and potential for multiple comparisons, Bonferroni correction was used to adjust for multiple testing. This method is particularly useful when conducting multiple comparisons across different regions or subgroups, reducing the risk of Type I errors and ensuring the statistical rigor of the findings.

### Systematic bias adjustment and regression analyses

To assess systematic biases and ensure the validity of our findings, we implemented regression analyses with adjustments for multiple confounding variables. Specifically, the analysis was conducted for each disease and injury type, with multivariate regression models applied to adjust for factors such as geographic region, SDI, and age group. The process for adjusting for systematic bias was as follows:

Geographic adjustment: Geographic regions were included as independent variables in the regression models to control for regional differences in disease burden. This adjustment allowed us to account for disparities in disease incidence that could arise from location-specific factors. SDI adjustment: The Socio-Demographic Index (SDI), which measures the socioeconomic development of a region, was included as a covariate to account for variations in disease burden among regions with different levels of economic and social conditions. Age group adjustment: Age-specific trends were considered by stratifying data into various age groups (e.g., 60–64, 65–69, 70–74) to account for the potential impact of age on disease burden, especially given that older populations may exhibit different disease trends compared to younger groups. For each disease and injury, the following procedure was followed to ensure the estimates were as accurate and reliable as possible: Initial estimation: We first derived the raw estimates of disease burden (prevalence, incidence, mortality, and DALYs) from the GBD dataset at global, regional, and national levels. Systematic bias adjustment: Using multivariate regression models, we adjusted the raw estimates for factors such as geographic region, SDI, and age group. This adjustment process mitigated the effects of systematic biases and confounding variables that might skew the results. EAPC calculation: The EAPC was calculated by applying linear regression models to the adjusted estimates, providing a measure of trend direction and rate of change in disease burden over time. Uncertainty assessment: We incorporated Monte Carlo simulations to calculate 95% Uncertainty Intervals (UIs) for each estimate, reflecting the inherent uncertainty in the data and the modeling process. These steps ensured that the final estimates and trends were adjusted for systematic biases, accurately reflecting the true patterns of disease burden across different regions and populations.

### Prevalence and incidence calculation

In the statistical analysis, prevalence and incidence were calculated based on the detailed information in the GBD dataset. For each disease and injury, we first calculated the number of prevalent and incident cases per 1000 people, stratified by age group, sex, and geographic region. These calculations rely on the total case counts and population base provided in the dataset, using the following formulas:

$$\begin{aligned} & {\text{Prevalence }} = \, \left( {{\text{Total cases of a specific disease or injury }}/{\text{ Total population}}} \right) \, *{ 1}000 \\ & {\text{Incidence }} = \, \left( {{\text{New cases of a specific disease or injury }}/{\text{ Total population}}} \right) \, *{ 1}000 \\ \end{aligned}$$

These data were aggregated and standardized at the global, regional, and national levels to ensure comparability across different regions.

### Exclusion criteria

No exclusion criteria were used in selecting the data, as we employed the complete and publicly available GBD dataset for all regions and conditions. This ensures that the analysis reflects the most comprehensive and inclusive dataset available.

### Software and reproducibility

Finally, the reason R was chosen for statistical analysis lies in its flexibility, reproducibility, and the extensive set of packages available for complex statistical modeling and visualisation. R ensures transparency in data processing and result reporting, and the codes used for statistical analyses will be made available for full reproducibility.

## Results

### Global trends

**Fig. 1:**
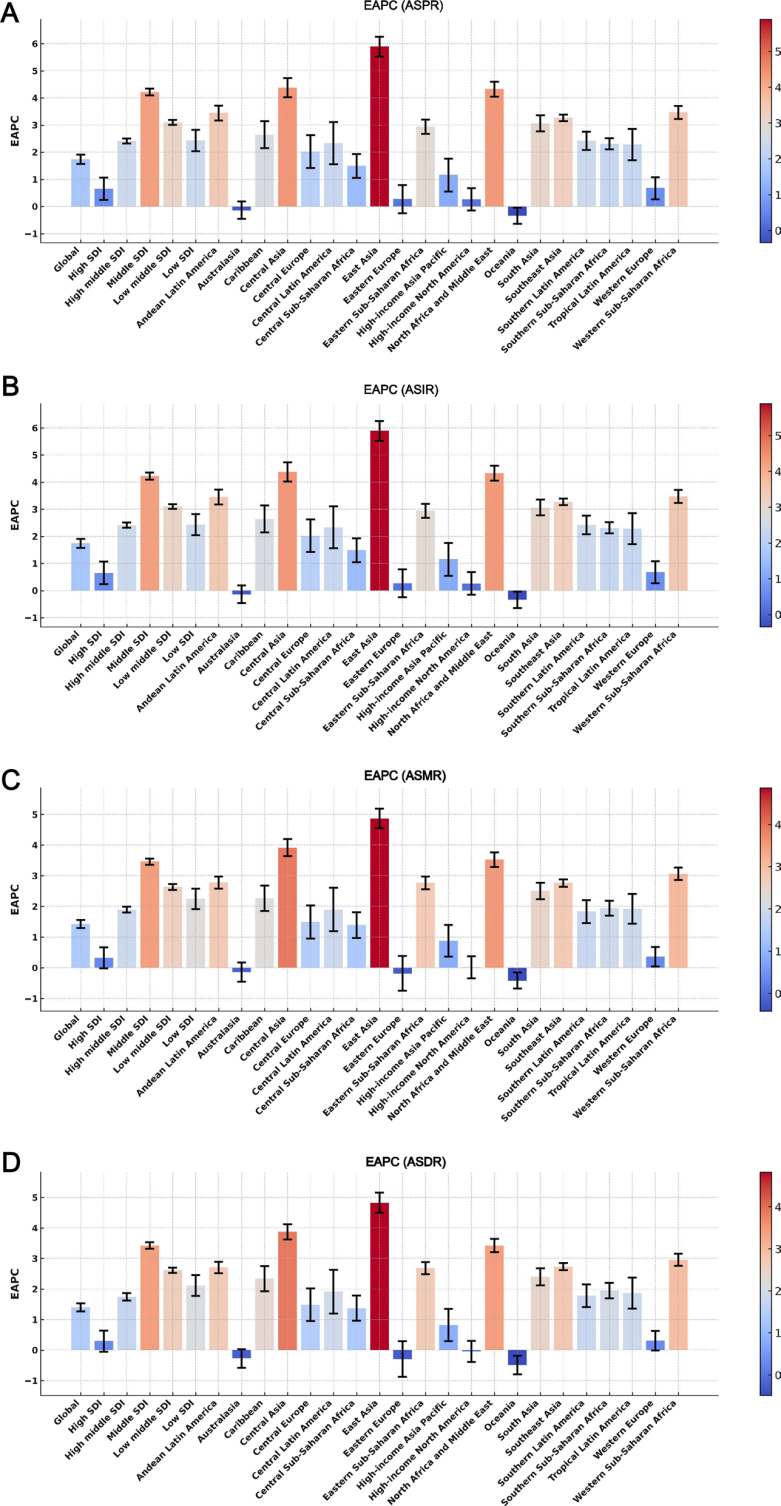
EAPCs in age-standardised prevalence (**A**), incidence (**B**), mortality (**C**), and DALYs (**D**) for neuroblastoma and peripheral nervous system tumours, stratified by GBD regions and SDI quintiles, from 1990 to 2021. *EAPC* Estimated Average Percentage Changes, *DALYs* Disability-adjusted Life-Years, *SDI* Socio-demographic Index

Globally, the number of elderly individuals (aged 60 and above) diagnosed with neuroblastoma and peripheral nervous system tumours increased from 281.36 (95% UI 221.73, 352.95) per 100,000 in 1990 to 1166.65 (95% UI 918.18, 1413.19) per 100,000 in 2021, representing an increase of 314.65%. Simultaneously, the age-standardised prevalence rate increased from 0.06 per 100,000 (95% UI 0.05, 0.08) in 1990 to 0.11 per 100,000 (95% UI 0.09, 0.13) in 2021, with an EAPC of 1.74 (95% CI 1.57, 1.91) (Fig. 1A, Table 1). Regarding incidence, the number of new cases rose from 562.72 (95% UI 443.46, 705.91) per 100,000 in 1990 to 2333.31 (95% UI 1836.36, 2826.39) per 100,000 in 2021, also marking a 314.65% increase. The age-standardised incidence rate showed a similar EAPC of 1.74 (95% CI 1.57, 1.91), increasing from 0.12 per 100,000 (95% UI 0.09, 0.15) in 1990 to 0.22 per 100,000 (95% UI 0.17, 0.26) in 2021 (Fig. 1B, Supplementary Table 1). Regarding mortality, the death count in 2021 was 1593.92 (95% UI 1329.94, 1781.63) per 100,000, a significant rise from 424.42 (95% UI 361.82, 491.51) per 100,000 in 1990, representing an increase of 275.55%. The age-standardised mortality rate similarly showed a notable increase, rising from 0.09 per 100,000 (95% UI 0.08, 0.11) in 1990 to 0.15 per 100,000 (95% UI 0.12, 0.17) in 2021, accompanied by an EAPC of 1.42 (95% CI 1.29, 1.56) (Fig. 1C, Supplementary Table 2). Regarding DALYs, the total number of DALYs in 2021 resulting from neuroblastoma and peripheral nervous system tumours in the elderly was 32,050.19 (95% UI 26,840.23, 35,736.83), an increase of 262.53% compared to 8,840.78 (95% UI 7,492.67, 10,292.56) in 1990. The age-standardised DALY rate also rose from 1.82 per 100,000 (95% UI 1.55, 2.12) in 1990 to 2.95 per 100,000 (95% UI 2.47, 3.29) in 2021, with an EAPC of 1.40 (95% CI 1.27, 1.53) (Fig. 1D, Supplementary Table 3).

**Table 1:** The age-standardized prevalence rate, number of cases, and EAPC of neuroblastoma and peripheral nervous system tumours among elderly individuals aged 60 and above globally and regionally from 1990 to 2021

	Prevalence (95% UI)				
	Cases in 1990 (million)	Age-standardised rate in 1990 (per 100,000)	Cases in 2021(million)	Age-standardised rate in 2021 (per 100,000)	EAPC (95% CI)
Global	281.36 (221.73, 352.95)	0.06 (0.05, 0.08)	1166.65 (918.18, 1413.19)	0.11 (0.09, 0.13)	1.74 (1.57, 1.91)
*Sex*					
Male	143.92 (112.09, 180.82)	0.07 (0.05, 0.09)	668.19 (529.78, 813.17)	0.14 (0.11, 0.17)	2.01 (1.85, 2.18)
Female	137.44 (106.34, 176.47)	0.05 (0.04, 0.07)	498.47 (355.55, 622.22)	0.09 (0.06, 0.11)	1.39 (1.21, 1.58)
*SDI quintile*					
High	147.70 (121.50, 176.18)	0.10 (0.08, 0.12)	381.67 (312.41, 449.88)	0.14 (0.11, 0.16)	0.65 (0.24, 1.07)
High middle	90.56 (64.54, 120.79)	0.07 (0.05, 0.10)	400.74 (299.04, 498.47)	0.16 (0.12, 0.20)	2.41 (2.32, 2.51)
Middle	31.86 (22.08, 45.70)	0.03 (0.02, 0.04)	315.15 (232.90, 399.48)	0.10 (0.07, 0.12)	4.22 (4.09, 4.35)
Low middle	8.97 (5.66, 14.51)	0.01 (0.01, 0.02)	58.99 (43.89, 79.21)	0.04 (0.03, 0.05)	3.10 (3.01, 3.19)
Low	1.89 (0.88, 3.89)	0.01 (0.00, 0.02)	8.92 (5.21, 14.77)	0.02 (0.01, 0.03)	2.43 (2.04, 2.82)
*GBD region*					
Andean Latin America	1.01 (0.68, 1.51)	0.04 (0.03, 0.06)	8.25 (5.54, 11.87)	0.12 (0.08, 0.17)	3.45 (3.17, 3.72)
Australasia	4.19 (3.22, 5.28)	0.14 (0.10, 0.17)	10.23 (7.51, 13.66)	0.14 (0.11, 0.19)	− 0.14 (− 0.46, 0.19)
Caribbean	1.40 (1.02, 1.91)	0.04 (0.03, 0.06)	5.65 (4.22, 7.44)	0.08 (0.06, 0.11)	2.64 (2.15, 3.14)
Central Asia	2.34 (1.26, 3.68)	0.04 (0.02, 0.07)	12.13 (8.87, 16.12)	0.13 (0.09, 0.17)	4.37 (4.02, 4.73)
Central Europe	14.97 (11.31, 19.30)	0.08 (0.06, 0.10)	48.46 (38.57, 59.69)	0.16 (0.13, 0.20)	2.02 (1.42, 2.63)
Central Latin America	3.60 (2.81, 4.79)	0.04 (0.03, 0.05)	28.76 (22.08, 36.33)	0.09 (0.07, 0.12)	2.33 (1.56, 3.11)
Central Sub-Saharan Africa	0.29 (0.13, 0.62)	0.01 (0.01, 0.03)	1.10 (0.53, 2.18)	0.02 (0.01, 0.04)	1.49 (1.05, 1.93)
East Asia	29.88 (19.09, 46.17)	0.03 (0.02, 0.05)	399.83 (262.84, 522.80)	0.15 (0.10, 0.19)	5.89 (5.52, 6.25)
Eastern Europe	37.30 (22.86, 53.16)	0.10 (0.06, 0.14)	73.96 (58.63, 91.66)	0.15 (0.12, 0.19)	0.27 (− 0.25, 0.79)
Eastern Sub-Saharan Africa	0.71 (0.33, 1.51)	0.01 (0.00, 0.02)	3.77 (2.09, 6.57)	0.02 (0.01, 0.04)	2.94 (2.68, 3.20)
High-income Asia Pacific	15.40 (12.61, 18.64)	0.06 (0.05, 0.07)	64.87 (52.28, 77.75)	0.11 (0.09, 0.13)	1.16 (0.55, 1.76)
High-income North America	50.39 (41.26, 60.52)	0.11 (0.09, 0.13)	111.52 (91.86, 131.50)	0.13 (0.10, 0.15)	0.26 (− 0.15, 0.68)
North Africa and Middle East	3.91 (2.08, 6.85)	0.02 (0.01, 0.04)	36.41 (25.15, 51.74)	0.07 (0.05, 0.10)	4.33 (4.05, 4.60)
Oceania	0.04 (0.02, 0.09)	0.01 (0.01, 0.03)	0.11 (0.06, 0.23)	0.01 (0.01, 0.03)	− 0.34 (− 0.64, − 0.04)
South Asia	6.98 (4.14, 11.35)	0.01 (0.01, 0.02)	54.29 (40.01, 74.19)	0.03 (0.02, 0.04)	3.06 (2.77, 3.36)
Southeast Asia	7.87 (5.07, 11.77)	0.03 (0.02, 0.04)	60.95 (44.50, 82.75)	0.08 (0.06, 0.11)	3.27 (3.15, 3.39)
Southern Latin America	4.10 (2.79, 5.87)	0.07 (0.05, 0.10)	14.15 (10.01, 19.19)	0.13 (0.09, 0.17)	2.42 (2.08, 2.76)
Southern Sub-Saharan Africa	1.39 (0.72, 2.16)	0.05 (0.02, 0.07)	6.20 (3.96, 8.45)	0.10 (0.06, 0.13)	2.31 (2.11, 2.52)
Tropical Latin America	4.83 (3.71, 6.45)	0.05 (0.04, 0.06)	33.18 (25.10, 42.79)	0.10 (0.08, 0.13)	2.28 (1.71, 2.86)
Western Europe	89.32 (73.49, 106.66)	0.12 (0.10, 0.14)	185.02 (148.23, 222.96)	0.15 (0.12, 0.18)	0.68 (0.27, 1.08)
Western Sub-Saharan Africa	1.44 (0.75, 2.59)	0.02 (0.01, 0.03)	7.83 (4.88, 11.31)	0.04 (0.03, 0.06)	3.47 (3.23, 3.71)

*EAPC* Estimated Annual Percentage Change

### Global trends by gender

**Fig. 2:**
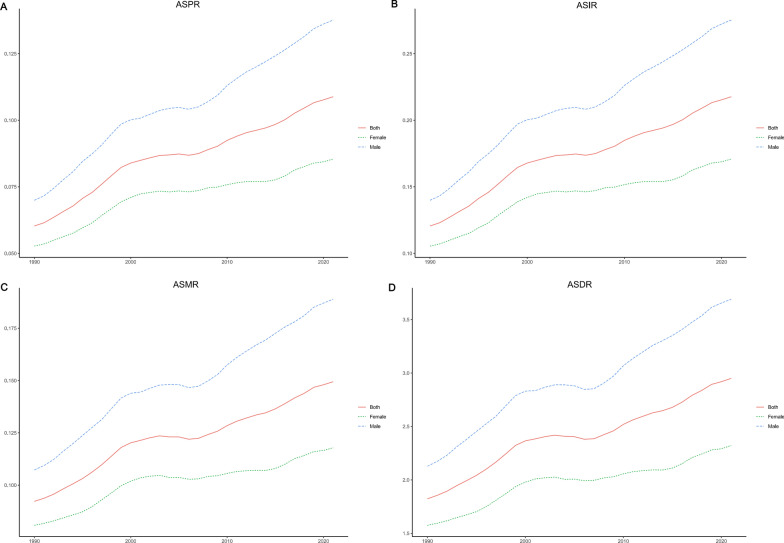
Trends in age-standardised prevalence (**A**), incidence (**B**), mortality (**C**), and DALYs rates (**D**) of neuroblastoma and peripheral nervous system tumours among individuals aged 60 years and older, classified by gender, from 1990 to 2021. *ASPR* Age-standardized Prevalence Rate, *ASIR* Age-standardized Incidence Rate, *ASMR* Age-standardized Mortality Rate, *ASDR* Age-standardized, *DALYs* Disability-adjusted Life-Years

Between 1990 and 2021, the age-standardised prevalence, incidence, mortality, and DALY rates for neuroblastoma and peripheral nervous system tumours among the elderly population showed a marked upward trend for both males and females globally, an even more noticeable rise was seen in males (Fig. 2A–D). Notably, the trends in age-standardised prevalence and incidence were consistent, with males having an EAPC of 2.01 (95% CI 1.85, 2.18) compared to 1.39 (95% CI 1.21, 1.58) for females (Fig. 2A, B, Table 1, Supplementary Table 1). For age-standardised mortality rates, the EAPC for males was 1.69 (1.56, 1.82), while for females, it was 1.08 (95% CI 0.93, 1.24). Similarly, in terms of age-standardised DALY rates, males had an EAPC of 1.62 (95% CI 1.49, 1.75), compared to 1.11 (95% CI 0.97, 1.25) for females (Fig. 2C, D, Supplementary Table 2 and Table 3). Form 1990 to 2021, the disease burden of neuroblastoma and peripheral nervous system tumours among elderly males has consistently been greater than that observed in females.

### Global trends by age groups

**Fig. 3:**
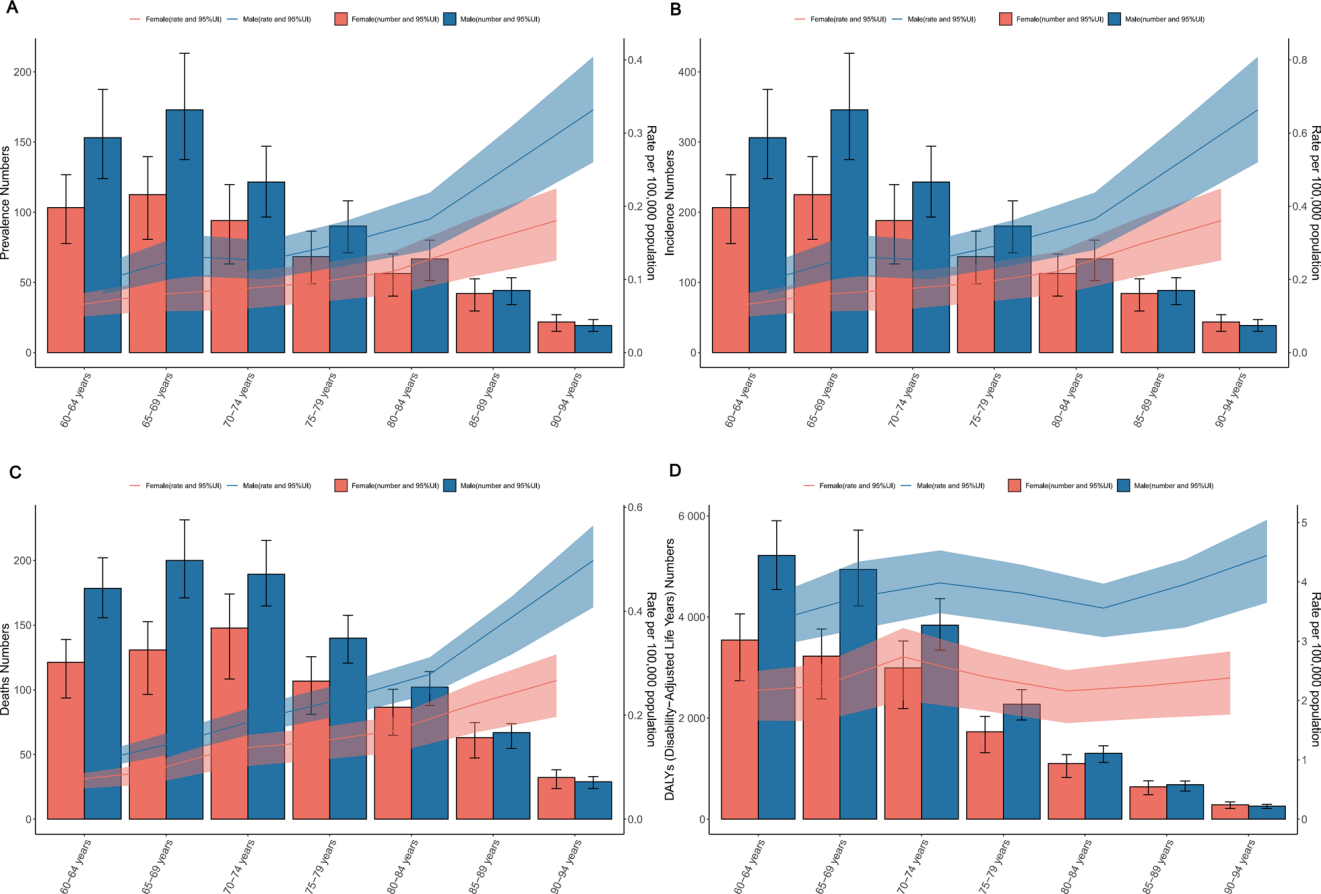
Age distribution of the total number of cases and age-standardised prevalence (**A**), incidence (**B**), mortality (**C**), and DALYs (**D**) due to neuroblastoma and peripheral nervous system tumours among individuals aged 60 years and older, classified by sex, globally in 2021, with error bars indicating the 95% uncertainty range for case numbers, while the shading represents the 95% uncertainty range for the rates. DALYs, Disability-adjusted Life-Years

Globally, in 2021, the age-specific prevalence, incidence, and mortality rates of neuroblastoma and peripheral nervous system tumours among the elderly exhibited a gradual increase with advancing age, peaking within the 90–94 age group. The rates for this age group reached 0.23 per 100,000 (95% UI 0.28, 0.17) for prevalence, 0.46 per 100,000 (95% UI 0.56, 0.34) for incidence, and 3.05 per 100,000 (95% UI 3.49, 2.43) for mortality (Fig. 3A–C, Table 2). The number of cases, including prevalence, incidence, and mortality, peaked predominantly within the 65–69 age group, with figures of 41.08 (95% UI 50.04, 30.84), 82.16 (95% UI 100.08, 61.68), and 61.17 (95% UI 70.02, 48.56), respectively. Following this peak, the numbers showed a consistent decline as age increased (Fig. 3A–C). In contrast, the age-specific DALY rate demonstrated a relatively stable trend, with its highest point observed in the 70–74 age group at 3.32 per 100,000 (95% UI 2.74, 3.71). The peak number of DALYs was concentrated in the 60–64 age group, reaching 8766.73 (95% UI 7257.25, 9675.18), and subsequently declined gradually with age (Fig. 3D, Table 2).

**Table 2:** Age-specific prevalence, incidence, mortality rates, and DALYs, as well as the corresponding case numbers, for elderly individuals across different age groups

		Age groups						
		60–64	65–69	70–74	75–79	80–84	85–89	90–94
*Rate*								
Prevalence (95% UI)	Male	0.1 (0.08, 0.12)	0.13 (0.1, 0.16)	0.13 (0.1, 0.15)	0.15 (0.12, 0.18)	0.18 (0.14, 0.22)	0.26 (0.2, 0.31)	0.33 (0.26, 0.4)
	Female	0.06 (0.05, 0.08)	0.08 (0.06, 0.1)	0.09 (0.06, 0.11)	0.09 (0.07, 0.12)	0.11 (0.08, 0.14)	0.15 (0.1, 0.18)	0.18 (0.13, 0.22)
	Both	0.08 (0.06, 0.1)	0.1 (0.08, 0.13)	0.1 (0.08, 0.13)	0.12 (0.09, 0.14)	0.14 (0.11, 0.17)	0.19 (0.14, 0.23)	0.23 (0.17, 0.28)
Incidence (95% UI)	Male	0.2 (0.16, 0.24)	0.26 (0.21, 0.32)	0.25 (0.2, 0.31)	0.3 (0.24, 0.36)	0.36 (0.28, 0.44)	0.51 (0.4, 0.62)	0.66 (0.52, 0.81)
	Female	0.13 (0.09, 0.15)	0.16 (0.11, 0.19)	0.17 (0.12, 0.22)	0.19 (0.14, 0.24)	0.22 (0.16, 0.28)	0.3 (0.21, 0.37)	0.36 (0.25, 0.45)
	Both	0.16 (0.13, 0.19)	0.21 (0.17, 0.25)	0.21 (0.16, 0.25)	0.24 (0.19, 0.29)	0.28 (0.22, 0.34)	0.38 (0.29, 0.46)	0.46 (0.34, 0.56)
Deaths (95% UI)	Male	0.11 (0.1, 0.13)	0.15 (0.13, 0.18)	0.2 (0.17, 0.22)	0.23 (0.2, 0.26)	0.28 (0.24, 0.31)	0.39 (0.32, 0.43)	0.5 (0.41, 0.56)
	Female	0.07 (0.06, 0.08)	0.09 (0.07, 0.11)	0.13 (0.1, 0.16)	0.15 (0.11, 0.17)	0.17 (0.13, 0.2)	0.22 (0.17, 0.26)	0.27 (0.2, 0.32)
	Both	0.09 (0.08, 0.1)	0.12 (0.1, 0.13)	0.16 (0.14, 0.18)	0.19 (0.16, 0.21)	0.22 (0.18, 0.24)	0.28 (0.23, 0.32)	0.34 (0.27, 0.39)
DALYs (95% UI)	Male	3.36 (2.92, 3.8)	3.75 (3.2, 4.34)	3.98 (3.47, 4.53)	3.81 (3.28, 4.29)	3.56 (3.07, 3.97)	3.96 (3.23, 4.38)	4.45 (3.65, 5.05)
	Female	2.16 (1.66, 2.47)	2.24 (1.65, 2.61)	2.73 (2, 3.22)	2.4 (1.83, 2.82)	2.16 (1.62, 2.51)	2.25 (1.7, 2.67)	2.38 (1.76, 2.83)
	Both	2.74 (2.35, 3.02)	2.96 (2.46, 3.33)	3.32 (2.74, 3.71)	3.04 (2.54, 3.37)	2.74 (2.27, 3.06)	2.9 (2.39, 3.29)	3.05 (2.43, 3.49)
*Number*								
Prevalence (95% UI)	Male	153.07 (123.92, 187.65)	172.91 (137.53, 213.27)	121.57 (96.49, 147.07)	90.23 (71.17, 108.19)	66.78 (51.32, 80.09)	44.3 (34.2, 53.33)	19.32 (15.15, 23.57)
	Female	103.26 (77.57, 126.69)	112.59 (80.67, 139.57)	94 (63.1, 119.72)	68.44 (49.07, 86.51)	56.36 (40.26, 70.21)	42.05 (29.69, 52.47)	21.76 (15.19, 27.04)
	Both	256.33 (208.01, 308.18)	285.51 (228.32, 351.62)	215.57 (165.18, 261.34)	158.67 (123.63, 189.5)	123.14 (95.91, 147.47)	86.35 (66.29, 105.03)	41.08 (30.84, 50.04)
Incidence (95% UI)	Male	306.15 (247.85, 375.31)	345.83 (275.05, 426.54)	243.14 (192.99, 294.14)	180.46 (142.35, 216.39)	133.56 (102.64, 160.18)	88.6 (68.4, 106.65)	38.65 (30.29, 47.14)
	Female	206.52 (155.14, 253.38)	225.19 (161.34, 279.15)	188 (126.19, 239.45)	136.89 (98.13, 173.03)	112.73 (80.52, 140.42)	84.1 (59.38, 104.95)	43.51 (30.38, 54.07)
	Both	512.66 (416.01, 616.36)	571.02 (456.65, 703.25)	431.14 (330.35, 522.69)	317.34 (247.26, 379.01)	246.29 (191.82, 294.94)	172.7 (132.58, 210.06)	82.16 (61.68, 100.08)
Deaths (95% UI)	Male	178.45 (155.66, 201.99)	199.97 (171.03, 231.34)	189.26 (164.82, 215.44)	139.96 (120.61, 157.67)	102.08 (87.92, 114.09)	66.93 (54.63, 73.83)	29 (23.74, 32.93)
	Female	121.28 (93.78, 138.94)	130.84 (96.45, 152.7)	147.69 (108.44, 174.12)	106.8 (81.21, 125.57)	86.49 (64.84, 100.41)	62.98 (47.41, 74.67)	32.16 (23.71, 38.21)
	Both	299.74 (257.69, 330.48)	330.82 (275.2, 372.12)	336.95 (278.78, 376.58)	246.76 (206.56, 274.23)	188.57 (156.26, 209.99)	129.92 (106.89, 148.21)	61.17 (48.56, 70.02)
DALYs (95% UI)	Male	5220.88 (4545.95, 5905.34)	4941.81 (4221.05, 5721.31)	3839.81 (3346.83, 4365.2)	2276.46 (1963.52, 2563.24)	1303.59 (1124.84, 1454.31)	683.16 (557.49, 755.37)	259.19 (212.64, 294.08)
	Female	3545.85 (2738.98, 4061.72)	3228.27 (2381.35, 3763.71)	2992.58 (2188.96, 3524.52)	1730.44 (1316.88, 2035.34)	1100.49 (824.39, 1279.2)	640.63 (484.11, 759.81)	287.02 (212.5, 341.14)
	Both	8766.73 (7527.25, 9675.18)	8170.09 (6794.13, 9180.88)	6832.39 (5647.85, 7627.49)	4006.9 (3352.35, 4444.76)	2404.07 (1992.12, 2679.61)	1323.79 (1091.5, 1504.87)	546.2 (435.03, 624.04)

*DALYs* Disability-Adjusted Life Years

### Global trends by SDI quintiles

**Fig. 4:**
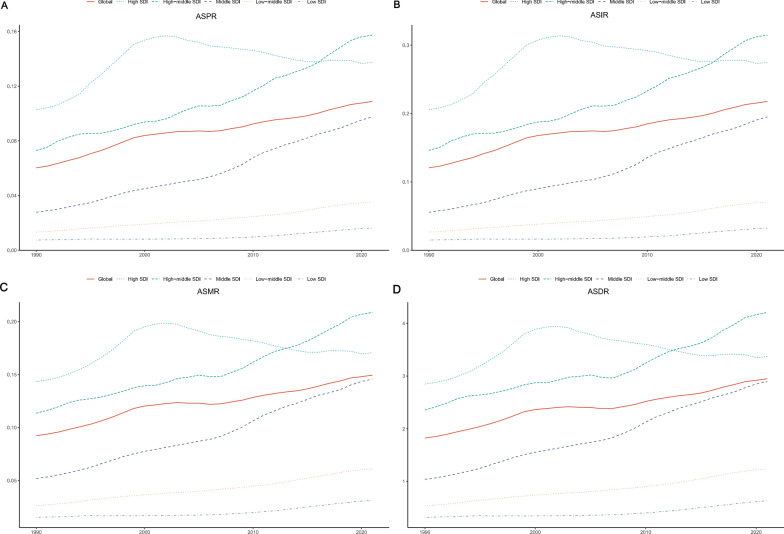
Global trends in age-standardised prevalence (**A**), incidence (**B**), mortality (**C**), and DALYs rates (**D**) from 1990 to 2021, stratified by SDI quintiles. DALYs, Disability-adjusted Life-Years; SDI: Socio-demographic Index

Between 1990 and 2021, the age-standardised prevalence, incidence, mortality, and DALY rates for neuroblastoma and peripheral nervous system tumours among the elderly across all SDI regions demonstrated broadly similar trends. Low SDI regions consistently maintained the lowest levels, while all other SDI regions, except for high SDI regions which showed minimal change, exhibited a gradual upward trend. The middle SDI regions experienced the highest growth rate throughout the period (Fig. 4A–D). In terms of age-standardised prevalence and incidence rates, high SDI regions held the highest levels until 2017, after which high-middle SDI regions surpassed them and took the lead. Regarding the rate of increase, middle SDI regions showed the highest EAPC at 4.22 (95% CI 4.09, 4.35), followed by middle-low SDI regions and then low SDI regions (Fig. 4A, B, Table 1, Supplementary Table 1). For age-standardised mortality rates, high SDI regions remained at the highest level until 2014, after which high-middle SDI regions took over. The highest growth rate in age-standardised mortality was observed in middle SDI regions, with an EAPC of 3.46 (95% CI 3.36, 3.56), followed by middle-low SDI regions and low SDI regions (Fig. 4C, Supplementary Table 2). With respect to age-standardised DALY rates, high SDI regions maintained the highest levels until 2013, after which high-middle SDI regions consistently led. The middle SDI regions again displayed the highest growth rate, with an EAPC of 3.42 (95% CI 3.32, 3.53), followed by middle-low SDI regions and low SDI regions (Fig. 4D, Supplementary Table 3).

### Regional trends

**Fig. 5:**
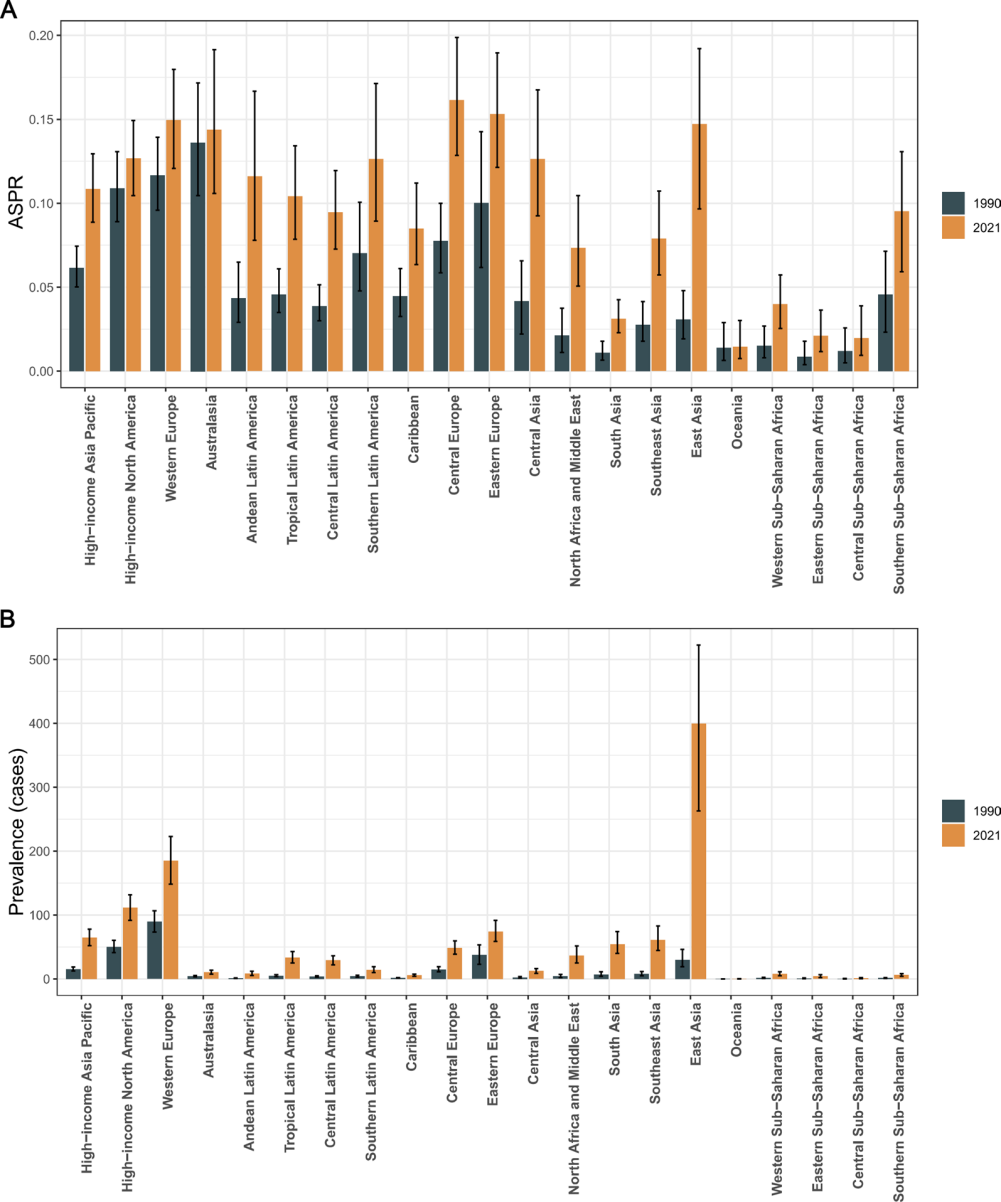
Age-standardised prevalence rates (**A**) and prevalent cases (**B**) of neuroblastoma and peripheral nervous system tumours among people aged 60 and above across the 21 regions of the Global Burden of Disease (GBD) from 1990 to 2021

The GBD regional classification includes 204 countries and territories, grouped into 21 regions [15]. An analysis of the burden of neuroblastoma and peripheral nervous system tumours among the elderly across 21 global regions in 2021 revealed that Central Europe exhibited the highest age-standardized rates for prevalence, incidence, and mortality, recorded at 0.16 per 100,000 (95% UI 0.13, 0.20), 0.32 per 100,000 (95% UI 0.26, 0.40), and 0.21 per 100,000 (95% UI 0.18, 0.25), respectively. Meanwhile, Eastern Europe showed the highest age-standardised DALY rate globally, reaching 4.58 per 100,000 (95% UI 3.91, 5.29) (Fig. 5A, Supplementary Fig. 1A, Supplementary Fig. 2A, Supplementary Fig. 3A, Table 1, Supplementary Tables 1–3). In terms of case numbers, East Asia recorded the highest numbers for prevalence, incidence, mortality, and DALYs in 2021 among the 21 regions, with figures of 399.83 (95% UI 262.84, 522.80), 799.66 (95% UI 525.67, 1045.61), 532.57 (95% UI 367.41, 670.62), and 10,742.81 (95% UI 7,395.77, 13,606.59), respectively (Fig. 5B, Supplementary Figs. 1B, 2B, 3B, Table 1, Supplementary Tables 1–3). Between 1990 and 2021, most regions exhibited an upward trend in age-standardised prevalence and incidence rates, except for Australasia and Oceania, where no significant increases were observed. East Asia demonstrated the fastest growth, with EAPCs of 5.89 (95% CI 5.52, 6.25) for both prevalence and incidence rates. Similarly, in terms of age-standardised mortality and DALY rates, most regions showed a marked increase, with East Asia again exhibiting the highest growth. The EAPCs for age-standardised mortality and DALY rates in East Asia were 4.86 (95% CI 4.55, 5.18) and 4.82 (95% CI 4.49, 5.15), respectively (Fig. 1A–D).

### National trends

**Fig. 6:**
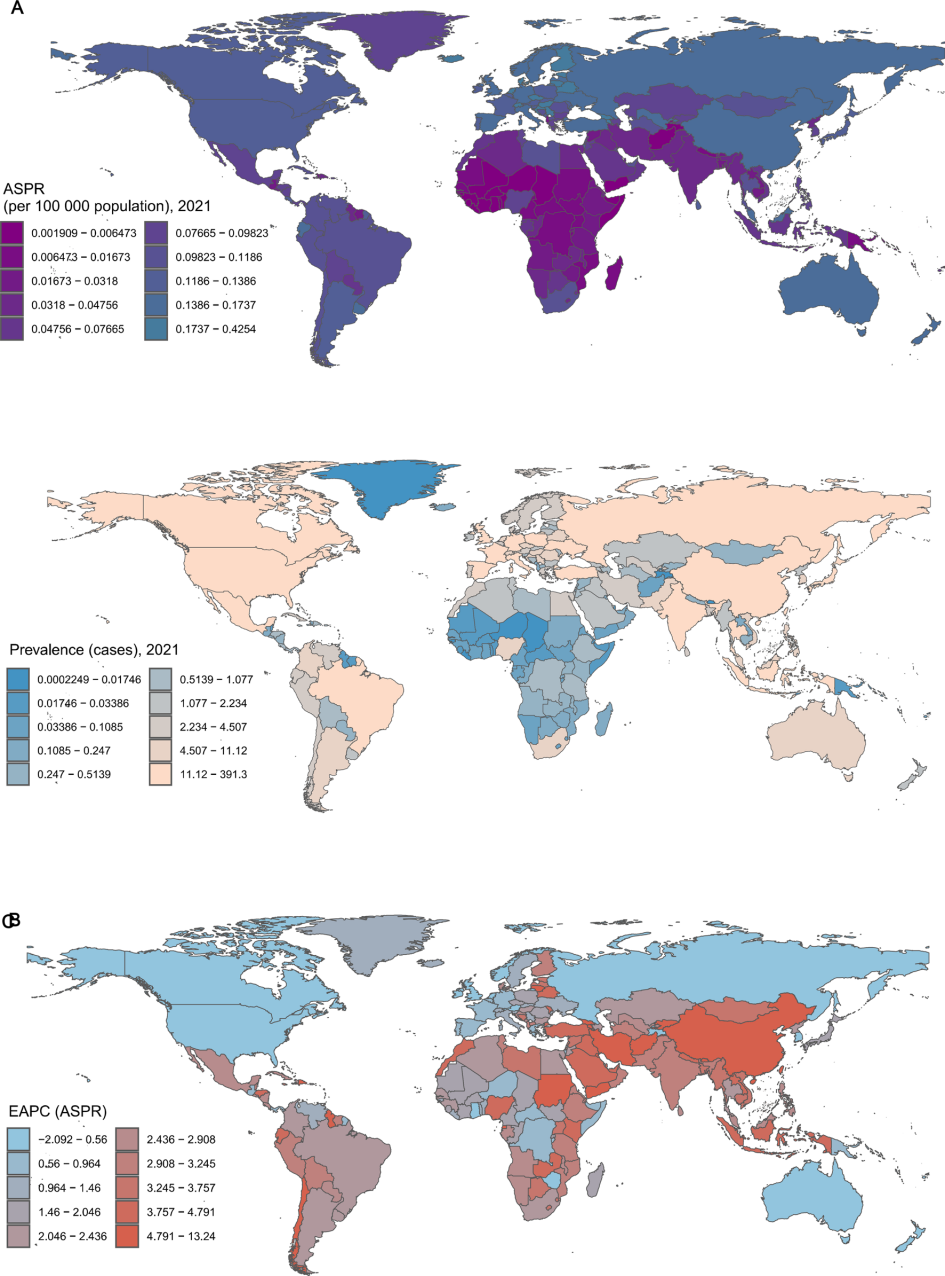
The global prevalence of neuroblastoma and peripheral nervous system tumours among individuals aged 60 years and older across 204 countries and territories: age-standardised prevalence rate in 2021 (**A**), number of prevalent cases in 2021 (**B**), and the EAPC in prevalence from 1990 to 2021 (**C**). EAPC, Estimated Average Percentage Change

On a national scale, in 2021, the Republic of Estonia recorded the highest age-standardised prevalence, incidence, and mortality rates globally, at 0.42 per 100,000 (95% UI 0.28, 0.63), 0.85 per 100,000 (95% UI 0.56, 1.25), and 0.51 per 100,000 (95% UI 0.34, 0.72), respectively. In contrast, the Republic of Croatia had the highest age-standardised DALY rate at 9.14 per 100,000 (95% UI 6.36, 12.73) (Fig. 6A, Supplementary Figs. 4A, 5A, Supplementary Tables 4–8). In 2021, the People's Republic of China reported the highest numbers for prevalence, incidence, mortality, and DALYs among the 204 countries studied, with figures of 389.19 (253.48, 511.35), 778.39 (95% UI 506.97, 1022.69), 518.98 (95% UI 354.83, 656.37), and 10,464.64 (95% UI 7,140.82, 13,312.97), respectively (Fig. 6B, Supplementary Figs. 4B, 5B, 6B, Supplementary Tables 4–8). Examining trends from 1990 to 2021 across 204 countries, more than 90% exhibited an increasing pattern in age-standardised prevalence and incidence rates. Georgia exhibited the highest growth rate globally, with EAPCs for age-standardised prevalence and incidence rates of 13.35 (95% CI 11.93, 14.80). In terms of age-standardised mortality and DALY rates, over 85% of countries demonstrated an increasing trend, with Georgia again showing the highest growth. The EAPCs for age-standardised mortality and DALY rates in Georgia were 13.34 (95% UI 11.89, 14.80) and 13.26 (95% CI 11.81, 14.73), respectively (Fig. 6C, Supplementary Figs. 4C, 5C, 6C).

## Discussion

The probability of developing neurological tumours increases significantly in the elderly due to factors such as reduced cellular regeneration capacity, the build-up of genetic alterations, the decline of immune system function, and prolonged exposure to environmental and lifestyle risk factors. Neuroblastoma and peripheral nervous system tumours exemplify this phenomenon, exhibiting distinctive patterns of onset and clinical presentation [16–19]. This is the first comprehensive study to detail the global, regional, and national prevalence, incidence, mortality, and DALY rates of neuroblastoma and peripheral nervous system tumours among the elderly between 1990 and 2021. Our analysis segments the distribution of these tumors across regions with different SDI levels, areas, countries, genders, and age groups, providing crucial insights into the status of neuroblastoma and peripheral nervous system tumour burden among older adults worldwide. This study underscores the pressing demand for more powerful and targeted health interventions to mitigate the burden of these tumours among the elderly [20].

### Increasing global burden

Our findings indicate that the burden of neuroblastoma and peripheral nervous system tumours has shown a significant upward trend from 1990 to 2021. The age-standardised prevalence and incidence rates increased from 0.06 per 100,000 (95% UI 0.05, 0.08) and 0.12 per 100,000 (95% UI 0.09, 0.15) to 0.11 per 100,000 (95% UI 0.09, 0.13) and 0.22 per 100,000 (95% UI 0.17, 0.26), respectively. Similarly, the age-standardised mortality and DALY rates also displayed a notable increase, rising from 0.09 per 100,000 (95% UI 0.08, 0.11) and 1.82 per 100,000 (95% UI 1.55, 2.12) to 2.95 per 100,000 (95% UI 2.47, 3.29). This upward trend highlights the growing impact of neuroblastoma and peripheral nervous system tumours on the global elderly population (Fig. 6).

The study suggests that this trend may be closely linked to a combination of external and internal factors, including genetic susceptibility, environmental exposure, and lifestyle changes. For instance, exposure to certain chemicals and pollutants is believed to be associated with tumour development, and changes in dietary patterns may also influence disease risk. With a rise in the consumption of processed foods and greater exposure to harmful substances in daily life, these cumulative factors merit further attention [21–24]. Additionally, the ageing population is more susceptible to these factors due to physiological decline and alterations in immune function, which contribute to the rising incidence of neuroblastoma and peripheral nervous system tumours [25–27]. Future research should focus on investigating the potential links between these external environmental factors and neurological tumours, as well as the unique role that the elderly population may play in this process. Additionally, studies should explore the relationship between neurodegenerative diseases and these tumours to determine whether there are shared pathogenic mechanisms, thereby paving the way for more effective preventive and therapeutic strategies [28].

### Regional disparities

Our findings reveal significant regional disparities in the burden of neuroblastoma and peripheral nervous system tumours among the elderly population. In this study, we observed that Central Europe exhibited the highest age-standardised prevalence, incidence, and mortality rates, while Eastern Europe had the highest age-standardised DALY rate, indicating that these two regions bear a heavy disease burden. In terms of the rate of increase in age-standardised prevalence, incidence, mortality, and DALY rates, East Asia showed the highest levels, suggesting that the burden in East Asia has been escalating rapidly over the past three decades. These regional differences may be attributed to a combination of genetic, environmental, healthcare, and lifestyle factors [25, 29]. The disease burden in Central and Eastern Europe may stem from multiple interacting factors. Genetic susceptibility plays a key role, with certain genetic mutations or familial aggregation linked to higher disease rates in these populations. Additionally, early industrialisation exposed these regions to greater environmental pollutants, such as heavy metals and chemicals, which contribute to an elevated risk of neuroblastoma and other neurological tumours. The accessibility and quality of healthcare resources also contribute, with uneven distribution between urban and rural areas hindering early diagnosis and treatment, which increases mortality rates [30–32].

Behavioral factors including elevated smoking rates, alcohol intake, and poor eating habits further contribute to the cancer burden in these regions. Research indicates that lifestyle changes have directly impacted cancer incidence, particularly in populations with limited health education and resources [33–37]. Regarding the situation in East Asia, we observed that the region exhibited the highest growth in age-standardised prevalence, incidence, mortality, and DALY rates. This trend may be related to rapid economic development, accelerated urbanisation, and lifestyle changes in the region [38]. In the last thirty years, shifts in eating habits and lifestyle behaviors and environmental factors in East Asia may have contributed to the increased disease burden [39, 40].

### Socio-demographic variations

Our study found that in 2021, regions with medium–high SDI had the highest age-standardised prevalence, incidence, mortality, and DALY rates for neuroblastoma and peripheral nervous system tumours among the elderly, followed by high SDI regions. This highlights that regions with higher SDI carry a greater disease burden. While high SDI regions often have more advanced medical facilities, enabling better cancer screening and early diagnosis, this may also result in higher reported incidence rates, as more cases are detected. Thus, the higher disease burden in these regions may reflect improved detection rather than higher true incidence. According to an analysis from the GLOBOCAN database, elderly populations in high-income countries are typically more efficient in early screening and diagnosis of neurological tumours [1, 41]. However, this efficiency in healthcare does not necessarily translate into lower disease rates but may rather highlight the more effective detection of cases that may otherwise go undiagnosed. Additionally, these regions often exhibit more diverse lifestyles, including unhealthy dietary habits, a lack of physical activity, and high smoking rates, all of which are closely linked to cancer incidence. Research indicates that lifestyle changes in high-income countries have led to an increase in cancer rates [42]. Furthermore, the rapid industrialisation and urbanisation seen in high SDI regions have led to increased environmental pollution, including air and water contamination, which are known cancer risk factors [43].

Conversely, medium SDI regions showed the most significant growth in disease burden. This may be due to economic transitions and rapid urbanisation, which exacerbate environmental factors like pollution. These regions still face challenges in healthcare access and early diagnosis, leading to higher incidence and mortality rates. Furthermore, socio-economic inequalities may impede residents’ ability to access quality healthcare, further exacerbating the disease burden [43]. The ageing process in medium SDI regions may also be accelerating faster than in other SDI categories, which makes the disease burden more pronounced in elderly populations. Moreover, in medium SDI regions, the westernisation of lifestyles driven by rapid economic development has resulted in widespread unhealthy eating habits and an increased risk of obesity and related diseases, directly contributing to the rise in cancer incidence [33, 34].

### Trends in different age groups

Our study analysed age-related disease trends in neuroblastoma and peripheral nervous system tumours among the elderly, indicating that the incidence and impact patterns vary across different age groups. Among these, people aged 90–94 had the highest rates of prevalence, incidence, and mortality, whereas individuals between 70 and 74 years old had the highest DALY rates for their age group. These results emphasize that, with increasing age, the disease burden of neuroblastoma and peripheral nervous system tumours shows a clear upward trend in the elderly population.

Some literature suggests that the incidence of neuroblastoma and peripheral nervous system tumours rises significantly among the oldest age groups, particularly those aged over 90 [24]. Earlier studies have shown that the decline in immune surveillance is vital to the progression of neurological tumours as individuals age. Factors such as immunosenescence, reduced T-cell function, chronic inflammation, and cumulative DNA damage contribute to the increased susceptibility of the elderly to neuroblastoma and peripheral nervous system tumours [44]. The progressive loss of immune system efficiency with aging leads to a diminished capacity to detect and eliminate malignant cells, resulting in an increased risk of tumour development. Additionally, genetic mutations accumulated over a lifetime may compound the risk, as older individuals are more likely to have mutations that drive tumour growth. These biological changes, in conjunction with environmental exposures, lead to more pronounced diagnoses and mortality rates in the oldest age groups [45–48]. We also observed that individuals aged 70–74 experienced a notable burden in terms of DALYs. The deterioration of survival and quality of life associated with peripheral nervous system tumours is particularly prominent in older age groups. Earlier studies have shown that, although the overall mortality rate for those aged 70–74 may not be as significant as in older age groups, the disability burden remains substantial. This may be due to a higher prevalence of chronic conditions or multimorbidity within this age group. Additionally, elderly individuals diagnosed with neuroblastoma and peripheral nervous system tumours may face psychological issues such as depression and anxiety. Functional loss and dependence on others as a result of these diseases can lead to feelings of loneliness, helplessness, and even a loss of interest in life, leading to a marked reduction in the quality of life for these individuals [49–51].

## Gender differences

Our findings revealed that among patients aged 60 and above, the global age-standardised prevalence rate for males was 1.56 times greater than for females, while the age-standardised incidence, mortality, and DALY rates were 1.65, 1.58, and 1.59 times higher, respectively. Form 1990 to 2021, the EAPC for neuroblastoma and peripheral nervous system tumours in males significantly surpassed that of females, indicating a much higher disease burden among older men globally.

This observed gender disparity may result from a mixture of age-related biological and socio-behavioral factors. Hormonal shifts and immune system changes in older males may contribute to their heightened susceptibility to these tumours [52, 53]. Specifically, the weakening of immune function as individuals age could reduce the body's capacity to detect and fight tumour cells, while hormonal changes, particularly in testosterone levels, may affect tumour development and progression [54–57]. Additionally, gender-related delays in seeking medical care and differences in treatment approaches may further exacerbate the observed disparity [58–60]. Understanding these age-modified gender differences is crucial for developing tailored healthcare strategies to address the unique needs of elderly male patients.

### Need for effective interventions

With the intensifying trend of global ageing, the tumour burden among the elderly, particularly neuroblastoma and peripheral nervous system tumours, has become a significant public health challenge for the future. According to the World Population Prospects report published by the United Nations in 2020, the number of people aged 60 and older worldwide is projected to surpass 2 billion by 2050, projecting a more severe burden of neuroblastoma and peripheral nervous system tumours. Although these tumours are more commonly seen in adolescents and children, they are also showing a steady increase in the elderly population as society ages. While current treatments have shown some effectiveness in certain cases, traditional methods often have considerable limitations due to the biological characteristics of these tumours and the unique physiological conditions of older patients, highlighting the urgent need for new, more effective intervention measures. For the elderly population, conventional treatments are often poorly tolerated due to declining immune and metabolic functions, leading to suboptimal outcomes and significant adverse effects. This is particularly true for neuroblastoma, a tumour originating in the sympathetic nervous system, where traditional treatments are often insufficient in addressing issues related to metastasis and recurrence. Therefore, we recommend personalised precision treatment strategies involving the detection of specific gene mutations or related molecular markers in tumour tissue. For instance, drugs targeting ALK (Anaplastic Lymphoma Kinase) mutations can be selectively used to inhibit tumour growth and reduce the side effects associated with traditional chemotherapy and radiotherapy [61, 62]. We suggest that high-SDI regions accelerate research into targeted therapies and immunotherapies, particularly focusing on the needs of elderly patients. Meanwhile, in lower-SDI regions, there is a need to enhance the development of imaging technologies (e.g., CT, MRI) and biomarkers, especially in identifying tumour-specific markers that can provide more tools for early clinical screening. In resource-limited areas, strengthening training for primary care physicians and promoting health check-ups can help increase the likelihood of early detection.

### Limitations

There are several limitations to this study. First, each iteration of the GBD data differs from previous generations, making them incomparable. These discrepancies are likely due to improvements in algorithms and models over time [63]. Second, an accurate global assessment of the burden of neuroblastoma and peripheral nervous system tumours in the elderly requires more precise epidemiological data, as the databases for neuroblastoma and peripheral nervous system tumours in some countries are still incomplete [1], which may lead to inaccuracies in estimates based on complex statistical models. Third, the GBD does not differentiate between subtypes of neuroblastoma and peripheral nervous system tumours or the severity of the condition, limiting our ability to analyse the specific burden of these subtypes on a global scale and provide detailed epidemiological insights based on the severity of patients' conditions. Fourth, in regions with lower levels of healthcare, the lack of expertise in medical examinations and advanced neuroimaging technologies may hinder the accurate differentiation of neuroblastoma and peripheral nervous system tumours from other diseases (such as kidney tumours, liver tumours, and metastatic cancers), increasing diagnostic uncertainty in these areas.

## Conclusion

In conclusion, since the 1990s, the incidence of neuroblastoma and peripheral nervous system tumours among the elderly has steadily increased in almost every country, with notable variations observed among regions, genders, and age categories. The treatment of neuroblastoma and peripheral nervous system tumours continues to present considerable challenges, highlighting the critical demand for more efficient and focused interventions to alleviate the burden of these central nervous system tumours. To address the disparities in disease incidence by region, gender, and age, a comprehensive approach integrating medical, psychosocial, and public health strategies is essential. Future studies should prioritize identifying possible risk factors and creating innovative treatment methods to address this prevalent and significant disease.

## Supplementary Information

Supplementary Material 1

Supplementary Material 2

Supplementary Material 3

Supplementary Material 4

Supplementary Material 5

Supplementary Material 6

Supplementary Material 7

Supplementary Material 8

Supplementary Material 9

Supplementary Material 10

Supplementary Material 11

Supplementary Material 12

Supplementary Material 13

Supplementary Material 14

## Data Availability

Data used for the analyses are publicly available from the Institute of Health Metrics and Evaluation (https://vizhub.healthdata.org/gbd-results/).

## Abbreviations

ASR: Age-standardized rate
ALK: Anaplastic Lymphoma Kinase
DALYs: Disability-adjusted life years
EAPC: Estimated Average Percentage Change
GBD: Global Burden of Disease
GHDx: Global Health Data Exchange
ICD-10: International Classification of Diseases, 10th Edition
SDI: Socio-demographic Index
UI: Uncertainty intervals
CI: Confidence Intervals
