# Supplementary material for: Global, regional, and national burden of neuroblastoma and peripheral nervous system tumours in individuals aged over 60 from 1990 to 2021: a trend analysis of global burden of disease study
Source: J Health Popul Nutr. 2025 Mar 17;44:78. doi: 10.1186/s41043-025-00810-9 (PMC11916991; doi:10.1186/s41043-025-00810-9)
Supplement: Supplementary file 1 — Supplementary Material 1 [file 41043_2025_810_MOESM1_ESM.docx]

List of Appendix and figures

Appendix Figures

**Supplement figure 1.** Age-standardised incidence rates (A) and incidence cases (B) of neuroblastoma and peripheral nervous system tumours among individuals aged 60 years and older across the 21 regions of the Global Burden of Disease (GBD) from 1990 to 2021.

**Supplement figure 2.** Age-standardised mortality rates (A) and deaths (B) of neuroblastoma and peripheral nervous system tumours among individuals aged 60 years and older across the 21 regions of the Global Burden of Disease (GBD) from 1990 to 2021.

**Supplement figure 3.** Age-standardised DALY rates (A) and the number of DALYs (B) of neuroblastoma and peripheral nervous system tumours among individuals aged 60 years and older across the 21 regions of the Global Burden of Disease (GBD) from 1990 to 2021.

DALY, Disability-adjusted Life-Year.

**Supplement figure 4.** The global prevalence of neuroblastoma and peripheral nervous system tumours among individuals aged 60 years and older across 204 countries and territories: age-standardised incidence rate in 2021 (A), number of incidence cases in 2021 (B), and the EAPC in incidence from 1990 to 2021 (C).

EAPC, Estimated Average Percentage Change.

**Supplement figure 5.** The global prevalence of neuroblastoma and peripheral nervous system tumours among individuals aged 60 years and older across 204 countries and territories: age-standardised mortality rate in 2021 (A), deaths in 2021 (B), and the EAPC in mortality from 1990 to 2021 (C).

EAPC, Estimated Average Percentage Change.

**Supplement figure 6.** The global prevalence of neuroblastoma and peripheral nervous system tumours among individuals aged 60 years and older across 204 countries and territories: age-standardised DALYs rate in 2021 (A), the number of DALYs in 2021 (B), and the EAPC in DALYs from 1990 to 2021 (C).

EAPC, Estimated Average Percentage Change; DALYs, Disability-adjusted life–years.
