# Supplementary figures and images for: Global, regional, and national burden of neuroblastoma and peripheral nervous system tumours in individuals aged over 60 from 1990 to 2021: a trend analysis of global burden of disease study
Source: J Health Popul Nutr. 2025 Mar 17;44:78. doi: 10.1186/s41043-025-00810-9 (PMC11916991; doi:10.1186/s41043-025-00810-9)

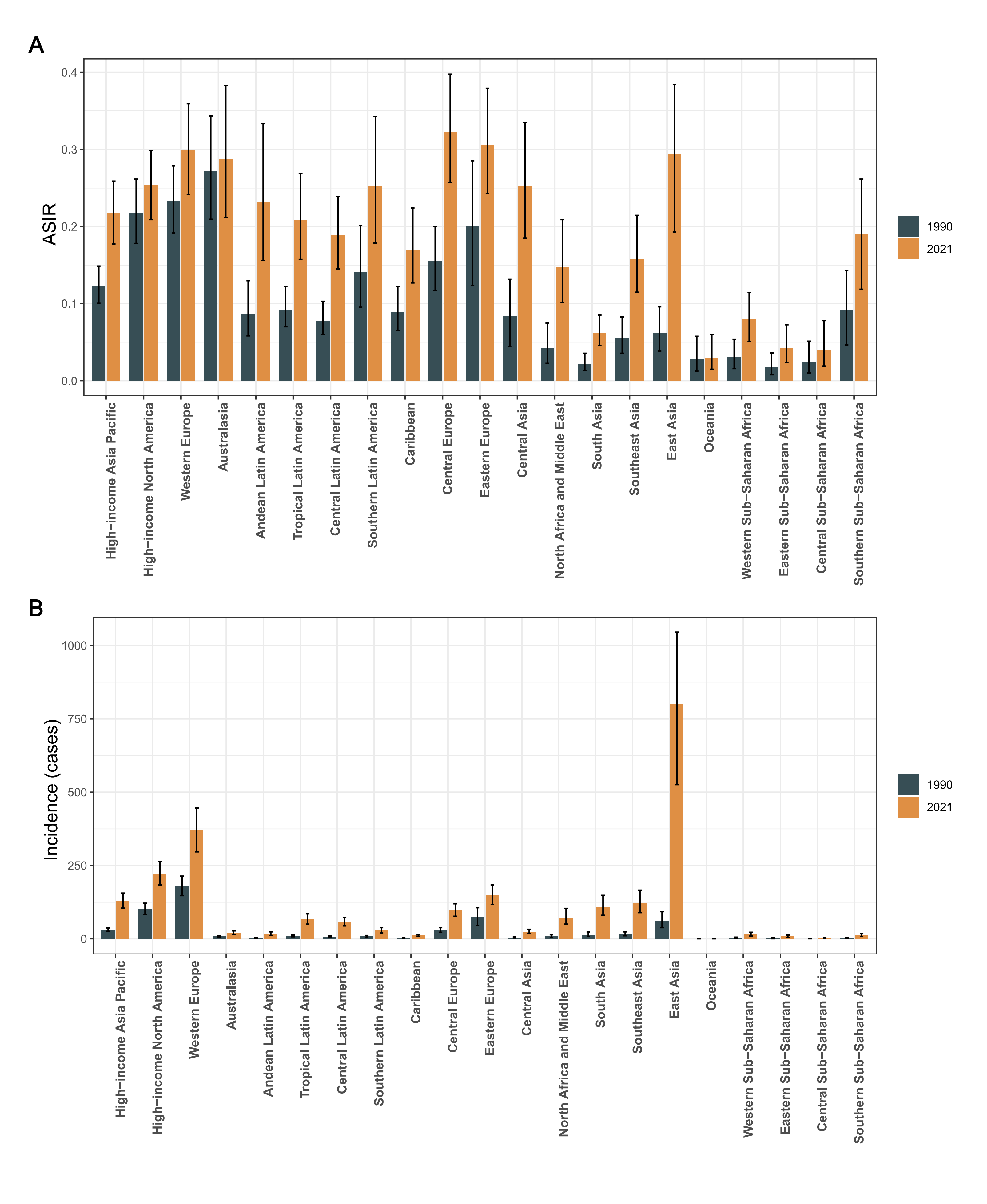

Supplement: Supplementary file 2 — Supplementary Material 2 [file 41043_2025_810_MOESM2_ESM.tif]

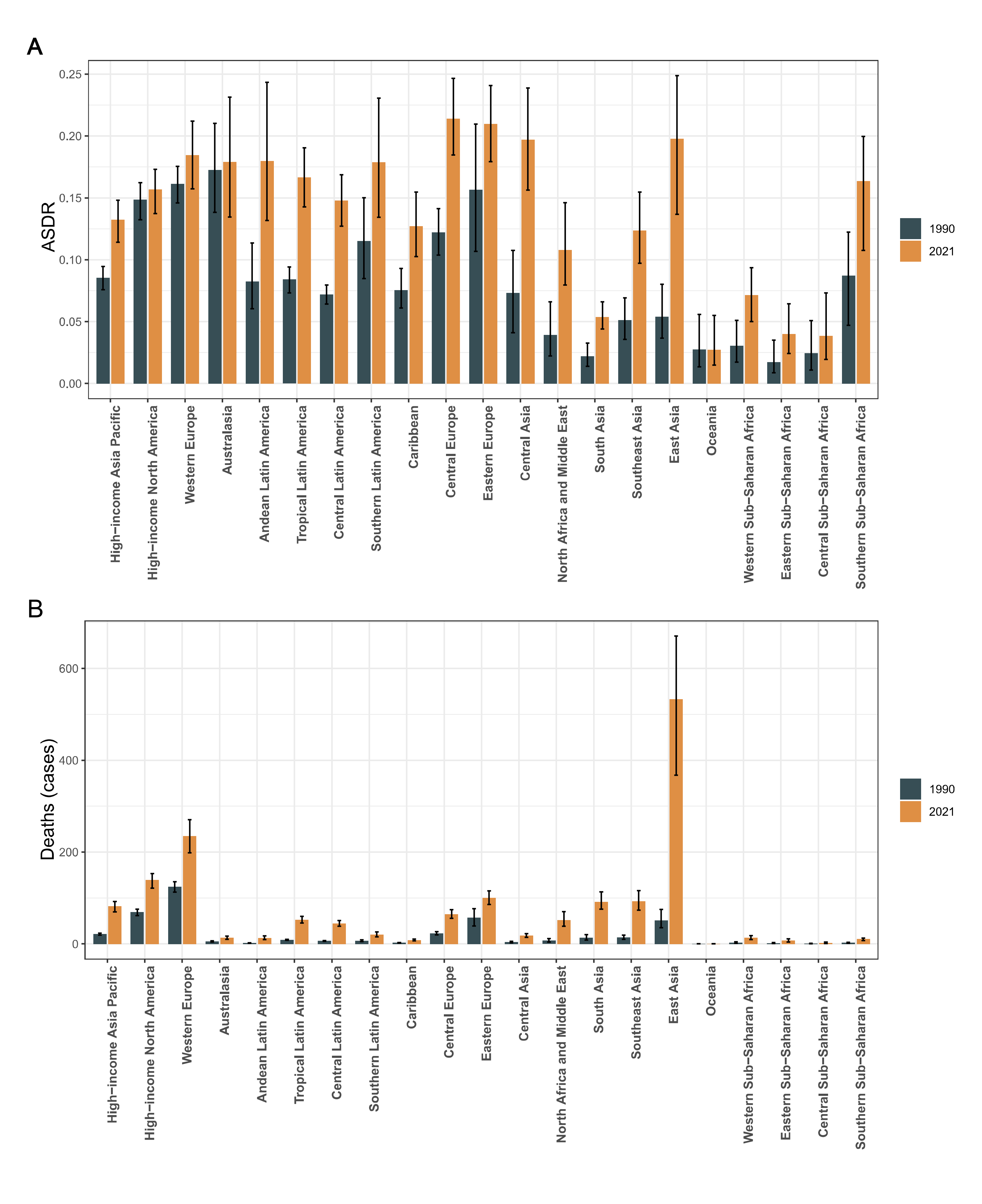

Supplement: Supplementary file 3 — Supplementary Material 3 [file 41043_2025_810_MOESM3_ESM.tif]

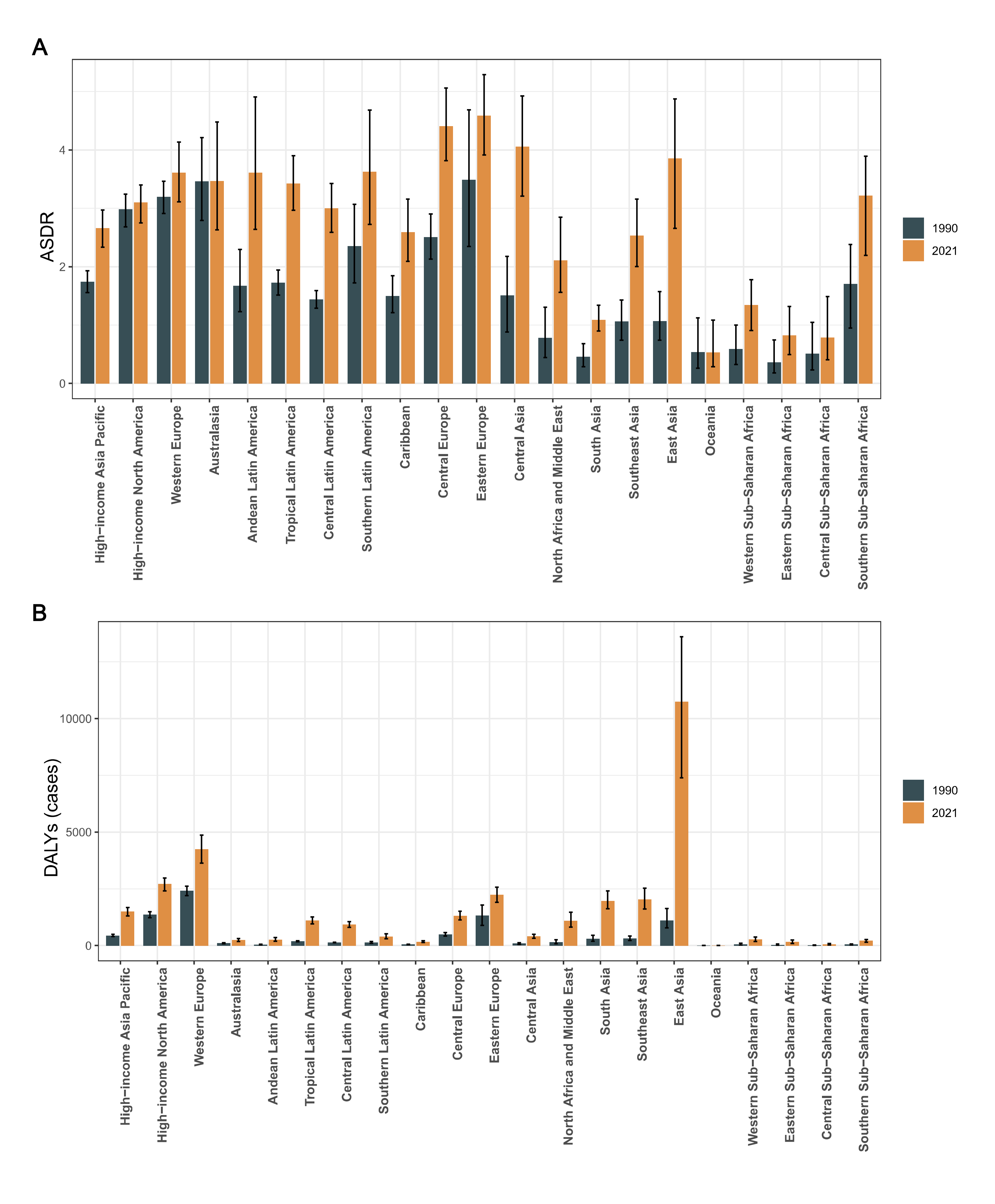

Supplement: Supplementary file 4 — Supplementary Material 4 [file 41043_2025_810_MOESM4_ESM.tif]
