# Supplementary material for: Global, regional, and national burden of neuroblastoma and peripheral nervous system tumours in individuals aged over 60 from 1990 to 2021: a trend analysis of global burden of disease study
Source: J Health Popul Nutr. 2025 Mar 17;44:78. doi: 10.1186/s41043-025-00810-9 (PMC11916991; doi:10.1186/s41043-025-00810-9)
Supplement: Supplementary file 8 — Supplementary Material 8 [file 41043_2025_810_MOESM8_ESM.docx]

Supplement 1. The age-standardized incidence rate, number of cases, and EAPC of central nervous system cancers among elderly individuals aged 60 and above globally and regionally from 1990 to 2021.

|  | Incidence (95% UI) | | | | |
| --- | --- | --- | --- | --- | --- |
|  | Cases in 1990 (million) | Age-standardised rate in 1990 (per 100 000) | Cases in 2021(million) | Age-standardised rate in 2021 (per 100 000) | EAPC (95% CI) |
|  |  |  |  |  |  |
| Global | 56432.8(50032.7,62202.7) | 11.7(10.3,12.9) | 164644.6(142850.4,184409.7) | 15.3(13.1,17.2) | 0.9(0.9,1.0) |
| Sex |  | | | | |
| Male | 39.5(33.6,41.2) | 13.3(11,15.7) | 48.4(36.5,46.3) | 17.3(13.9,20.5) | 0.9(0.9,1.0) |
| Female | 30.7(26.5,32) | 10.4(9.4,11.5) | 38.4(28.7,36.3) | 13.6(11.6,15.4) | 0.9(0.8,1.0) |
| SDI quintile |  | | | | |
| High | 24163.1(22882.8,25045.2) | 16.7(15.8,17.4) | 59472(53687.1,63398.4) | 21.1(19.1,22.7) | 0.8(0.7,0.8) |
| High middle | 16309.3(14252.1,17974) | 12.9(11.2,14.4) | 48040.5(40036.9,55040.3) | 18.8(15.6,21.7) | 1.4(1.2,1.5) |
| Middle | 11458.5(9186.6,14157.3) | 9.6(7.7,11.9) | 42455.8(34426.4,51341.4) | 13.1(10.5,15.9) | 1.1(1.0,1.1) |
| Low middle | 3458.6(2577.5,4719.2) | 4.9(3.7,6.8) | 12016(10024.1,15224.2) | 7(5.8,8.9) | 1.2(1.1,1.3) |
| Low | 973.3(609,1334.9) | 3.7(2.3,5.1) | 2483.7(1721.9,3113.2) | 4.4(3,5.5) | 0.5(0.4,0.6) |
| GBD region |  | | | | |
| Andean Latin America | 185.7(141.8,249.7) | 7.9(5.8,10.9) | 942.5(724.7,1182.1) | 13.2(9.9,17) | 1.8(1.5,2.1) |
| Australasia | 652.2(604,697.3) | 20.8(18.9,22.8) | 1452.5(1270.9,1634.8) | 20.5(17.2,23.9) | -0.1(-0.2,0.0) |
| Caribbean | 263.9(235,300.9) | 8.2(7.2,9.5) | 834.4(719.8,958.2) | 12.4(10.5,14.7) | 1.9(1.7,2.0) |
| Central Asia | 400.1(313.6,472.8) | 6.9(5.4,8.2) | 1249.9(1094,1411.3) | 12.4(10.9,14) | 2.2(2.1,2.4) |
| Central Europe | 2796.1(2639.2,3018.2) | 14(13.1,15.2) | 7139.3(6465.3,7786.6) | 23.6(21.3,26) | 1.7(1.3,2.0) |
| Central Latin America | 615.8(585.9,641.4) | 6.5(6.1,6.8) | 2896.4(2547.4,3239.2) | 9.5(8.3,10.6) | 0.8(0.5,1.2) |
| Central Sub-Saharan Africa | 71.7(49,98.8) | 2.9(1.9,4.1) | 206.5(130.4,284.7) | 3.6(2.2,5.1) | 0.8(0.7,0.9) |
| East Asia | 12774.6(9764.4,16271.7) | 12.6(9.6,16.1) | 47451(35514.8,60074.2) | 17.4(12.7,22.2) | 1.1(1.1,1.2) |
| Eastern Europe | 2482.1(2245.8,2722.7) | 6.5(5.8,7.2) | 7198.7(6582.6,7837.4) | 14.9(13.5,16.3) | 3.0(2.8,3.1) |
| Eastern Sub-Saharan Africa | 320.6(191.2,408.1) | 3.7(2.2,4.7) | 831.2(522.6,1029.4) | 4.4(2.8,5.5) | 0.6(0.6,0.6) |
| High-income Asia Pacific | 2133.4(1872,2347.1) | 8.6(7.2,9.8) | 10641.1(8585.1,12272.5) | 15.9(12.4,19.1) | 2.1(1.8,2.4) |
| High-income North America | 10031(9389.1,10445.7) | 21.5(20.1,22.5) | 21262.7(19159.8,22522.2) | 23.8(21.5,25.5) | 0.3(0.2,0.4) |
| North Africa and Middle East | 2586.1(1968.4,3748.7) | 13.7(10.1,19.9) | 8830.6(6463.3,11053.1) | 17.5(12.6,22) | 1.1(1.0,1.2) |
| Oceania | 5.7(2.9,8.3) | 1.9(1,2.9) | 15.2(7.9,21.9) | 2(1,2.9) | 0.2(0.1,0.2) |
| South Asia | 2980.4(1951.7,3957.6) | 4.5(2.9,6) | 9494(7682.9,12860.2) | 5.3(4.2,7.1) | 0.3(0.2,0.4) |
| Southeast Asia | 1707.5(1273,2156) | 5.9(4.4,7.6) | 5796.7(4361.1,7152.4) | 7.5(5.6,9.3) | 0.8(0.7,1.0) |
| Southern Latin America | 639.4(555,736.1) | 10.9(9.1,12.9) | 1477.1(1311.9,1628) | 13(11.3,14.9) | 1.2(0.8,1.5) |
| Southern Sub-Saharan Africa | 160.4(109.7,203.2) | 5.2(3.5,6.6) | 461(308.2,542.8) | 6.9(4.5,8.2) | 1.0(0.9,1.1) |
| Tropical Latin America | 1358.8(1240.5,1503) | 12.7(11.4,14.1) | 6343.7(5776.1,6758.5) | 19.8(17.9,21.4) | 1.6(1.4,1.8) |
| Western Europe | 14175.3(13394,14769.1) | 18.7(17.6,19.6) | 29819.1(26787,32012.3) | 24.6(22.2,26.6) | 1.0(0.8,1.1) |
| Western Sub-Saharan Africa | 91.8(66,118.7) | 0.9(0.6,1.2) | 301.1(185.1,375.5) | 1.4(0.8,1.8) | 1.6(1.6,1.7) |

EAPC: Estimated annual percentage change
