# Supplementary material for: Global, regional, and national burden of neuroblastoma and peripheral nervous system tumours in individuals aged over 60 from 1990 to 2021: a trend analysis of global burden of disease study
Source: J Health Popul Nutr. 2025 Mar 17;44:78. doi: 10.1186/s41043-025-00810-9 (PMC11916991; doi:10.1186/s41043-025-00810-9)
Supplement: Supplementary file 9 — Supplementary Material 9 [file 41043_2025_810_MOESM9_ESM.docx]

Supplement 2. The age-standardized mortality rate, number of cases, and EAPC of neuroblastoma and peripheral nervous system tumours among elderly individuals aged 60 and above globally and regionally from 1990 to 2021.

|  | Deaths (95% UI) | | | | |
| --- | --- | --- | --- | --- | --- |
|  | Cases in 1990 (million) | Age-standardised rate in 1990 (per 100 000) | Cases in 2021(million) | Age-standardised rate in 2021 (per 100 000) | EAPC (95% CI) |
|  |  |  |  |  |  |
| Global | 424.42(361.82,491.51) | 0.09(0.08,0.11) | 1593.92(1329.94,1781.63) | 0.15(0.12,0.17) | 1.42(1.29,1.56) |
| Sex |  | | | | |
| Male | 215.08(181.58,246.01) | 0.11(0.09,0.12) | 905.66(778.42,1027.28) | 0.19(0.16,0.21) | 1.69(1.56,1.82) |
| Female | 209.34(174.56,255.80) | 0.08(0.07,0.10) | 688.27(515.84,804.62) | 0.12(0.09,0.14) | 1.08(0.93,1.24) |
| SDI quintile |  | | | | |
| High | 206.65(188.12,221.59) | 0.14(0.13,0.15) | 482.24(422.92,531.59) | 0.17(0.15,0.19) | 0.32(-0.02,0.67) |
| High middle | 138.34(107.95,173.23) | 0.11(0.09,0.14) | 529.27(417.17,619.18) | 0.21(0.16,0.24) | 1.89(1.80,1.99) |
| Middle | 57.81(43.90,75.12) | 0.05(0.04,0.07) | 463.89(371.43,540.93) | 0.15(0.12,0.17) | 3.46(3.36,3.56) |
| Low middle | 17.28(11.92,25.44) | 0.03(0.02,0.04) | 100.11(84.00,119.72) | 0.06(0.05,0.07) | 2.63(2.54,2.73) |
| Low | 3.75(1.88,7.33) | 0.02(0.01,0.03) | 16.78(10.65,25.77) | 0.03(0.02,0.05) | 2.25(1.91,2.58) |
| GBD region |  | | | | |
| Andean Latin America | 1.90(1.39,2.61) | 0.08(0.06,0.11) | 12.72(9.33,17.24) | 0.18(0.13,0.24) | 2.77(2.58,2.97) |
| Australasia | 5.31(4.27,6.48) | 0.17(0.14,0.21) | 12.94(9.69,16.76) | 0.18(0.13,0.23) | -0.14(-0.46,0.17) |
| Caribbean | 2.35(1.90,2.89) | 0.08(0.06,0.09) | 8.46(6.84,10.30) | 0.13(0.10,0.15) | 2.26(1.85,2.68) |
| Central Asia | 4.04(2.31,5.91) | 0.07(0.04,0.11) | 18.14(14.35,22.07) | 0.20(0.16,0.24) | 3.91(3.64,4.19) |
| Central Europe | 23.12(19.65,26.73) | 0.12(0.10,0.14) | 64.72(55.81,74.64) | 0.21(0.18,0.25) | 1.49(0.95,2.03) |
| Central Latin America | 6.60(5.91,7.31) | 0.07(0.06,0.08) | 44.54(38.30,50.77) | 0.15(0.13,0.17) | 1.89(1.19,2.61) |
| Central Sub-Saharan Africa | 0.57(0.26,1.17) | 0.02(0.01,0.05) | 2.06(1.06,3.88) | 0.04(0.02,0.07) | 1.39(0.97,1.81) |
| East Asia | 50.95(35.30,74.90) | 0.05(0.04,0.08) | 532.57(367.41,670.62) | 0.20(0.14,0.25) | 4.86(4.55,5.18) |
| Eastern Europe | 57.39(38.96,76.98) | 0.16(0.11,0.21) | 100.45(85.84,115.61) | 0.21(0.18,0.24) | -0.19(-0.75,0.38) |
| Eastern Sub-Saharan Africa | 1.41(0.70,2.90) | 0.02(0.01,0.04) | 7.02(4.23,11.26) | 0.04(0.02,0.06) | 2.76(2.56,2.97) |
| High-income Asia Pacific | 21.21(18.92,23.51) | 0.09(0.08,0.09) | 82.30(69.94,92.48) | 0.13(0.11,0.15) | 0.87(0.36,1.39) |
| High-income North America | 69.42(61.82,75.80) | 0.15(0.13,0.16) | 139.00(121.37,153.34) | 0.16(0.14,0.17) | 0.01(-0.34,0.37) |
| North Africa and Middle East | 6.98(3.99,11.73) | 0.04(0.02,0.07) | 52.10(38.58,70.39) | 0.11(0.08,0.15) | 3.52(3.28,3.76) |
| Oceania | 0.08(0.04,0.16) | 0.03(0.01,0.06) | 0.20(0.11,0.40) | 0.03(0.01,0.05) | -0.42(-0.68,-0.15) |
| South Asia | 13.56(8.52,20.20) | 0.02(0.01,0.03) | 92.00(75.72,113.26) | 0.05(0.04,0.07) | 2.50(2.23,2.77) |
| Southeast Asia | 14.17(9.91,19.15) | 0.05(0.04,0.07) | 93.07(73.47,116.19) | 0.12(0.10,0.15) | 2.76(2.64,2.88) |
| Southern Latin America | 6.67(4.91,8.69) | 0.12(0.08,0.15) | 20.15(15.16,25.98) | 0.18(0.13,0.23) | 1.83(1.46,2.20) |
| Southern Sub-Saharan Africa | 2.59(1.43,3.63) | 0.09(0.05,0.12) | 10.36(7.01,12.55) | 0.16(0.11,0.20) | 1.94(1.70,2.18) |
| Tropical Latin America | 8.70(7.62,9.76) | 0.08(0.07,0.09) | 52.62(45.24,60.14) | 0.17(0.14,0.19) | 1.92(1.43,2.41) |
| Western Europe | 124.54(112.72,135.39) | 0.16(0.15,0.18) | 234.88(198.62,270.43) | 0.18(0.16,0.21) | 0.36(0.04,0.68) |
| Western Sub-Saharan Africa | 2.85(1.58,4.84) | 0.03(0.02,0.05) | 13.64(9.29,17.97) | 0.07(0.05,0.09) | 3.06(2.86,3.26) |

EAPC: Estimated annual percentage change
