# Supplementary material for: Global, regional, and national burden of neuroblastoma and peripheral nervous system tumours in individuals aged over 60 from 1990 to 2021: a trend analysis of global burden of disease study
Source: J Health Popul Nutr. 2025 Mar 17;44:78. doi: 10.1186/s41043-025-00810-9 (PMC11916991; doi:10.1186/s41043-025-00810-9)
Supplement: Supplementary file 10 — Supplementary Material 10 [file 41043_2025_810_MOESM10_ESM.docx]

Supplement 3. The age-standardized DALYs rate, number of DALYs, and EAPC of neuroblastoma and peripheral nervous system tumours among elderly individuals aged 60 and above globally and regionally from 1990 to 2021.

|  | DALYs (95% UI) | | | | |
| --- | --- | --- | --- | --- | --- |
|  | Cases in 1990 (million) | Age-standardised rate in 1990 (per 100 000) | Cases in 2021(million) | Age-standardised rate in 2021 (per 100 000) | EAPC (95% CI) |
|  |  |  |  |  |  |
| Global | 8840.78(7492.67,10292.56) | 1.82(1.55,2.12) | 32050.19(26840.23,35736.83) | 2.95(2.47,3.29) | 1.40(1.27,1.53) |
| Sex |  | | | | |
| Male | 4645.69(3896.80,5337.35) | 2.13(1.79,2.44) | 18524.90(15972.33,21058.85) | 3.69(3.18,4.19) | 1.62(1.49,1.75) |
| Female | 4195.09(3488.46,5171.87) | 1.58(1.31,1.94) | 13525.29(10147.17,15765.45) | 2.32(1.74,2.71) | 1.11(0.97,1.25) |
| SDI quintile |  | | | | |
| High | 4087.65(3757.96,4368.17) | 2.85(2.62,3.04) | 9109.09(8104.04,9993.24) | 3.37(3.01,3.69) | 0.30(-0.05,0.64) |
| High middle | 3016.73(2330.68,3793.18) | 2.36(1.83,2.96) | 10793.04(8504.66,12652.38) | 4.21(3.32,4.93) | 1.74(1.62,1.86) |
| Middle | 1259.66(963.15,1632.15) | 1.04(0.79,1.35) | 9605.90(7714.75,11177.91) | 2.89(2.32,3.37) | 3.42(3.32,3.53) |
| Low middle | 379.98(261.92,560.78) | 0.54(0.37,0.79) | 2145.51(1798.19,2560.22) | 1.24(1.04,1.48) | 2.61(2.52,2.70) |
| Low | 84.56(42.43,165.91) | 0.32(0.16,0.62) | 363.76(230.11,559.66) | 0.63(0.40,0.98) | 2.11(1.77,2.45) |
| GBD region |  | | | | |
| Andean Latin America | 39.73(29.33,54.52) | 1.67(1.23,2.29) | 259.21(189.44,352.27) | 3.61(2.64,4.91) | 2.70(2.51,2.89) |
| Australasia | 107.50(86.77,130.74) | 3.46(2.79,4.21) | 241.71(182.69,312.59) | 3.47(2.63,4.48) | -0.27(-0.58,0.03) |
| Caribbean | 47.77(38.68,58.74) | 1.50(1.21,1.84) | 172.32(139.20,209.76) | 2.59(2.09,3.16) | 2.34(1.92,2.75) |
| Central Asia | 87.23(51.81,125.53) | 1.51(0.88,2.18) | 405.55(319.95,493.95) | 4.06(3.21,4.93) | 3.87(3.62,4.12) |
| Central Europe | 493.78(419.51,571.11) | 2.51(2.13,2.90) | 1311.03(1135.37,1507.81) | 4.40(3.82,5.06) | 1.48(0.95,2.02) |
| Central Latin America | 138.12(124.06,152.76) | 1.44(1.29,1.59) | 925.90(798.23,1056.54) | 3.00(2.59,3.42) | 1.91(1.19,2.63) |
| Central Sub-Saharan Africa | 13.17(6.11,27.14) | 0.51(0.23,1.05) | 47.02(24.38,88.36) | 0.79(0.41,1.49) | 1.37(0.96,1.78) |
| East Asia | 1113.35(779.11,1628.93) | 1.07(0.74,1.57) | 10742.81(7395.77,13606.59) | 3.85(2.66,4.87) | 4.82(4.49,5.15) |
| Eastern Europe | 1323.25(886.71,1780.75) | 3.49(2.35,4.69) | 2230.31(1901.69,2576.02) | 4.58(3.91,5.29) | -0.30(-0.88,0.29) |
| Eastern Sub-Saharan Africa | 31.76(15.66,65.91) | 0.36(0.18,0.75) | 154.40(92.28,247.54) | 0.82(0.49,1.32) | 2.68(2.48,2.88) |
| High-income Asia Pacific | 444.56(398.52,492.48) | 1.74(1.56,1.93) | 1503.51(1304.42,1681.92) | 2.66(2.33,2.97) | 0.82(0.29,1.35) |
| High-income North America | 1371.14(1232.09,1491.68) | 2.98(2.68,3.24) | 2711.25(2405.35,2976.64) | 3.10(2.75,3.40) | -0.04(-0.39,0.30) |
| North Africa and Middle East | 151.29(86.60,254.04) | 0.78(0.44,1.31) | 1093.83(811.20,1472.11) | 2.11(1.56,2.85) | 3.42(3.20,3.64) |
| Oceania | 1.66(0.82,3.62) | 0.54(0.26,1.13) | 4.21(2.26,8.71) | 0.53(0.29,1.09) | -0.49(-0.80,-0.19) |
| South Asia | 303.91(190.71,454.31) | 0.46(0.29,0.68) | 1963.98(1618.15,2413.37) | 1.09(0.90,1.34) | 2.40(2.12,2.68) |
| Southeast Asia | 313.69(220.62,422.68) | 1.06(0.74,1.43) | 2037.16(1614.88,2531.66) | 2.53(2.00,3.16) | 2.73(2.61,2.85) |
| Southern Latin America | 139.58(102.19,182.17) | 2.35(1.72,3.07) | 403.39(303.34,520.97) | 3.62(2.72,4.68) | 1.78(1.41,2.15) |
| Southern Sub-Saharan Africa | 53.81(30.38,74.94) | 1.70(0.95,2.38) | 219.68(152.29,264.96) | 3.22(2.19,3.89) | 1.95(1.69,2.20) |
| Tropical Latin America | 189.24(166.10,212.54) | 1.73(1.51,1.94) | 1105.45(958.80,1258.05) | 3.42(2.97,3.90) | 1.86(1.36,2.37) |
| Western Europe | 2417.10(2202.77,2620.57) | 3.19(2.91,3.46) | 4238.78(3632.92,4867.86) | 3.61(3.11,4.13) | 0.31(-0.01,0.63) |
| Western Sub-Saharan Africa | 59.14(32.09,101.70) | 0.59(0.32,1.00) | 278.69(182.91,371.67) | 1.34(0.90,1.78) | 2.95(2.76,3.15) |

DALYs: Disability-Adjusted Life Years; EAPC: Estimated Annual Percentage Change
