# Supplementary material for: Global, regional, and national burden of neuroblastoma and peripheral nervous system tumours in individuals aged over 60 from 1990 to 2021: a trend analysis of global burden of disease study
Source: J Health Popul Nutr. 2025 Mar 17;44:78. doi: 10.1186/s41043-025-00810-9 (PMC11916991; doi:10.1186/s41043-025-00810-9)
Supplement: Supplementary file 11 — Supplementary Material 11 [file 41043_2025_810_MOESM11_ESM.docx]

Supplement 4. The age-standardized prevalence rate, number of cases, and EAPC of neuroblastoma and peripheral nervous system tumours among individuals aged 60 and above across 204 countries from 1990 to 2021

| Nation | Prevalence (95% UI) | | | | |
| --- | --- | --- | --- | --- | --- |
|  | Cases in 1990 (million) | Age-standardised rate in 1990 (per 100 000) | Cases in 2021(million) | Age-standardised rate in 2021 (per 100 000) | EAPC (95% CI) |
|  |  |  |  |  |  |
| American Samoa | 0.00(0.00,0.00) | 0.05(0.02,0.09) | 0.00(0.00,0.01) | 0.04(0.02,0.08) | -1.18(-2.21,-0.15) |
| Antigua and Barbuda | 0.00(0.00,0.00) | 0.03(0.02,0.05) | 0.01(0.00,0.01) | 0.06(0.04,0.09) | 2.00(1.75,2.24) |
| Arab Republic of Egypt | 0.46(0.19,0.96) | 0.02(0.01,0.03) | 2.50(1.37,4.92) | 0.04(0.02,0.07) | 2.39(1.95,2.82) |
| Argentine Republic | 3.28(2.18,4.77) | 0.08(0.05,0.12) | 9.44(6.44,13.22) | 0.13(0.09,0.18) | 2.17(1.94,2.40) |
| Australia | 3.46(2.58,4.46) | 0.14(0.10,0.17) | 8.40(5.92,11.45) | 0.14(0.10,0.19) | -0.24(-0.49,0.01) |
| Barbados | 0.05(0.04,0.07) | 0.14(0.10,0.19) | 0.16(0.11,0.22) | 0.24(0.17,0.32) | 2.37(2.08,2.66) |
| Belize | 0.00(0.00,0.00) | 0.03(0.02,0.04) | 0.03(0.02,0.03) | 0.07(0.05,0.10) | 3.19(2.25,4.15) |
| Bermuda | 0.00(0.00,0.00) | 0.04(0.03,0.05) | 0.01(0.01,0.01) | 0.06(0.04,0.08) | 1.42(0.99,1.85) |
| Bolivarian Republic of Venezuela | 0.80(0.58,1.10) | 0.07(0.05,0.10) | 4.23(2.79,6.13) | 0.11(0.08,0.17) | 1.16(0.63,1.69) |
| Bosnia and Herzegovina | 0.21(0.11,0.40) | 0.05(0.02,0.09) | 0.84(0.48,1.35) | 0.10(0.06,0.16) | 3.27(2.86,3.69) |
| Brunei Darussalam | 0.01(0.00,0.01) | 0.06(0.03,0.12) | 0.03(0.02,0.04) | 0.07(0.04,0.11) | 0.21(-0.13,0.55) |
| Burkina Faso | 0.01(0.00,0.02) | 0.00(0.00,0.00) | 0.03(0.02,0.07) | 0.00(0.00,0.01) | 1.49(1.33,1.65) |
| Canada | 5.72(4.24,7.63) | 0.14(0.10,0.18) | 12.11(8.52,16.40) | 0.12(0.09,0.17) | -0.33(-0.57,-0.08) |
| Central African Republic | 0.01(0.00,0.03) | 0.01(0.00,0.02) | 0.03(0.01,0.06) | 0.01(0.00,0.03) | 0.76(0.67,0.85) |
| Commonwealth of Dominica | 0.00(0.00,0.00) | 0.03(0.02,0.06) | 0.01(0.00,0.01) | 0.07(0.04,0.13) | 2.72(2.32,3.12) |
| Commonwealth of the Bahamas | 0.01(0.01,0.01) | 0.05(0.03,0.06) | 0.03(0.02,0.05) | 0.07(0.05,0.09) | 1.49(1.32,1.66) |
| Cook Islands | 0.00(0.00,0.00) | 0.02(0.01,0.03) | 0.00(0.00,0.00) | 0.03(0.02,0.06) | 2.15(2.00,2.31) |
| Czech Republic | 2.33(1.60,3.25) | 0.13(0.09,0.18) | 6.75(4.46,9.68) | 0.24(0.16,0.34) | 2.03(1.70,2.37) |
| Democratic People's Republic of Korea | 0.47(0.24,0.87) | 0.03(0.01,0.05) | 1.65(0.78,3.11) | 0.04(0.02,0.08) | 2.15(1.83,2.48) |
| Democratic Republic of Sao Tome and Principe | 0.00(0.00,0.00) | 0.00(0.00,0.01) | 0.00(0.00,0.00) | 0.01(0.01,0.02) | 3.29(3.05,3.53) |
| Democratic Republic of the Congo | 0.19(0.07,0.44) | 0.01(0.00,0.03) | 0.53(0.20,1.25) | 0.01(0.01,0.03) | 0.76(0.17,1.35) |
| Democratic Republic of Timor-Leste | 0.00(0.00,0.00) | 0.01(0.00,0.02) | 0.02(0.01,0.05) | 0.02(0.01,0.04) | 3.90(3.72,4.09) |
| Democratic Socialist Republic of Sri Lanka | 0.69(0.35,1.21) | 0.06(0.03,0.10) | 4.47(2.30,7.66) | 0.13(0.07,0.22) | 2.56(2.32,2.80) |
| Dominican Republic | 0.03(0.01,0.07) | 0.01(0.00,0.02) | 0.53(0.30,0.88) | 0.04(0.03,0.07) | 5.92(5.01,6.84) |
| Eastern Republic of Uruguay | 0.49(0.32,0.74) | 0.09(0.06,0.14) | 1.21(0.79,1.77) | 0.17(0.11,0.25) | 2.05(1.79,2.31) |
| Federal Democratic Republic of Ethiopia | 0.20(0.06,0.65) | 0.01(0.00,0.03) | 1.08(0.41,2.49) | 0.02(0.01,0.05) | 2.97(2.61,3.33) |
| Federal Democratic Republic of Nepal | 0.05(0.01,0.12) | 0.00(0.00,0.01) | 0.38(0.17,0.72) | 0.01(0.01,0.03) | 3.78(3.55,4.02) |
| Federal Republic of Germany | 20.02(14.61,26.59) | 0.12(0.09,0.16) | 42.94(30.39,57.70) | 0.17(0.12,0.22) | 0.72(0.20,1.24) |
| Federal Republic of Nigeria | 1.29(0.65,2.37) | 0.03(0.01,0.05) | 7.28(4.43,10.63) | 0.08(0.05,0.12) | 4.09(3.85,4.33) |
| Federal Republic of Somalia | 0.01(0.00,0.02) | 0.00(0.00,0.01) | 0.03(0.01,0.11) | 0.00(0.00,0.02) | 0.72(0.67,0.77) |
| Federated States of Micronesia | 0.00(0.00,0.00) | 0.01(0.00,0.02) | 0.00(0.00,0.00) | 0.01(0.01,0.03) | 0.54(0.42,0.66) |
| Federative Republic of Brazil | 4.78(3.67,6.38) | 0.05(0.04,0.06) | 32.82(24.81,42.39) | 0.11(0.08,0.14) | 2.27(1.69,2.85) |
| French Republic | 10.98(8.05,14.62) | 0.10(0.07,0.13) | 24.48(16.34,34.53) | 0.13(0.09,0.19) | 0.85(0.47,1.24) |
| Gabonese Republic | 0.02(0.01,0.04) | 0.03(0.01,0.06) | 0.07(0.04,0.13) | 0.06(0.03,0.11) | 2.60(2.50,2.70) |
| Georgia | 0.10(0.06,0.16) | 0.01(0.01,0.02) | 1.98(1.20,2.99) | 0.25(0.15,0.38) | 13.35(11.93,14.80) |
| Grand Duchy of Luxembourg | 0.07(0.05,0.09) | 0.10(0.08,0.12) | 0.15(0.12,0.19) | 0.11(0.08,0.14) | 0.54(0.08,1.01) |
| Greenland | 0.00(0.00,0.00) | 0.06(0.03,0.10) | 0.01(0.00,0.01) | 0.08(0.02,0.15) | 1.26(1.06,1.46) |
| Grenada | 0.00(0.00,0.00) | 0.03(0.01,0.04) | 0.01(0.01,0.02) | 0.08(0.05,0.12) | 3.92(3.53,4.30) |
| Guam | 0.00(0.00,0.00) | 0.02(0.01,0.03) | 0.01(0.00,0.01) | 0.02(0.02,0.03) | 0.97(-0.24,2.19) |
| Hashemite Kingdom of Jordan | 0.06(0.03,0.10) | 0.04(0.02,0.08) | 0.59(0.32,1.00) | 0.08(0.04,0.13) | 2.24(1.98,2.49) |
| Hellenic Republic | 1.16(0.93,1.43) | 0.06(0.05,0.07) | 2.48(1.97,3.06) | 0.08(0.06,0.10) | 0.90(0.62,1.18) |
| Hungary | 1.87(1.27,2.62) | 0.10(0.06,0.13) | 6.68(4.40,9.81) | 0.26(0.17,0.38) | 2.30(1.68,2.93) |
| Independent State of Papua New Guinea | 0.01(0.00,0.03) | 0.00(0.00,0.01) | 0.03(0.01,0.10) | 0.01(0.00,0.02) | 0.92(0.78,1.06) |
| Independent State of Samoa | 0.00(0.00,0.01) | 0.03(0.02,0.13) | 0.01(0.00,0.04) | 0.06(0.02,0.22) | 1.70(1.55,1.85) |
| Ireland | 0.69(0.51,0.92) | 0.13(0.09,0.17) | 1.31(0.92,1.80) | 0.13(0.09,0.17) | -0.44(-0.98,0.12) |
| Islamic Republic of Afghanistan | 0.01(0.00,0.07) | 0.00(0.00,0.01) | 0.04(0.02,0.16) | 0.01(0.00,0.02) | 5.12(4.81,5.42) |
| Islamic Republic of Iran | 0.21(0.05,0.44) | 0.01(0.00,0.02) | 3.25(0.55,4.90) | 0.04(0.01,0.06) | 6.09(5.56,6.63) |
| Islamic Republic of Mauritania | 0.01(0.00,0.01) | 0.01(0.00,0.01) | 0.03(0.01,0.04) | 0.01(0.01,0.02) | 1.76(1.46,2.06) |
| Islamic Republic of Pakistan | 0.94(0.53,1.59) | 0.01(0.01,0.02) | 4.96(2.92,8.04) | 0.04(0.02,0.06) | 2.94(2.81,3.06) |
| Jamaica | 0.10(0.07,0.14) | 0.04(0.03,0.06) | 0.51(0.32,0.78) | 0.13(0.08,0.20) | 3.37(2.61,4.13) |
| Japan | 12.93(10.79,15.26) | 0.06(0.05,0.07) | 53.98(43.65,63.94) | 0.12(0.10,0.14) | 1.47(0.83,2.11) |
| Kingdom of Bahrain | 0.01(0.00,0.01) | 0.03(0.02,0.06) | 0.10(0.05,0.18) | 0.13(0.06,0.22) | 5.68(4.92,6.43) |
| Kingdom of Belgium | 1.98(1.40,2.72) | 0.10(0.07,0.13) | 4.07(2.84,5.58) | 0.13(0.09,0.18) | 0.87(0.32,1.43) |
| Kingdom of Bhutan | 0.00(0.00,0.00) | 0.00(0.00,0.01) | 0.01(0.01,0.03) | 0.02(0.01,0.04) | 4.78(4.63,4.94) |
| Kingdom of Cambodia | 0.06(0.02,0.14) | 0.01(0.00,0.03) | 0.45(0.22,0.86) | 0.03(0.02,0.06) | 3.65(3.47,3.83) |
| Kingdom of Denmark | 0.83(0.59,1.11) | 0.08(0.06,0.10) | 3.27(2.28,4.47) | 0.21(0.15,0.29) | 2.66(2.14,3.19) |
| Kingdom of Eswatini | 0.01(0.00,0.02) | 0.03(0.01,0.06) | 0.04(0.02,0.08) | 0.07(0.04,0.13) | 3.27(2.97,3.58) |
| Kingdom of Lesotho | 0.02(0.01,0.04) | 0.02(0.01,0.04) | 0.06(0.03,0.10) | 0.05(0.03,0.08) | 3.75(3.49,4.02) |
| Kingdom of Morocco | 0.37(0.17,0.74) | 0.02(0.01,0.04) | 2.82(1.48,5.02) | 0.07(0.04,0.12) | 4.01(3.74,4.29) |
| Kingdom of Norway | 1.17(0.94,1.43) | 0.13(0.10,0.16) | 2.20(1.75,2.70) | 0.17(0.13,0.20) | -0.29(-0.86,0.29) |
| Kingdom of Saudi Arabia | 0.13(0.04,0.25) | 0.02(0.01,0.04) | 1.10(0.48,1.83) | 0.06(0.03,0.11) | 3.85(2.84,4.87) |
| Kingdom of Spain | 7.92(5.73,10.55) | 0.11(0.08,0.15) | 18.30(12.59,25.63) | 0.14(0.10,0.20) | 0.69(0.28,1.11) |
| Kingdom of Sweden | 1.78(1.29,2.39) | 0.09(0.07,0.12) | 3.97(2.73,5.53) | 0.14(0.10,0.19) | 1.12(-0.04,2.29) |
| Kingdom of Thailand | 1.83(1.04,3.01) | 0.05(0.03,0.08) | 14.62(8.58,24.09) | 0.10(0.06,0.17) | 2.41(2.26,2.56) |
| Kingdom of the Netherlands | 5.05(3.73,6.68) | 0.19(0.14,0.26) | 9.33(6.61,12.75) | 0.20(0.14,0.27) | -0.32(-0.70,0.06) |
| Kingdom of Tonga | 0.00(0.00,0.00) | 0.01(0.00,0.03) | 0.00(0.00,0.00) | 0.02(0.01,0.03) | 1.34(1.07,1.61) |
| Kyrgyz Republic | 0.04(0.03,0.06) | 0.01(0.01,0.02) | 0.44(0.28,0.64) | 0.08(0.05,0.12) | 8.59(7.37,9.84) |
| Lao People's Democratic Republic | 0.02(0.01,0.05) | 0.01(0.00,0.02) | 0.14(0.07,0.28) | 0.03(0.01,0.05) | 3.81(3.72,3.90) |
| Lebanese Republic | 0.07(0.04,0.13) | 0.03(0.01,0.05) | 0.46(0.25,0.76) | 0.06(0.03,0.10) | 3.53(3.19,3.88) |
| Malaysia | 0.62(0.32,1.15) | 0.06(0.03,0.11) | 5.14(3.02,8.17) | 0.15(0.09,0.24) | 2.69(2.29,3.09) |
| Mongolia | 0.05(0.02,0.09) | 0.04(0.02,0.08) | 0.29(0.16,0.47) | 0.12(0.07,0.19) | 3.68(3.45,3.91) |
| Montenegro | 0.05(0.03,0.07) | 0.06(0.04,0.09) | 0.17(0.11,0.23) | 0.13(0.09,0.18) | 2.64(2.32,2.97) |
| New Zealand | 0.73(0.54,0.99) | 0.14(0.10,0.19) | 1.84(1.34,2.46) | 0.16(0.12,0.22) | 0.35(-0.35,1.06) |
| North Macedonia | 0.09(0.06,0.13) | 0.04(0.03,0.06) | 0.42(0.28,0.59) | 0.10(0.07,0.14) | 3.06(2.52,3.60) |
| Northern Mariana Islands | 0.00(0.00,0.00) | 0.01(0.00,0.01) | 0.00(0.00,0.00) | 0.01(0.00,0.01) | 1.28(0.32,2.24) |
| Palestine | 0.03(0.01,0.07) | 0.03(0.01,0.07) | 0.24(0.13,0.38) | 0.09(0.05,0.14) | 3.23(3.04,3.42) |
| People's Democratic Republic of Algeria | 0.22(0.11,0.42) | 0.02(0.01,0.03) | 1.57(0.85,2.83) | 0.04(0.02,0.07) | 2.42(2.22,2.62) |
| People's Republic of Bangladesh | 0.35(0.12,0.81) | 0.01(0.00,0.02) | 3.25(1.40,6.21) | 0.02(0.01,0.04) | 3.40(3.09,3.72) |
| People's Republic of China | 28.02(17.52,44.04) | 0.03(0.02,0.05) | 389.19(253.48,511.35) | 0.15(0.10,0.19) | 6.06(5.68,6.43) |
| Plurinational State of Bolivia | 0.14(0.08,0.25) | 0.04(0.02,0.07) | 1.05(0.56,1.83) | 0.10(0.05,0.17) | 3.10(2.96,3.23) |
| Portuguese Republic | 1.84(1.32,2.47) | 0.10(0.07,0.14) | 3.88(2.66,5.43) | 0.12(0.08,0.16) | 0.55(0.23,0.88) |
| Principality of Andorra | 0.01(0.00,0.01) | 0.11(0.06,0.20) | 0.02(0.01,0.04) | 0.12(0.06,0.21) | 0.69(0.46,0.93) |
| Principality of Monaco | 0.00(0.00,0.00) | 0.00(0.00,0.00) | 0.00(0.00,0.00) | 0.00(0.00,0.00) | 0.61(0.55,0.68) |
| Puerto Rico | 0.22(0.14,0.33) | 0.05(0.03,0.07) | 0.88(0.58,1.28) | 0.09(0.06,0.14) | 2.08(1.58,2.58) |
| Republic of Albania | 0.03(0.02,0.05) | 0.01(0.01,0.02) | 0.15(0.09,0.26) | 0.03(0.01,0.04) | 3.06(2.74,3.38) |
| Republic of Angola | 0.04(0.01,0.10) | 0.01(0.00,0.02) | 0.31(0.12,0.67) | 0.03(0.01,0.06) | 2.78(2.51,3.04) |
| Republic of Armenia | 0.19(0.10,0.34) | 0.06(0.03,0.10) | 1.63(1.03,2.47) | 0.28(0.17,0.42) | 6.47(5.66,7.29) |
| Republic of Austria | 1.75(1.30,2.30) | 0.11(0.08,0.15) | 2.78(1.96,3.77) | 0.12(0.08,0.16) | 0.23(-0.26,0.71) |
| Republic of Azerbaijan | 0.29(0.14,0.56) | 0.05(0.02,0.10) | 0.93(0.51,1.61) | 0.08(0.04,0.14) | 2.26(1.80,2.73) |
| Republic of Belarus | 1.18(0.76,1.78) | 0.07(0.05,0.11) | 6.54(4.12,9.81) | 0.30(0.19,0.45) | 4.05(3.53,4.57) |
| Republic of Benin | 0.01(0.00,0.01) | 0.00(0.00,0.01) | 0.03(0.01,0.05) | 0.01(0.00,0.01) | 1.39(1.20,1.59) |
| Republic of Botswana | 0.02(0.01,0.04) | 0.03(0.01,0.06) | 0.12(0.06,0.22) | 0.08(0.04,0.14) | 3.27(2.97,3.57) |
| Republic of Bulgaria | 0.59(0.36,0.89) | 0.04(0.02,0.06) | 1.82(1.18,2.73) | 0.09(0.06,0.14) | 2.23(1.56,2.91) |
| Republic of Burundi | 0.02(0.01,0.04) | 0.01(0.00,0.02) | 0.05(0.02,0.12) | 0.01(0.00,0.02) | 0.72(0.52,0.91) |
| Republic of Cabo Verde | 0.00(0.00,0.00) | 0.00(0.00,0.00) | 0.00(0.00,0.00) | 0.00(0.00,0.01) | 5.13(4.94,5.33) |
| Republic of Cameroon | 0.02(0.01,0.04) | 0.01(0.00,0.01) | 0.11(0.06,0.19) | 0.01(0.00,0.02) | 1.47(1.25,1.69) |
| Republic of Chad | 0.01(0.00,0.01) | 0.00(0.00,0.00) | 0.02(0.01,0.03) | 0.00(0.00,0.01) | 1.78(1.68,1.88) |
| Republic of Chile | 0.33(0.23,0.46) | 0.03(0.02,0.04) | 3.50(2.40,4.86) | 0.11(0.07,0.15) | 4.80(3.51,6.11) |
| Republic of Colombia | 0.82(0.56,1.19) | 0.04(0.03,0.06) | 7.36(4.86,10.64) | 0.11(0.07,0.16) | 2.20(1.47,2.93) |
| Republic of Costa Rica | 0.11(0.07,0.15) | 0.05(0.03,0.07) | 0.93(0.62,1.31) | 0.14(0.09,0.19) | 2.43(1.82,3.04) |
| Republic of Croatia | 1.19(0.82,1.63) | 0.16(0.11,0.21) | 4.55(3.06,6.39) | 0.37(0.25,0.52) | 2.70(2.37,3.04) |
| Republic of Cuba | 0.79(0.54,1.13) | 0.06(0.04,0.09) | 2.59(1.80,3.66) | 0.11(0.07,0.15) | 2.68(2.10,3.27) |
| Republic of Cyprus | 0.14(0.07,0.25) | 0.15(0.07,0.28) | 0.53(0.32,0.83) | 0.20(0.12,0.31) | 0.85(0.57,1.13) |
| The Republic of Côte d'Ivoire | 0.01(0.01,0.02) | 0.00(0.00,0.01) | 0.05(0.03,0.10) | 0.00(0.00,0.01) | 1.52(1.32,1.72) |
| Republic of Djibouti | 0.00(0.00,0.00) | 0.01(0.01,0.02) | 0.02(0.01,0.04) | 0.03(0.02,0.06) | 2.96(2.83,3.09) |
| Republic of Ecuador | 0.25(0.16,0.37) | 0.04(0.03,0.06) | 2.77(1.74,4.21) | 0.14(0.09,0.21) | 4.61(3.92,5.30) |
| Republic of El Salvador | 0.06(0.04,0.10) | 0.02(0.01,0.03) | 0.31(0.20,0.47) | 0.04(0.03,0.06) | 2.51(2.18,2.83) |
| Republic of Equatorial Guinea | 0.00(0.00,0.01) | 0.01(0.00,0.03) | 0.04(0.02,0.06) | 0.07(0.03,0.13) | 6.64(6.48,6.79) |
| Republic of Estonia | 0.23(0.15,0.35) | 0.09(0.06,0.13) | 1.49(0.98,2.20) | 0.42(0.28,0.63) | 3.57(2.87,4.28) |
| Republic of Fiji | 0.02(0.01,0.04) | 0.06(0.02,0.12) | 0.04(0.02,0.11) | 0.05(0.02,0.13) | -1.45(-2.05,-0.85) |
| Republic of Finland | 0.74(0.52,1.01) | 0.08(0.05,0.11) | 3.64(2.45,5.12) | 0.21(0.15,0.30) | 3.21(2.66,3.77) |
| Republic of Ghana | 0.03(0.01,0.06) | 0.01(0.00,0.01) | 0.07(0.03,0.12) | 0.00(0.00,0.01) | -2.11(-3.26,-0.94) |
| Republic of Guatemala | 0.04(0.03,0.06) | 0.01(0.01,0.02) | 0.23(0.16,0.31) | 0.02(0.01,0.02) | 0.71(0.38,1.03) |
| Republic of Guinea | 0.01(0.01,0.03) | 0.00(0.00,0.01) | 0.04(0.02,0.08) | 0.01(0.00,0.01) | 2.05(1.98,2.13) |
| Republic of Guinea-Bissau | 0.00(0.00,0.00) | 0.00(0.00,0.01) | 0.00(0.00,0.01) | 0.01(0.00,0.01) | 1.16(1.06,1.27) |
| Republic of Guyana | 0.00(0.00,0.00) | 0.00(0.00,0.00) | 0.03(0.02,0.05) | 0.04(0.02,0.06) | 8.01(5.68,10.38) |
| Republic of Haiti | 0.04(0.02,0.09) | 0.01(0.00,0.03) | 0.16(0.07,0.33) | 0.02(0.01,0.04) | 2.12(1.93,2.32) |
| Republic of Honduras | 0.05(0.02,0.09) | 0.02(0.01,0.04) | 0.56(0.31,0.99) | 0.08(0.04,0.13) | 4.81(4.43,5.20) |
| Republic of Iceland | 0.05(0.03,0.06) | 0.13(0.09,0.17) | 0.14(0.10,0.19) | 0.18(0.13,0.25) | 1.42(1.18,1.66) |
| Republic of India | 5.63(3.26,9.16) | 0.01(0.01,0.02) | 45.69(33.45,62.67) | 0.03(0.02,0.04) | 3.14(2.81,3.47) |
| Republic of Indonesia | 1.94(1.18,3.08) | 0.02(0.01,0.03) | 17.36(11.51,25.10) | 0.06(0.04,0.09) | 3.94(3.80,4.08) |
| Republic of Iraq | 0.11(0.05,0.22) | 0.01(0.01,0.02) | 1.19(0.64,2.00) | 0.05(0.03,0.08) | 4.27(4.02,4.53) |
| Republic of Italy | 11.79(9.60,13.99) | 0.10(0.08,0.12) | 27.95(21.88,34.08) | 0.15(0.12,0.18) | 1.41(1.06,1.76) |
| Republic of Kazakhstan | 0.58(0.32,0.94) | 0.04(0.02,0.06) | 1.83(1.07,2.89) | 0.09(0.05,0.13) | 2.37(2.00,2.75) |
| Republic of Kenya | 0.05(0.02,0.11) | 0.01(0.00,0.01) | 0.47(0.27,0.75) | 0.02(0.01,0.03) | 3.93(3.80,4.07) |
| Republic of Kiribati | 0.00(0.00,0.00) | 0.00(0.00,0.00) | 0.00(0.00,0.00) | 0.00(0.00,0.01) | 1.27(1.19,1.34) |
| Republic of Korea | 2.21(1.28,3.62) | 0.07(0.04,0.11) | 9.74(5.85,15.05) | 0.08(0.05,0.12) | -0.02(-0.40,0.35) |
| Republic of Latvia | 0.29(0.18,0.44) | 0.06(0.04,0.10) | 0.87(0.54,1.34) | 0.17(0.10,0.26) | 3.49(3.19,3.80) |
| Republic of Liberia | 0.00(0.00,0.01) | 0.00(0.00,0.01) | 0.01(0.00,0.02) | 0.00(0.00,0.01) | 1.23(0.90,1.56) |
| Republic of Lithuania | 0.38(0.24,0.55) | 0.06(0.04,0.09) | 2.05(1.29,3.07) | 0.27(0.17,0.41) | 4.95(4.69,5.22) |
| Republic of Madagascar | 0.05(0.02,0.10) | 0.01(0.00,0.02) | 0.16(0.08,0.30) | 0.01(0.01,0.03) | 1.57(1.25,1.90) |
| Republic of Malawi | 0.08(0.04,0.15) | 0.02(0.01,0.03) | 0.32(0.18,0.54) | 0.04(0.02,0.07) | 2.58(2.44,2.72) |
| Republic of Maldives | 0.01(0.00,0.01) | 0.06(0.02,0.15) | 0.07(0.04,0.12) | 0.21(0.12,0.35) | 3.76(3.26,4.26) |
| Republic of Mali | 0.01(0.00,0.02) | 0.00(0.00,0.00) | 0.03(0.02,0.06) | 0.00(0.00,0.01) | 1.95(1.83,2.06) |
| Republic of Malta | 0.11(0.08,0.15) | 0.20(0.15,0.27) | 0.34(0.24,0.46) | 0.25(0.18,0.35) | 0.67(0.15,1.20) |
| Republic of Mauritius | 0.04(0.03,0.05) | 0.04(0.03,0.05) | 0.28(0.21,0.37) | 0.11(0.08,0.15) | 3.26(2.28,4.24) |
| Republic of Moldova | 0.21(0.14,0.32) | 0.04(0.03,0.06) | 1.02(0.78,1.33) | 0.13(0.10,0.16) | 4.66(4.22,5.11) |
| Republic of Mozambique | 0.04(0.01,0.10) | 0.01(0.00,0.02) | 0.18(0.08,0.39) | 0.02(0.01,0.03) | 3.24(3.05,3.43) |
| Republic of Namibia | 0.02(0.01,0.03) | 0.02(0.01,0.04) | 0.07(0.03,0.14) | 0.04(0.02,0.09) | 2.47(2.30,2.65) |
| Republic of Nauru | 0.00(0.00,0.00) | 0.02(0.01,0.04) | 0.00(0.00,0.00) | 0.02(0.01,0.04) | 0.54(0.18,0.90) |
| Republic of Nicaragua | 0.07(0.04,0.13) | 0.04(0.02,0.07) | 0.51(0.33,0.74) | 0.09(0.06,0.13) | 2.45(1.99,2.92) |
| Republic of Niue | 0.00(0.00,0.00) | 0.02(0.01,0.04) | 0.00(0.00,0.00) | 0.03(0.01,0.05) | 1.20(0.91,1.50) |
| Republic of Palau | 0.00(0.00,0.00) | 0.01(0.00,0.01) | 0.00(0.00,0.00) | 0.01(0.00,0.02) | 0.14(-0.02,0.30) |
| Republic of Panama | 0.12(0.09,0.15) | 0.07(0.05,0.09) | 0.53(0.37,0.72) | 0.10(0.07,0.13) | 0.96(0.77,1.15) |
| Republic of Paraguay | 0.05(0.03,0.10) | 0.02(0.01,0.04) | 0.36(0.20,0.59) | 0.05(0.03,0.08) | 2.91(2.67,3.15) |
| Republic of Peru | 0.62(0.36,1.03) | 0.05(0.03,0.08) | 4.43(2.58,7.33) | 0.11(0.06,0.18) | 2.93(2.71,3.16) |
| Republic of Poland | 3.53(2.75,4.54) | 0.06(0.05,0.08) | 13.54(10.99,16.52) | 0.14(0.11,0.17) | 1.88(0.68,3.09) |
| Republic of Rwanda | 0.04(0.02,0.07) | 0.01(0.01,0.02) | 0.16(0.08,0.30) | 0.02(0.01,0.04) | 1.83(1.42,2.24) |
| Republic of San Marino | 0.00(0.00,0.00) | 0.03(0.02,0.05) | 0.00(0.00,0.00) | 0.02(0.01,0.04) | -0.32(-0.76,0.12) |
| Republic of Senegal | 0.01(0.01,0.02) | 0.00(0.00,0.01) | 0.06(0.03,0.10) | 0.01(0.00,0.01) | 2.36(2.12,2.59) |
| Republic of Serbia | 1.70(0.89,2.92) | 0.13(0.07,0.22) | 4.52(2.71,7.16) | 0.20(0.12,0.32) | 1.46(1.27,1.65) |
| Republic of Seychelles | 0.00(0.00,0.00) | 0.01(0.00,0.01) | 0.00(0.00,0.00) | 0.01(0.00,0.02) | 1.54(1.27,1.80) |
| Republic of Sierra Leone | 0.01(0.00,0.01) | 0.00(0.00,0.01) | 0.02(0.01,0.03) | 0.00(0.00,0.01) | 1.78(1.48,2.09) |
| Republic of Singapore | 0.25(0.18,0.34) | 0.10(0.07,0.13) | 1.11(0.76,1.55) | 0.10(0.07,0.14) | 0.09(-0.40,0.59) |
| Republic of Slovenia | 0.24(0.17,0.34) | 0.08(0.05,0.11) | 0.71(0.48,1.03) | 0.12(0.08,0.17) | 1.43(0.69,2.17) |
| Republic of South Africa | 1.24(0.61,1.95) | 0.05(0.03,0.08) | 5.70(3.54,7.82) | 0.11(0.06,0.15) | 2.24(2.02,2.45) |
| Republic of South Sudan | 0.02(0.00,0.04) | 0.01(0.00,0.01) | 0.04(0.01,0.09) | 0.01(0.00,0.02) | 1.95(1.83,2.07) |
| Republic of Sudan | 0.02(0.00,0.08) | 0.00(0.00,0.01) | 0.17(0.07,0.43) | 0.01(0.00,0.02) | 6.46(6.11,6.81) |
| Republic of Suriname | 0.00(0.00,0.01) | 0.01(0.01,0.02) | 0.02(0.01,0.03) | 0.02(0.01,0.04) | 2.99(2.67,3.32) |
| Republic of Tajikistan | 0.00(0.00,0.01) | 0.00(0.00,0.00) | 0.01(0.01,0.02) | 0.00(0.00,0.00) | 0.51(0.25,0.78) |
| Republic of the Congo | 0.03(0.02,0.05) | 0.03(0.01,0.05) | 0.12(0.06,0.21) | 0.05(0.02,0.08) | 1.78(1.51,2.05) |
| Republic of the Gambia | 0.00(0.00,0.00) | 0.01(0.00,0.01) | 0.01(0.01,0.02) | 0.01(0.01,0.02) | 2.51(2.37,2.66) |
| Republic of the Marshall Islands | 0.00(0.00,0.00) | 0.01(0.00,0.02) | 0.00(0.00,0.00) | 0.02(0.01,0.03) | 1.28(1.16,1.40) |
| Republic of the Niger | 0.00(0.00,0.01) | 0.00(0.00,0.00) | 0.02(0.00,0.04) | 0.00(0.00,0.00) | 0.67(0.55,0.79) |
| Republic of the Philippines | 1.20(0.63,1.83) | 0.04(0.02,0.06) | 8.00(5.12,10.97) | 0.08(0.05,0.12) | 2.45(2.33,2.57) |
| Republic of the Union of Myanmar | 0.32(0.15,0.65) | 0.01(0.01,0.02) | 1.88(1.00,3.29) | 0.03(0.02,0.06) | 3.72(3.59,3.86) |
| Republic of Trinidad and Tobago | 0.09(0.06,0.12) | 0.09(0.06,0.12) | 0.45(0.30,0.64) | 0.18(0.12,0.25) | 2.62(2.37,2.87) |
| Republic of Tunisia | 0.21(0.10,0.38) | 0.04(0.02,0.07) | 1.54(0.78,2.71) | 0.09(0.05,0.17) | 3.24(3.04,3.43) |
| Republic of Turkey | 1.83(0.83,3.49) | 0.05(0.02,0.09) | 19.28(11.29,30.34) | 0.17(0.10,0.27) | 4.50(4.06,4.95) |
| Republic of Uganda | 0.04(0.02,0.07) | 0.01(0.00,0.01) | 0.26(0.13,0.45) | 0.02(0.01,0.03) | 3.25(3.06,3.43) |
| Republic of Uzbekistan | 0.96(0.33,1.78) | 0.07(0.02,0.13) | 4.48(2.64,7.03) | 0.15(0.09,0.23) | 2.62(2.35,2.90) |
| Republic of Vanuatu | 0.00(0.00,0.00) | 0.01(0.00,0.02) | 0.00(0.00,0.00) | 0.01(0.00,0.02) | 0.98(0.84,1.12) |
| Republic of Yemen | 0.01(0.00,0.03) | 0.00(0.00,0.01) | 0.09(0.03,0.24) | 0.01(0.00,0.02) | 6.73(6.31,7.16) |
| Republic of Zambia | 0.03(0.02,0.06) | 0.01(0.01,0.02) | 0.31(0.13,0.63) | 0.04(0.02,0.09) | 4.68(4.13,5.23) |
| Republic of Zimbabwe | 0.09(0.05,0.16) | 0.02(0.01,0.03) | 0.21(0.11,0.37) | 0.03(0.01,0.05) | 0.13(-0.35,0.61) |
| Romania | 2.29(1.35,3.64) | 0.06(0.04,0.10) | 5.85(3.77,8.66) | 0.12(0.08,0.17) | 1.99(1.66,2.31) |
| Russian Federation | 25.70(15.45,37.22) | 0.11(0.07,0.16) | 46.20(37.22,56.94) | 0.14(0.11,0.17) | -0.91(-1.75,-0.07) |
| Saint Kitts and Nevis | 0.00(0.00,0.00) | 0.02(0.01,0.04) | 0.00(0.00,0.01) | 0.05(0.03,0.09) | 3.67(3.06,4.29) |
| Saint Lucia | 0.00(0.00,0.01) | 0.03(0.02,0.05) | 0.02(0.01,0.03) | 0.06(0.03,0.10) | 1.87(1.63,2.12) |
| Saint Vincent and the Grenadines | 0.00(0.00,0.00) | 0.00(0.00,0.00) | 0.01(0.01,0.01) | 0.06(0.04,0.08) | 9.84(6.15,13.66) |
| Slovak Republic | 0.62(0.36,0.96) | 0.08(0.05,0.12) | 1.75(1.01,2.75) | 0.14(0.08,0.22) | 1.37(1.01,1.73) |
| Socialist Republic of Viet Nam | 1.12(0.59,2.03) | 0.02(0.01,0.04) | 8.44(4.52,15.36) | 0.07(0.04,0.13) | 3.32(3.01,3.63) |
| Solomon Islands | 0.00(0.00,0.00) | 0.00(0.00,0.02) | 0.00(0.00,0.01) | 0.01(0.00,0.02) | 0.85(0.63,1.07) |
| State of Eritrea | 0.01(0.00,0.02) | 0.01(0.00,0.02) | 0.06(0.03,0.11) | 0.02(0.01,0.04) | 3.00(2.72,3.28) |
| State of Israel | 0.66(0.45,0.93) | 0.10(0.07,0.15) | 2.11(1.45,2.93) | 0.13(0.09,0.18) | 0.70(0.28,1.12) |
| State of Kuwait | 0.02(0.01,0.03) | 0.04(0.03,0.05) | 0.20(0.13,0.30) | 0.08(0.05,0.11) | 4.85(3.05,6.69) |
| State of Libya | 0.08(0.03,0.18) | 0.04(0.01,0.08) | 0.55(0.28,0.98) | 0.10(0.05,0.18) | 3.56(3.05,4.08) |
| State of Qatar | 0.00(0.00,0.00) | 0.01(0.01,0.02) | 0.02(0.01,0.04) | 0.03(0.02,0.06) | 4.14(3.45,4.85) |
| Sultanate of Oman | 0.02(0.00,0.04) | 0.02(0.01,0.05) | 0.11(0.06,0.19) | 0.06(0.03,0.10) | 3.07(2.60,3.55) |
| Swiss Confederation | 1.85(1.34,2.49) | 0.14(0.10,0.18) | 5.43(3.71,7.62) | 0.23(0.16,0.32) | 1.09(0.63,1.55) |
| Syrian Arab Republic | 0.04(0.01,0.09) | 0.01(0.00,0.02) | 0.30(0.14,0.54) | 0.02(0.01,0.04) | 3.59(3.18,4.01) |
| Taiwan (Province of China) | 1.40(1.03,1.87) | 0.07(0.05,0.10) | 8.99(6.18,12.62) | 0.16(0.11,0.23) | 2.18(1.72,2.64) |
| Togolese Republic | 0.00(0.00,0.01) | 0.00(0.00,0.01) | 0.02(0.01,0.04) | 0.01(0.00,0.01) | 1.23(0.99,1.47) |
| Tokelau | 0.00(0.00,0.00) | 0.02(0.01,0.03) | 0.00(0.00,0.00) | 0.02(0.01,0.04) | 1.19(1.01,1.37) |
| Turkmenistan | 0.11(0.06,0.19) | 0.05(0.03,0.09) | 0.54(0.29,0.95) | 0.12(0.06,0.20) | 3.02(2.74,3.30) |
| Tuvalu | 0.00(0.00,0.00) | 0.01(0.00,0.02) | 0.00(0.00,0.00) | 0.01(0.01,0.03) | 1.14(0.78,1.50) |
| Ukraine | 9.31(5.16,14.78) | 0.10(0.05,0.15) | 15.77(9.43,24.14) | 0.15(0.09,0.23) | 1.41(1.06,1.77) |
| Union of the Comoros | 0.00(0.00,0.00) | 0.01(0.00,0.02) | 0.02(0.01,0.03) | 0.03(0.01,0.05) | 3.31(3.18,3.44) |
| United Arab Emirates | 0.01(0.01,0.03) | 0.04(0.02,0.09) | 0.25(0.13,0.41) | 0.11(0.06,0.18) | 4.17(3.54,4.81) |
| United Kingdom of Great Britain and Northern Ireland | 18.67(15.61,22.05) | 0.15(0.13,0.18) | 25.52(21.29,29.65) | 0.15(0.12,0.17) | -0.10(-0.47,0.27) |
| United Mexican States | 1.53(1.22,2.04) | 0.03(0.03,0.04) | 14.09(10.73,18.15) | 0.09(0.07,0.12) | 2.80(1.80,3.81) |
| United Republic of Tanzania | 0.11(0.05,0.23) | 0.01(0.00,0.02) | 0.61(0.30,1.12) | 0.02(0.01,0.04) | 2.91(2.66,3.17) |
| United States of America | 44.66(36.39,53.82) | 0.11(0.09,0.13) | 99.40(82.18,117.16) | 0.13(0.11,0.15) | 0.33(-0.11,0.77) |
| United States Virgin Islands | 0.00(0.00,0.00) | 0.02(0.01,0.04) | 0.01(0.00,0.02) | 0.04(0.02,0.07) | 1.41(0.90,1.92) |

EAPC: Estimated Annual Percentage Change
