# Supplementary material for: Global, regional, and national burden of neuroblastoma and peripheral nervous system tumours in individuals aged over 60 from 1990 to 2021: a trend analysis of global burden of disease study
Source: J Health Popul Nutr. 2025 Mar 17;44:78. doi: 10.1186/s41043-025-00810-9 (PMC11916991; doi:10.1186/s41043-025-00810-9)
Supplement: Supplementary file 12 — Supplementary Material 12 [file 41043_2025_810_MOESM12_ESM.docx]

Supplement 5. The age-standardized incidence rate, number of cases, and EAPC of neuroblastoma and peripheral nervous system tumours among individuals aged 60 and above across 204 countries from 1990 to 2021

| Nation | Incidence (95% UI) | | | | |
| --- | --- | --- | --- | --- | --- |
|  | Cases in 1990 (million) | Age-standardised rate in 1990 (per 100 000) | Cases in 2021(million) | Age-standardised rate in 2021 (per 100 000) | EAPC (95% CI) |
|  |  |  |  |  |  |
| American Samoa | 0.00(0.00,0.01) | 0.09(0.04,0.19) | 0.01(0.00,0.01) | 0.08(0.04,0.17) | -1.18(-2.21,-0.15) |
| Antigua and Barbuda | 0.00(0.00,0.01) | 0.06(0.04,0.10) | 0.02(0.01,0.02) | 0.12(0.07,0.18) | 2.00(1.75,2.24) |
| Arab Republic of Egypt | 0.92(0.38,1.92) | 0.03(0.01,0.07) | 5.01(2.74,9.84) | 0.07(0.04,0.15) | 2.39(1.95,2.82) |
| Argentine Republic | 6.57(4.35,9.53) | 0.16(0.11,0.23) | 18.88(12.87,26.43) | 0.26(0.18,0.37) | 2.17(1.94,2.40) |
| Australia | 6.92(5.16,8.92) | 0.27(0.20,0.35) | 16.79(11.83,22.91) | 0.28(0.20,0.38) | -0.24(-0.49,0.01) |
| Barbados | 0.11(0.08,0.14) | 0.28(0.21,0.38) | 0.33(0.23,0.45) | 0.48(0.33,0.65) | 2.37(2.08,2.66) |
| Belize | 0.01(0.00,0.01) | 0.06(0.03,0.08) | 0.05(0.04,0.07) | 0.15(0.10,0.20) | 3.19(2.25,4.15) |
| Bermuda | 0.01(0.00,0.01) | 0.07(0.05,0.11) | 0.02(0.01,0.03) | 0.11(0.08,0.16) | 1.42(0.99,1.85) |
| Bolivarian Republic of Venezuela | 1.59(1.17,2.19) | 0.14(0.11,0.20) | 8.46(5.58,12.25) | 0.23(0.15,0.33) | 1.16(0.63,1.69) |
| Bosnia and Herzegovina | 0.42(0.22,0.79) | 0.09(0.05,0.17) | 1.69(0.97,2.70) | 0.20(0.11,0.32) | 3.27(2.86,3.69) |
| Brunei Darussalam | 0.01(0.01,0.03) | 0.13(0.06,0.24) | 0.06(0.03,0.09) | 0.14(0.09,0.22) | 0.21(-0.13,0.55) |
| Burkina Faso | 0.02(0.01,0.05) | 0.00(0.00,0.01) | 0.07(0.03,0.14) | 0.01(0.00,0.01) | 1.49(1.33,1.65) |
| Canada | 11.45(8.47,15.26) | 0.27(0.20,0.36) | 24.22(17.04,32.80) | 0.25(0.17,0.34) | -0.33(-0.57,-0.08) |
| Central African Republic | 0.02(0.01,0.06) | 0.02(0.01,0.05) | 0.05(0.02,0.13) | 0.02(0.01,0.06) | 0.76(0.67,0.85) |
| Commonwealth of Dominica | 0.00(0.00,0.01) | 0.06(0.03,0.12) | 0.02(0.01,0.03) | 0.14(0.07,0.25) | 2.72(2.32,3.12) |
| Commonwealth of the Bahamas | 0.02(0.01,0.02) | 0.09(0.06,0.12) | 0.07(0.04,0.09) | 0.13(0.09,0.18) | 1.49(1.32,1.66) |
| Cook Islands | 0.00(0.00,0.00) | 0.03(0.01,0.07) | 0.00(0.00,0.00) | 0.06(0.03,0.11) | 2.15(2.00,2.31) |
| Czech Republic | 4.66(3.21,6.50) | 0.25(0.17,0.35) | 13.51(8.92,19.37) | 0.48(0.31,0.69) | 2.03(1.70,2.37) |
| Democratic People's Republic of Korea | 0.93(0.48,1.74) | 0.05(0.03,0.10) | 3.29(1.56,6.22) | 0.08(0.04,0.16) | 2.15(1.83,2.48) |
| Democratic Republic of Sao Tome and Principe | 0.00(0.00,0.00) | 0.01(0.00,0.01) | 0.00(0.00,0.00) | 0.02(0.01,0.03) | 3.29(3.05,3.53) |
| Democratic Republic of the Congo | 0.37(0.14,0.87) | 0.02(0.01,0.05) | 1.07(0.41,2.51) | 0.03(0.01,0.07) | 0.76(0.17,1.35) |
| Democratic Republic of Timor-Leste | 0.00(0.00,0.01) | 0.01(0.00,0.04) | 0.05(0.02,0.09) | 0.04(0.02,0.09) | 3.90(3.72,4.09) |
| Democratic Socialist Republic of Sri Lanka | 1.38(0.71,2.43) | 0.11(0.06,0.20) | 8.94(4.59,15.32) | 0.25(0.13,0.43) | 2.56(2.32,2.80) |
| Dominican Republic | 0.05(0.02,0.14) | 0.01(0.01,0.04) | 1.06(0.61,1.76) | 0.09(0.05,0.15) | 5.92(5.01,6.84) |
| Eastern Republic of Uruguay | 0.98(0.63,1.47) | 0.19(0.12,0.29) | 2.41(1.57,3.55) | 0.33(0.22,0.49) | 2.05(1.79,2.31) |
| Federal Democratic Republic of Ethiopia | 0.40(0.11,1.29) | 0.02(0.01,0.06) | 2.15(0.82,4.97) | 0.05(0.02,0.11) | 2.97(2.61,3.33) |
| Federal Democratic Republic of Nepal | 0.10(0.03,0.24) | 0.01(0.00,0.02) | 0.76(0.34,1.45) | 0.03(0.01,0.05) | 3.78(3.55,4.02) |
| Federal Republic of Germany | 40.03(29.22,53.18) | 0.24(0.18,0.32) | 85.88(60.78,115.40) | 0.33(0.24,0.45) | 0.72(0.20,1.24) |
| Federal Republic of Nigeria | 2.58(1.30,4.74) | 0.05(0.03,0.10) | 14.56(8.85,21.26) | 0.16(0.10,0.23) | 4.09(3.85,4.33) |
| Federal Republic of Somalia | 0.02(0.00,0.05) | 0.01(0.00,0.02) | 0.06(0.02,0.21) | 0.01(0.00,0.03) | 0.72(0.67,0.77) |
| Federated States of Micronesia | 0.00(0.00,0.00) | 0.02(0.01,0.05) | 0.00(0.00,0.00) | 0.03(0.01,0.06) | 0.54(0.42,0.66) |
| Federative Republic of Brazil | 9.56(7.35,12.76) | 0.09(0.07,0.12) | 65.64(49.61,84.78) | 0.21(0.16,0.27) | 2.27(1.69,2.85) |
| French Republic | 21.96(16.09,29.24) | 0.20(0.15,0.27) | 48.96(32.69,69.06) | 0.26(0.18,0.37) | 0.85(0.47,1.24) |
| Gabonese Republic | 0.04(0.02,0.08) | 0.05(0.02,0.11) | 0.14(0.07,0.25) | 0.13(0.07,0.22) | 2.60(2.50,2.70) |
| Georgia | 0.20(0.12,0.33) | 0.03(0.02,0.04) | 3.96(2.41,5.97) | 0.50(0.30,0.75) | 13.35(11.93,14.80) |
| Grand Duchy of Luxembourg | 0.14(0.11,0.17) | 0.19(0.15,0.24) | 0.30(0.23,0.37) | 0.22(0.17,0.28) | 0.54(0.08,1.01) |
| Greenland | 0.00(0.00,0.01) | 0.13(0.07,0.19) | 0.01(0.00,0.02) | 0.16(0.05,0.29) | 1.26(1.06,1.46) |
| Grenada | 0.00(0.00,0.01) | 0.05(0.03,0.08) | 0.02(0.01,0.03) | 0.16(0.10,0.25) | 3.92(3.53,4.30) |
| Guam | 0.00(0.00,0.00) | 0.04(0.02,0.05) | 0.01(0.01,0.02) | 0.05(0.03,0.07) | 0.97(-0.24,2.19) |
| Hashemite Kingdom of Jordan | 0.12(0.06,0.21) | 0.08(0.04,0.15) | 1.18(0.65,2.01) | 0.15(0.08,0.26) | 2.24(1.98,2.49) |
| Hellenic Republic | 2.32(1.87,2.85) | 0.12(0.09,0.14) | 4.95(3.94,6.13) | 0.16(0.13,0.19) | 0.90(0.62,1.18) |
| Hungary | 3.74(2.53,5.24) | 0.19(0.13,0.27) | 13.37(8.81,19.62) | 0.52(0.34,0.76) | 2.30(1.68,2.93) |
| Independent State of Papua New Guinea | 0.02(0.00,0.06) | 0.01(0.00,0.03) | 0.07(0.02,0.20) | 0.01(0.00,0.04) | 0.92(0.78,1.06) |
| Independent State of Samoa | 0.01(0.00,0.02) | 0.07(0.03,0.27) | 0.02(0.01,0.07) | 0.11(0.05,0.45) | 1.70(1.55,1.85) |
| Ireland | 1.39(1.01,1.84) | 0.26(0.19,0.34) | 2.63(1.84,3.61) | 0.25(0.18,0.35) | -0.44(-0.98,0.12) |
| Islamic Republic of Afghanistan | 0.02(0.00,0.14) | 0.00(0.00,0.02) | 0.09(0.03,0.32) | 0.01(0.00,0.04) | 5.12(4.81,5.42) |
| Islamic Republic of Iran | 0.42(0.09,0.87) | 0.02(0.00,0.03) | 6.50(1.09,9.81) | 0.07(0.01,0.11) | 6.09(5.56,6.63) |
| Islamic Republic of Mauritania | 0.01(0.01,0.02) | 0.01(0.01,0.02) | 0.05(0.03,0.09) | 0.02(0.01,0.04) | 1.76(1.46,2.06) |
| Islamic Republic of Pakistan | 1.89(1.06,3.19) | 0.03(0.02,0.05) | 9.92(5.85,16.09) | 0.08(0.05,0.12) | 2.94(2.81,3.06) |
| Jamaica | 0.20(0.14,0.28) | 0.09(0.06,0.12) | 1.02(0.64,1.57) | 0.26(0.16,0.40) | 3.37(2.61,4.13) |
| Japan | 25.86(21.57,30.51) | 0.12(0.10,0.14) | 107.97(87.29,127.87) | 0.24(0.20,0.28) | 1.47(0.83,2.11) |
| Kingdom of Bahrain | 0.01(0.01,0.02) | 0.06(0.03,0.11) | 0.20(0.10,0.36) | 0.25(0.13,0.44) | 5.68(4.92,6.43) |
| Kingdom of Belgium | 3.97(2.79,5.44) | 0.19(0.14,0.26) | 8.13(5.68,11.15) | 0.26(0.19,0.36) | 0.87(0.32,1.43) |
| Kingdom of Bhutan | 0.00(0.00,0.01) | 0.01(0.00,0.02) | 0.03(0.01,0.05) | 0.04(0.01,0.07) | 4.78(4.63,4.94) |
| Kingdom of Cambodia | 0.12(0.05,0.27) | 0.02(0.01,0.05) | 0.90(0.43,1.71) | 0.06(0.03,0.12) | 3.65(3.47,3.83) |
| Kingdom of Denmark | 1.65(1.19,2.21) | 0.16(0.11,0.21) | 6.54(4.55,8.95) | 0.42(0.29,0.57) | 2.66(2.14,3.19) |
| Kingdom of Eswatini | 0.02(0.01,0.03) | 0.06(0.03,0.11) | 0.09(0.05,0.15) | 0.15(0.08,0.25) | 3.27(2.97,3.58) |
| Kingdom of Lesotho | 0.03(0.02,0.08) | 0.04(0.02,0.08) | 0.12(0.07,0.21) | 0.10(0.05,0.17) | 3.75(3.49,4.02) |
| Kingdom of Morocco | 0.74(0.34,1.48) | 0.04(0.02,0.09) | 5.64(2.96,10.03) | 0.14(0.07,0.24) | 4.01(3.74,4.29) |
| Kingdom of Norway | 2.34(1.88,2.86) | 0.25(0.20,0.31) | 4.41(3.51,5.40) | 0.33(0.27,0.41) | -0.29(-0.86,0.29) |
| Kingdom of Saudi Arabia | 0.26(0.07,0.50) | 0.04(0.01,0.08) | 2.19(0.97,3.67) | 0.13(0.06,0.22) | 3.85(2.84,4.87) |
| Kingdom of Spain | 15.85(11.45,21.10) | 0.22(0.16,0.29) | 36.60(25.19,51.26) | 0.28(0.20,0.40) | 0.69(0.28,1.11) |
| Kingdom of Sweden | 3.56(2.58,4.78) | 0.18(0.13,0.24) | 7.95(5.46,11.07) | 0.28(0.19,0.39) | 1.12(-0.04,2.29) |
| Kingdom of Thailand | 3.66(2.07,6.01) | 0.09(0.05,0.15) | 29.23(17.16,48.19) | 0.21(0.12,0.34) | 2.41(2.26,2.56) |
| Kingdom of the Netherlands | 10.10(7.47,13.36) | 0.39(0.29,0.51) | 18.66(13.22,25.51) | 0.40(0.28,0.54) | -0.32(-0.70,0.06) |
| Kingdom of Tonga | 0.00(0.00,0.00) | 0.02(0.01,0.05) | 0.00(0.00,0.01) | 0.03(0.01,0.07) | 1.34(1.07,1.61) |
| Kyrgyz Republic | 0.08(0.05,0.13) | 0.02(0.01,0.04) | 0.87(0.56,1.28) | 0.16(0.10,0.23) | 8.59(7.37,9.84) |
| Lao People's Democratic Republic | 0.05(0.02,0.11) | 0.02(0.01,0.04) | 0.28(0.13,0.56) | 0.06(0.03,0.11) | 3.81(3.72,3.90) |
| Lebanese Republic | 0.14(0.08,0.25) | 0.06(0.03,0.10) | 0.92(0.51,1.53) | 0.12(0.07,0.20) | 3.53(3.19,3.88) |
| Malaysia | 1.25(0.64,2.31) | 0.12(0.06,0.22) | 10.28(6.05,16.34) | 0.30(0.17,0.47) | 2.69(2.29,3.09) |
| Mongolia | 0.10(0.05,0.19) | 0.08(0.04,0.16) | 0.58(0.33,0.93) | 0.23(0.13,0.38) | 3.68(3.45,3.91) |
| Montenegro | 0.09(0.06,0.14) | 0.12(0.07,0.18) | 0.33(0.23,0.46) | 0.26(0.18,0.36) | 2.64(2.32,2.97) |
| New Zealand | 1.47(1.07,1.98) | 0.28(0.21,0.38) | 3.67(2.68,4.93) | 0.33(0.24,0.44) | 0.35(-0.35,1.06) |
| North Macedonia | 0.19(0.12,0.27) | 0.08(0.05,0.12) | 0.83(0.56,1.18) | 0.20(0.13,0.28) | 3.06(2.52,3.60) |
| Northern Mariana Islands | 0.00(0.00,0.00) | 0.01(0.00,0.02) | 0.00(0.00,0.00) | 0.02(0.01,0.03) | 1.28(0.32,2.24) |
| Palestine | 0.07(0.03,0.15) | 0.07(0.03,0.14) | 0.47(0.27,0.75) | 0.18(0.10,0.28) | 3.23(3.04,3.42) |
| People's Democratic Republic of Algeria | 0.43(0.22,0.84) | 0.03(0.02,0.06) | 3.14(1.70,5.66) | 0.08(0.04,0.14) | 2.42(2.22,2.62) |
| People's Republic of Bangladesh | 0.70(0.24,1.63) | 0.01(0.00,0.03) | 6.49(2.80,12.42) | 0.04(0.02,0.07) | 3.40(3.09,3.72) |
| People's Republic of China | 56.04(35.03,88.08) | 0.06(0.04,0.10) | 778.39(506.97,1022.69) | 0.30(0.19,0.39) | 6.06(5.68,6.43) |
| Plurinational State of Bolivia | 0.29(0.16,0.49) | 0.08(0.04,0.14) | 2.09(1.11,3.65) | 0.19(0.10,0.34) | 3.10(2.96,3.23) |
| Portuguese Republic | 3.67(2.64,4.94) | 0.20(0.14,0.27) | 7.76(5.31,10.86) | 0.23(0.16,0.33) | 0.55(0.23,0.88) |
| Principality of Andorra | 0.02(0.01,0.03) | 0.22(0.11,0.40) | 0.05(0.02,0.08) | 0.24(0.12,0.42) | 0.69(0.46,0.93) |
| Principality of Monaco | 0.00(0.00,0.00) | 0.00(0.00,0.01) | 0.00(0.00,0.00) | 0.00(0.00,0.01) | 0.61(0.55,0.68) |
| Puerto Rico | 0.44(0.29,0.65) | 0.10(0.06,0.14) | 1.75(1.15,2.55) | 0.19(0.12,0.27) | 2.08(1.58,2.58) |
| Republic of Albania | 0.06(0.03,0.09) | 0.02(0.01,0.04) | 0.31(0.17,0.51) | 0.05(0.03,0.09) | 3.06(2.74,3.38) |
| Republic of Angola | 0.08(0.02,0.19) | 0.02(0.01,0.05) | 0.61(0.23,1.33) | 0.05(0.02,0.11) | 2.78(2.51,3.04) |
| Republic of Armenia | 0.39(0.19,0.67) | 0.12(0.06,0.20) | 3.26(2.06,4.95) | 0.55(0.35,0.84) | 6.47(5.66,7.29) |
| Republic of Austria | 3.50(2.60,4.61) | 0.22(0.16,0.29) | 5.56(3.93,7.53) | 0.24(0.17,0.32) | 0.23(-0.26,0.71) |
| Republic of Azerbaijan | 0.58(0.28,1.11) | 0.10(0.05,0.20) | 1.86(1.02,3.22) | 0.16(0.09,0.28) | 2.26(1.80,2.73) |
| Republic of Belarus | 2.36(1.51,3.56) | 0.14(0.09,0.21) | 13.09(8.25,19.62) | 0.60(0.38,0.89) | 4.05(3.53,4.57) |
| Republic of Benin | 0.01(0.01,0.03) | 0.01(0.00,0.01) | 0.05(0.03,0.10) | 0.01(0.01,0.02) | 1.39(1.20,1.59) |
| Republic of Botswana | 0.04(0.02,0.07) | 0.06(0.03,0.12) | 0.24(0.13,0.43) | 0.15(0.08,0.28) | 3.27(2.97,3.57) |
| Republic of Bulgaria | 1.17(0.72,1.78) | 0.07(0.05,0.11) | 3.63(2.35,5.46) | 0.19(0.12,0.29) | 2.23(1.56,2.91) |
| Republic of Burundi | 0.04(0.02,0.08) | 0.02(0.01,0.03) | 0.10(0.04,0.24) | 0.02(0.01,0.05) | 0.72(0.52,0.91) |
| Republic of Cabo Verde | 0.00(0.00,0.00) | 0.00(0.00,0.00) | 0.00(0.00,0.01) | 0.01(0.00,0.01) | 5.13(4.94,5.33) |
| Republic of Cameroon | 0.05(0.03,0.08) | 0.01(0.01,0.02) | 0.21(0.11,0.37) | 0.02(0.01,0.03) | 1.47(1.25,1.69) |
| Republic of Chad | 0.01(0.00,0.02) | 0.00(0.00,0.01) | 0.03(0.01,0.07) | 0.01(0.00,0.01) | 1.78(1.68,1.88) |
| Republic of Chile | 0.65(0.45,0.92) | 0.05(0.04,0.08) | 7.00(4.80,9.71) | 0.21(0.15,0.29) | 4.80(3.51,6.11) |
| Republic of Colombia | 1.64(1.11,2.37) | 0.08(0.06,0.12) | 14.73(9.72,21.29) | 0.21(0.14,0.31) | 2.20(1.47,2.93) |
| Republic of Costa Rica | 0.21(0.14,0.30) | 0.10(0.07,0.15) | 1.87(1.25,2.62) | 0.27(0.18,0.38) | 2.43(1.82,3.04) |
| Republic of Croatia | 2.37(1.64,3.27) | 0.31(0.22,0.43) | 9.09(6.12,12.77) | 0.75(0.50,1.05) | 2.70(2.37,3.04) |
| Republic of Cuba | 1.57(1.07,2.26) | 0.12(0.08,0.18) | 5.17(3.60,7.32) | 0.21(0.15,0.30) | 2.68(2.10,3.27) |
| Republic of Cyprus | 0.28(0.13,0.51) | 0.31(0.15,0.56) | 1.06(0.64,1.66) | 0.39(0.24,0.61) | 0.85(0.57,1.13) |
| The Republic of Côte d'Ivoire | 0.02(0.01,0.04) | 0.01(0.00,0.01) | 0.11(0.05,0.20) | 0.01(0.00,0.02) | 1.52(1.32,1.72) |
| Republic of Djibouti | 0.00(0.00,0.01) | 0.03(0.01,0.05) | 0.04(0.02,0.07) | 0.06(0.03,0.11) | 2.96(2.83,3.09) |
| Republic of Ecuador | 0.49(0.33,0.73) | 0.08(0.05,0.12) | 5.54(3.48,8.42) | 0.28(0.17,0.42) | 4.61(3.92,5.30) |
| Republic of El Salvador | 0.13(0.07,0.20) | 0.04(0.02,0.06) | 0.62(0.39,0.94) | 0.08(0.05,0.12) | 2.51(2.18,2.83) |
| Republic of Equatorial Guinea | 0.00(0.00,0.01) | 0.02(0.01,0.05) | 0.07(0.03,0.13) | 0.14(0.07,0.25) | 6.64(6.48,6.79) |
| Republic of Estonia | 0.46(0.29,0.70) | 0.17(0.11,0.26) | 2.98(1.97,4.40) | 0.85(0.56,1.25) | 3.57(2.87,4.28) |
| Republic of Fiji | 0.04(0.02,0.08) | 0.11(0.05,0.24) | 0.09(0.03,0.23) | 0.10(0.04,0.26) | -1.45(-2.05,-0.85) |
| Republic of Finland | 1.47(1.03,2.03) | 0.16(0.11,0.21) | 7.28(4.90,10.24) | 0.43(0.29,0.60) | 3.21(2.66,3.77) |
| Republic of Ghana | 0.06(0.01,0.12) | 0.01(0.00,0.02) | 0.14(0.07,0.25) | 0.01(0.00,0.02) | -2.11(-3.26,-0.94) |
| Republic of Guatemala | 0.09(0.07,0.13) | 0.03(0.02,0.04) | 0.46(0.33,0.63) | 0.04(0.03,0.05) | 0.71(0.38,1.03) |
| Republic of Guinea | 0.03(0.01,0.05) | 0.01(0.00,0.01) | 0.08(0.04,0.16) | 0.01(0.01,0.03) | 2.05(1.98,2.13) |
| Republic of Guinea-Bissau | 0.00(0.00,0.01) | 0.01(0.00,0.01) | 0.01(0.00,0.01) | 0.01(0.01,0.02) | 1.16(1.06,1.27) |
| Republic of Guyana | 0.00(0.00,0.00) | 0.00(0.00,0.01) | 0.06(0.04,0.09) | 0.08(0.05,0.12) | 8.01(5.68,10.38) |
| Republic of Haiti | 0.08(0.03,0.18) | 0.02(0.01,0.05) | 0.32(0.14,0.65) | 0.04(0.02,0.08) | 2.12(1.93,2.32) |
| Republic of Honduras | 0.09(0.04,0.19) | 0.04(0.02,0.08) | 1.13(0.61,1.97) | 0.15(0.08,0.27) | 4.81(4.43,5.20) |
| Republic of Iceland | 0.09(0.07,0.12) | 0.25(0.18,0.33) | 0.28(0.20,0.39) | 0.37(0.25,0.50) | 1.42(1.18,1.66) |
| Republic of India | 11.27(6.52,18.33) | 0.02(0.01,0.04) | 91.37(66.91,125.34) | 0.06(0.05,0.09) | 3.14(2.81,3.47) |
| Republic of Indonesia | 3.89(2.36,6.17) | 0.04(0.02,0.06) | 34.72(23.02,50.20) | 0.13(0.08,0.19) | 3.94(3.80,4.08) |
| Republic of Iraq | 0.22(0.09,0.45) | 0.02(0.01,0.05) | 2.37(1.29,4.01) | 0.09(0.05,0.16) | 4.27(4.02,4.53) |
| Republic of Italy | 23.58(19.20,27.97) | 0.20(0.16,0.24) | 55.91(43.76,68.16) | 0.29(0.23,0.36) | 1.41(1.06,1.76) |
| Republic of Kazakhstan | 1.17(0.64,1.87) | 0.08(0.04,0.12) | 3.67(2.15,5.78) | 0.17(0.10,0.27) | 2.37(2.00,2.75) |
| Republic of Kenya | 0.10(0.04,0.21) | 0.01(0.00,0.02) | 0.95(0.54,1.49) | 0.04(0.02,0.06) | 3.93(3.80,4.07) |
| Republic of Kiribati | 0.00(0.00,0.00) | 0.00(0.00,0.01) | 0.00(0.00,0.00) | 0.01(0.00,0.01) | 1.27(1.19,1.34) |
| Republic of Korea | 4.42(2.56,7.23) | 0.14(0.08,0.22) | 19.49(11.70,30.10) | 0.16(0.09,0.24) | -0.02(-0.40,0.35) |
| Republic of Latvia | 0.58(0.35,0.89) | 0.12(0.08,0.19) | 1.74(1.08,2.68) | 0.34(0.21,0.52) | 3.49(3.19,3.80) |
| Republic of Liberia | 0.01(0.00,0.02) | 0.01(0.00,0.01) | 0.02(0.01,0.03) | 0.01(0.00,0.02) | 1.23(0.90,1.56) |
| Republic of Lithuania | 0.75(0.49,1.10) | 0.13(0.08,0.19) | 4.10(2.57,6.15) | 0.55(0.34,0.83) | 4.95(4.69,5.22) |
| Republic of Madagascar | 0.10(0.05,0.20) | 0.02(0.01,0.03) | 0.33(0.16,0.61) | 0.03(0.01,0.05) | 1.57(1.25,1.90) |
| Republic of Malawi | 0.16(0.08,0.30) | 0.04(0.02,0.07) | 0.65(0.35,1.09) | 0.08(0.04,0.13) | 2.58(2.44,2.72) |
| Republic of Maldives | 0.01(0.00,0.03) | 0.13(0.05,0.29) | 0.14(0.08,0.24) | 0.42(0.24,0.69) | 3.76(3.26,4.26) |
| Republic of Mali | 0.02(0.01,0.03) | 0.00(0.00,0.01) | 0.06(0.03,0.12) | 0.01(0.00,0.01) | 1.95(1.83,2.06) |
| Republic of Malta | 0.22(0.16,0.30) | 0.40(0.29,0.55) | 0.67(0.47,0.93) | 0.51(0.36,0.70) | 0.67(0.15,1.20) |
| Republic of Mauritius | 0.07(0.05,0.09) | 0.08(0.06,0.11) | 0.56(0.41,0.73) | 0.23(0.17,0.30) | 3.26(2.28,4.24) |
| Republic of Moldova | 0.43(0.29,0.63) | 0.08(0.05,0.11) | 2.04(1.55,2.67) | 0.25(0.19,0.33) | 4.66(4.22,5.11) |
| Republic of Mozambique | 0.08(0.03,0.20) | 0.01(0.00,0.03) | 0.35(0.16,0.79) | 0.03(0.01,0.07) | 3.24(3.05,3.43) |
| Republic of Namibia | 0.03(0.01,0.06) | 0.04(0.02,0.08) | 0.14(0.07,0.27) | 0.09(0.04,0.18) | 2.47(2.30,2.65) |
| Republic of Nauru | 0.00(0.00,0.00) | 0.04(0.01,0.09) | 0.00(0.00,0.00) | 0.05(0.02,0.08) | 0.54(0.18,0.90) |
| Republic of Nicaragua | 0.15(0.08,0.25) | 0.09(0.05,0.15) | 1.01(0.67,1.48) | 0.18(0.12,0.26) | 2.45(1.99,2.92) |
| Republic of Niue | 0.00(0.00,0.00) | 0.04(0.02,0.07) | 0.00(0.00,0.00) | 0.05(0.03,0.10) | 1.20(0.91,1.50) |
| Republic of Palau | 0.00(0.00,0.00) | 0.01(0.01,0.03) | 0.00(0.00,0.00) | 0.02(0.01,0.03) | 0.14(-0.02,0.30) |
| Republic of Panama | 0.23(0.17,0.31) | 0.13(0.10,0.18) | 1.06(0.74,1.43) | 0.20(0.14,0.26) | 0.96(0.77,1.15) |
| Republic of Paraguay | 0.11(0.05,0.19) | 0.04(0.02,0.07) | 0.73(0.41,1.18) | 0.10(0.06,0.17) | 2.91(2.67,3.15) |
| Republic of Peru | 1.24(0.72,2.06) | 0.09(0.05,0.15) | 8.87(5.15,14.66) | 0.22(0.13,0.36) | 2.93(2.71,3.16) |
| Republic of Poland | 7.07(5.49,9.07) | 0.12(0.10,0.16) | 27.09(21.98,33.04) | 0.27(0.22,0.33) | 1.88(0.68,3.09) |
| Republic of Rwanda | 0.08(0.04,0.13) | 0.02(0.01,0.04) | 0.32(0.16,0.61) | 0.05(0.02,0.09) | 1.83(1.42,2.24) |
| Republic of San Marino | 0.00(0.00,0.00) | 0.06(0.04,0.10) | 0.00(0.00,0.01) | 0.04(0.02,0.08) | -0.32(-0.76,0.12) |
| Republic of Senegal | 0.02(0.01,0.05) | 0.01(0.00,0.01) | 0.12(0.06,0.21) | 0.01(0.01,0.03) | 2.36(2.12,2.59) |
| Republic of Serbia | 3.39(1.78,5.85) | 0.25(0.13,0.43) | 9.03(5.43,14.32) | 0.40(0.24,0.64) | 1.46(1.27,1.65) |
| Republic of Seychelles | 0.00(0.00,0.00) | 0.01(0.00,0.03) | 0.00(0.00,0.01) | 0.02(0.00,0.05) | 1.54(1.27,1.80) |
| Republic of Sierra Leone | 0.01(0.01,0.02) | 0.01(0.00,0.01) | 0.04(0.02,0.07) | 0.01(0.00,0.02) | 1.78(1.48,2.09) |
| Republic of Singapore | 0.50(0.36,0.67) | 0.19(0.14,0.26) | 2.22(1.52,3.10) | 0.20(0.14,0.28) | 0.09(-0.40,0.59) |
| Republic of Slovenia | 0.49(0.34,0.67) | 0.16(0.11,0.21) | 1.42(0.96,2.05) | 0.24(0.16,0.35) | 1.43(0.69,2.17) |
| Republic of South Africa | 2.47(1.22,3.89) | 0.11(0.05,0.17) | 11.41(7.08,15.64) | 0.21(0.13,0.29) | 2.24(2.02,2.45) |
| Republic of South Sudan | 0.03(0.01,0.09) | 0.01(0.00,0.03) | 0.08(0.03,0.18) | 0.02(0.01,0.05) | 1.95(1.83,2.07) |
| Republic of Sudan | 0.03(0.01,0.16) | 0.00(0.00,0.02) | 0.34(0.15,0.86) | 0.02(0.01,0.04) | 6.46(6.11,6.81) |
| Republic of Suriname | 0.01(0.00,0.01) | 0.03(0.01,0.05) | 0.04(0.02,0.07) | 0.05(0.03,0.09) | 2.99(2.67,3.32) |
| Republic of Tajikistan | 0.01(0.00,0.02) | 0.00(0.00,0.01) | 0.02(0.01,0.04) | 0.00(0.00,0.01) | 0.51(0.25,0.78) |
| Republic of the Congo | 0.06(0.03,0.11) | 0.05(0.03,0.09) | 0.25(0.12,0.43) | 0.09(0.05,0.16) | 1.78(1.51,2.05) |
| Republic of the Gambia | 0.00(0.00,0.01) | 0.01(0.01,0.02) | 0.02(0.01,0.05) | 0.02(0.01,0.05) | 2.51(2.37,2.66) |
| Republic of the Marshall Islands | 0.00(0.00,0.00) | 0.02(0.01,0.05) | 0.00(0.00,0.00) | 0.03(0.01,0.06) | 1.28(1.16,1.40) |
| Republic of the Niger | 0.01(0.00,0.02) | 0.00(0.00,0.01) | 0.03(0.01,0.07) | 0.00(0.00,0.01) | 0.67(0.55,0.79) |
| Republic of the Philippines | 2.41(1.26,3.66) | 0.08(0.04,0.12) | 16.01(10.25,21.95) | 0.17(0.11,0.23) | 2.45(2.33,2.57) |
| Republic of the Union of Myanmar | 0.63(0.31,1.30) | 0.02(0.01,0.05) | 3.75(1.99,6.58) | 0.07(0.03,0.11) | 3.72(3.59,3.86) |
| Republic of Trinidad and Tobago | 0.18(0.12,0.25) | 0.18(0.12,0.25) | 0.90(0.61,1.29) | 0.35(0.24,0.51) | 2.62(2.37,2.87) |
| Republic of Tunisia | 0.42(0.20,0.77) | 0.07(0.03,0.13) | 3.08(1.55,5.42) | 0.19(0.10,0.33) | 3.24(3.04,3.43) |
| Republic of Turkey | 3.66(1.66,6.98) | 0.09(0.04,0.18) | 38.56(22.59,60.68) | 0.34(0.20,0.53) | 4.50(4.06,4.95) |
| Republic of Uganda | 0.08(0.05,0.15) | 0.01(0.01,0.02) | 0.51(0.26,0.90) | 0.03(0.02,0.06) | 3.25(3.06,3.43) |
| Republic of Uzbekistan | 1.91(0.66,3.55) | 0.14(0.05,0.26) | 8.95(5.29,14.07) | 0.29(0.17,0.45) | 2.62(2.35,2.90) |
| Republic of Vanuatu | 0.00(0.00,0.00) | 0.01(0.00,0.03) | 0.00(0.00,0.01) | 0.02(0.01,0.04) | 0.98(0.84,1.12) |
| Republic of Yemen | 0.01(0.00,0.06) | 0.00(0.00,0.01) | 0.17(0.06,0.48) | 0.01(0.00,0.03) | 6.73(6.31,7.16) |
| Republic of Zambia | 0.07(0.03,0.13) | 0.02(0.01,0.04) | 0.62(0.27,1.25) | 0.09(0.04,0.17) | 4.68(4.13,5.23) |
| Republic of Zimbabwe | 0.18(0.10,0.31) | 0.04(0.02,0.07) | 0.41(0.22,0.73) | 0.05(0.03,0.10) | 0.13(-0.35,0.61) |
| Romania | 4.57(2.70,7.28) | 0.13(0.08,0.20) | 11.71(7.54,17.31) | 0.24(0.15,0.35) | 1.99(1.66,2.31) |
| Russian Federation | 51.39(30.89,74.45) | 0.22(0.13,0.31) | 92.41(74.44,113.88) | 0.28(0.23,0.35) | -0.91(-1.75,-0.07) |
| Saint Kitts and Nevis | 0.00(0.00,0.00) | 0.05(0.03,0.08) | 0.01(0.01,0.02) | 0.11(0.06,0.19) | 3.67(3.06,4.29) |
| Saint Lucia | 0.01(0.00,0.01) | 0.06(0.03,0.09) | 0.04(0.02,0.06) | 0.12(0.07,0.19) | 1.87(1.63,2.12) |
| Saint Vincent and the Grenadines | 0.00(0.00,0.00) | 0.00(0.00,0.00) | 0.02(0.01,0.03) | 0.12(0.08,0.16) | 9.84(6.15,13.66) |
| Slovak Republic | 1.24(0.73,1.92) | 0.16(0.09,0.24) | 3.49(2.02,5.51) | 0.27(0.16,0.43) | 1.37(1.01,1.73) |
| Socialist Republic of Viet Nam | 2.23(1.18,4.07) | 0.05(0.02,0.08) | 16.88(9.04,30.71) | 0.14(0.08,0.26) | 3.32(3.01,3.63) |
| Solomon Islands | 0.00(0.00,0.00) | 0.01(0.00,0.03) | 0.01(0.00,0.01) | 0.02(0.01,0.04) | 0.85(0.63,1.07) |
| State of Eritrea | 0.02(0.01,0.04) | 0.02(0.01,0.03) | 0.12(0.06,0.21) | 0.04(0.02,0.08) | 3.00(2.72,3.28) |
| State of Israel | 1.32(0.89,1.86) | 0.21(0.14,0.29) | 4.23(2.91,5.86) | 0.26(0.18,0.36) | 0.70(0.28,1.12) |
| State of Kuwait | 0.04(0.03,0.06) | 0.08(0.05,0.11) | 0.41(0.26,0.60) | 0.15(0.10,0.22) | 4.85(3.05,6.69) |
| State of Libya | 0.16(0.06,0.35) | 0.08(0.03,0.17) | 1.10(0.56,1.96) | 0.20(0.10,0.36) | 3.56(3.05,4.08) |
| State of Qatar | 0.00(0.00,0.00) | 0.03(0.01,0.05) | 0.04(0.02,0.07) | 0.07(0.03,0.11) | 4.14(3.45,4.85) |
| Sultanate of Oman | 0.03(0.01,0.07) | 0.04(0.01,0.10) | 0.22(0.12,0.37) | 0.12(0.06,0.20) | 3.07(2.60,3.55) |
| Swiss Confederation | 3.70(2.69,4.98) | 0.27(0.20,0.37) | 10.87(7.42,15.25) | 0.46(0.31,0.64) | 1.09(0.63,1.55) |
| Syrian Arab Republic | 0.07(0.02,0.18) | 0.01(0.00,0.03) | 0.60(0.29,1.09) | 0.04(0.02,0.07) | 3.59(3.18,4.01) |
| Taiwan (Province of China) | 2.80(2.05,3.74) | 0.14(0.11,0.19) | 17.98(12.37,25.25) | 0.32(0.22,0.45) | 2.18(1.72,2.64) |
| Togolese Republic | 0.01(0.01,0.02) | 0.01(0.00,0.01) | 0.04(0.02,0.08) | 0.01(0.01,0.02) | 1.23(0.99,1.47) |
| Tokelau | 0.00(0.00,0.00) | 0.03(0.01,0.07) | 0.00(0.00,0.00) | 0.05(0.02,0.09) | 1.19(1.01,1.37) |
| Turkmenistan | 0.23(0.12,0.38) | 0.10(0.05,0.17) | 1.09(0.59,1.91) | 0.23(0.13,0.40) | 3.02(2.74,3.30) |
| Tuvalu | 0.00(0.00,0.00) | 0.02(0.01,0.04) | 0.00(0.00,0.00) | 0.03(0.01,0.06) | 1.14(0.78,1.50) |
| Ukraine | 18.62(10.32,29.56) | 0.19(0.10,0.30) | 31.55(18.86,48.27) | 0.30(0.18,0.45) | 1.41(1.06,1.77) |
| Union of the Comoros | 0.00(0.00,0.01) | 0.02(0.01,0.04) | 0.03(0.02,0.06) | 0.06(0.03,0.10) | 3.31(3.18,3.44) |
| United Arab Emirates | 0.03(0.01,0.06) | 0.08(0.03,0.17) | 0.49(0.26,0.82) | 0.22(0.12,0.36) | 4.17(3.54,4.81) |
| United Kingdom of Great Britain and Northern Ireland | 37.35(31.22,44.10) | 0.31(0.26,0.36) | 51.05(42.58,59.30) | 0.29(0.25,0.34) | -0.10(-0.47,0.27) |
| United Mexican States | 3.07(2.44,4.08) | 0.07(0.05,0.09) | 28.18(21.47,36.30) | 0.19(0.14,0.24) | 2.80(1.80,3.81) |
| United Republic of Tanzania | 0.23(0.10,0.46) | 0.02(0.01,0.04) | 1.22(0.60,2.24) | 0.04(0.02,0.08) | 2.91(2.66,3.17) |
| United States of America | 89.32(72.79,107.64) | 0.21(0.17,0.26) | 198.80(164.36,234.31) | 0.25(0.21,0.30) | 0.33(-0.11,0.77) |
| United States Virgin Islands | 0.00(0.00,0.01) | 0.05(0.03,0.09) | 0.02(0.01,0.03) | 0.08(0.04,0.14) | 1.41(0.90,1.92) |

EAPC: Estimated Annual Percentage Change
