# Supplementary material for: Global, regional, and national burden of neuroblastoma and peripheral nervous system tumours in individuals aged over 60 from 1990 to 2021: a trend analysis of global burden of disease study
Source: J Health Popul Nutr. 2025 Mar 17;44:78. doi: 10.1186/s41043-025-00810-9 (PMC11916991; doi:10.1186/s41043-025-00810-9)
Supplement: Supplementary file 13 — Supplementary Material 13 [file 41043_2025_810_MOESM13_ESM.docx]

Supplement 6. The age-standardized mortality rate, number of cases, and EAPC of neuroblastoma and peripheral nervous system tumours among individuals aged 60 and above across 204 countries from 1990 to 2021

| Nation | Deaths (95% UI) | | | | |
| --- | --- | --- | --- | --- | --- |
|  | Cases in 1990 (million) | Age-standardised rate in 1990 (per 100 000) | Cases in 2021(million) | Age-standardised rate in 2021 (per 100 000) | EAPC (95% CI) |
|  |  |  |  |  |  |
| American Samoa | 0.00(0.00,0.00) | 0.08(0.03,0.15) | 0.00(0.00,0.01) | 0.06(0.03,0.12) | -1.46(-2.41,-0.51) |
| Antigua and Barbuda | 0.00(0.00,0.01) | 0.05(0.03,0.07) | 0.01(0.01,0.02) | 0.08(0.05,0.12) | 1.69(1.40,1.98) |
| Arab Republic of Egypt | 0.83(0.37,1.69) | 0.03(0.01,0.06) | 3.94(2.25,7.42) | 0.06(0.04,0.12) | 1.81(1.44,2.17) |
| Argentine Republic | 5.34(3.80,7.13) | 0.13(0.09,0.18) | 13.42(9.70,17.95) | 0.19(0.13,0.25) | 1.56(1.28,1.85) |
| Australia | 4.26(3.28,5.34) | 0.17(0.13,0.21) | 10.62(7.62,14.14) | 0.17(0.13,0.23) | -0.21(-0.46,0.04) |
| Barbados | 0.09(0.07,0.11) | 0.23(0.19,0.28) | 0.23(0.17,0.30) | 0.34(0.25,0.44) | 1.72(1.54,1.90) |
| Belize | 0.01(0.00,0.01) | 0.05(0.03,0.07) | 0.04(0.03,0.05) | 0.12(0.09,0.14) | 2.71(1.82,3.61) |
| Bermuda | 0.00(0.00,0.01) | 0.06(0.05,0.08) | 0.01(0.01,0.02) | 0.07(0.05,0.09) | 0.57(0.12,1.03) |
| Bolivarian Republic of Venezuela | 1.47(1.21,1.78) | 0.13(0.11,0.16) | 6.84(4.92,9.12) | 0.19(0.14,0.25) | 1.04(0.57,1.51) |
| Bosnia and Herzegovina | 0.34(0.18,0.61) | 0.07(0.04,0.14) | 1.20(0.73,1.83) | 0.14(0.09,0.22) | 2.68(2.42,2.93) |
| Brunei Darussalam | 0.01(0.01,0.02) | 0.10(0.05,0.18) | 0.04(0.02,0.06) | 0.10(0.06,0.15) | 0.10(-0.30,0.51) |
| Burkina Faso | 0.02(0.01,0.05) | 0.00(0.00,0.01) | 0.07(0.03,0.14) | 0.01(0.00,0.01) | 1.57(1.42,1.71) |
| Canada | 7.04(5.35,9.04) | 0.17(0.13,0.21) | 15.18(11.12,19.97) | 0.15(0.11,0.20) | -0.30(-0.55,-0.05) |
| Central African Republic | 0.02(0.01,0.06) | 0.02(0.01,0.05) | 0.05(0.02,0.12) | 0.02(0.01,0.06) | 0.78(0.70,0.87) |
| Commonwealth of Dominica | 0.00(0.00,0.01) | 0.06(0.03,0.11) | 0.01(0.01,0.02) | 0.12(0.06,0.20) | 2.25(2.02,2.49) |
| Commonwealth of the Bahamas | 0.01(0.01,0.02) | 0.07(0.06,0.09) | 0.05(0.04,0.07) | 0.11(0.08,0.14) | 1.56(1.38,1.73) |
| Cook Islands | 0.00(0.00,0.00) | 0.03(0.01,0.06) | 0.00(0.00,0.00) | 0.05(0.02,0.08) | 1.48(1.35,1.61) |
| Czech Republic | 3.24(2.36,4.32) | 0.18(0.13,0.24) | 8.38(5.70,11.96) | 0.29(0.20,0.41) | 1.33(0.95,1.71) |
| Democratic People's Republic of Korea | 0.84(0.46,1.48) | 0.05(0.03,0.09) | 2.64(1.31,4.86) | 0.07(0.03,0.12) | 1.52(1.29,1.74) |
| Democratic Republic of Sao Tome and Principe | 0.00(0.00,0.00) | 0.01(0.00,0.01) | 0.00(0.00,0.00) | 0.02(0.01,0.03) | 2.78(2.57,2.99) |
| Democratic Republic of the Congo | 0.37(0.14,0.83) | 0.02(0.01,0.05) | 1.05(0.42,2.42) | 0.03(0.01,0.07) | 0.77(0.18,1.36) |
| Democratic Republic of Timor-Leste | 0.00(0.00,0.01) | 0.01(0.00,0.04) | 0.04(0.02,0.08) | 0.04(0.02,0.08) | 3.47(3.32,3.63) |
| Democratic Socialist Republic of Sri Lanka | 1.28(0.71,2.12) | 0.11(0.06,0.18) | 6.30(3.34,10.46) | 0.18(0.10,0.30) | 1.86(1.65,2.08) |
| Dominican Republic | 0.05(0.02,0.13) | 0.01(0.01,0.03) | 0.85(0.51,1.34) | 0.07(0.04,0.11) | 5.33(4.52,6.14) |
| Eastern Republic of Uruguay | 0.80(0.56,1.12) | 0.16(0.11,0.22) | 1.74(1.20,2.43) | 0.24(0.16,0.33) | 1.50(1.35,1.65) |
| Federal Democratic Republic of Ethiopia | 0.39(0.12,1.29) | 0.02(0.01,0.06) | 1.97(0.79,4.50) | 0.04(0.02,0.10) | 2.69(2.41,2.98) |
| Federal Democratic Republic of Nepal | 0.10(0.03,0.23) | 0.01(0.00,0.02) | 0.71(0.33,1.31) | 0.03(0.01,0.05) | 3.39(3.17,3.61) |
| Federal Republic of Germany | 28.86(22.12,36.46) | 0.17(0.13,0.22) | 54.36(40.20,71.50) | 0.20(0.15,0.27) | 0.36(-0.02,0.74) |
| Federal Republic of Nigeria | 2.54(1.36,4.45) | 0.05(0.03,0.09) | 12.59(8.30,16.86) | 0.14(0.10,0.19) | 3.67(3.47,3.88) |
| Federal Republic of Somalia | 0.02(0.01,0.05) | 0.01(0.00,0.02) | 0.06(0.02,0.21) | 0.01(0.00,0.03) | 0.73(0.68,0.78) |
| Federated States of Micronesia | 0.00(0.00,0.00) | 0.02(0.01,0.05) | 0.00(0.00,0.00) | 0.03(0.01,0.05) | 0.17(0.03,0.32) |
| Federative Republic of Brazil | 8.60(7.53,9.64) | 0.09(0.07,0.10) | 52.03(44.70,59.52) | 0.17(0.14,0.19) | 1.90(1.41,2.40) |
| French Republic | 15.80(12.26,20.11) | 0.14(0.11,0.18) | 30.77(21.25,43.12) | 0.16(0.11,0.23) | 0.34(0.11,0.57) |
| Gabonese Republic | 0.04(0.02,0.07) | 0.06(0.02,0.11) | 0.13(0.07,0.21) | 0.12(0.07,0.20) | 2.19(2.07,2.31) |
| Georgia | 0.16(0.10,0.24) | 0.02(0.01,0.03) | 3.20(2.09,4.61) | 0.41(0.27,0.58) | 13.34(11.89,14.80) |
| Grand Duchy of Luxembourg | 0.10(0.08,0.12) | 0.14(0.12,0.16) | 0.19(0.15,0.23) | 0.14(0.11,0.16) | 0.14(-0.17,0.46) |
| Greenland | 0.00(0.00,0.01) | 0.10(0.06,0.15) | 0.01(0.00,0.02) | 0.12(0.04,0.21) | 0.73(0.57,0.88) |
| Grenada | 0.00(0.00,0.01) | 0.05(0.03,0.07) | 0.02(0.01,0.02) | 0.14(0.09,0.19) | 3.58(3.26,3.91) |
| Guam | 0.00(0.00,0.00) | 0.03(0.02,0.04) | 0.01(0.01,0.01) | 0.03(0.02,0.05) | 0.94(-0.13,2.02) |
| Hashemite Kingdom of Jordan | 0.09(0.05,0.16) | 0.07(0.04,0.12) | 0.82(0.47,1.35) | 0.11(0.06,0.18) | 1.57(1.37,1.77) |
| Hellenic Republic | 1.44(1.23,1.69) | 0.07(0.06,0.09) | 3.15(2.61,3.72) | 0.10(0.08,0.11) | 0.84(0.55,1.14) |
| Hungary | 2.98(2.23,3.84) | 0.15(0.11,0.20) | 8.05(5.41,11.69) | 0.31(0.21,0.44) | 1.96(1.39,2.53) |
| Independent State of Papua New Guinea | 0.02(0.00,0.06) | 0.01(0.00,0.03) | 0.06(0.02,0.17) | 0.01(0.00,0.03) | 0.84(0.75,0.92) |
| Independent State of Samoa | 0.01(0.00,0.02) | 0.07(0.03,0.28) | 0.02(0.01,0.06) | 0.10(0.04,0.39) | 1.09(1.05,1.12) |
| Ireland | 1.00(0.77,1.26) | 0.18(0.14,0.23) | 1.66(1.20,2.20) | 0.16(0.12,0.21) | -0.93(-1.35,-0.50) |
| Islamic Republic of Afghanistan | 0.02(0.00,0.14) | 0.00(0.00,0.02) | 0.09(0.03,0.32) | 0.01(0.00,0.04) | 5.09(4.79,5.40) |
| Islamic Republic of Iran | 0.33(0.07,0.65) | 0.01(0.00,0.02) | 4.60(0.74,6.72) | 0.05(0.01,0.08) | 5.59(4.98,6.20) |
| Islamic Republic of Mauritania | 0.01(0.01,0.02) | 0.01(0.01,0.02) | 0.04(0.02,0.07) | 0.02(0.01,0.03) | 1.23(1.01,1.45) |
| Islamic Republic of Pakistan | 1.92(1.16,3.01) | 0.03(0.02,0.05) | 9.11(5.80,13.60) | 0.07(0.05,0.11) | 2.63(2.48,2.78) |
| Jamaica | 0.17(0.12,0.22) | 0.07(0.05,0.10) | 0.83(0.55,1.21) | 0.21(0.14,0.31) | 3.35(2.60,4.10) |
| Japan | 17.34(15.95,18.38) | 0.08(0.07,0.09) | 68.96(58.77,75.98) | 0.14(0.13,0.16) | 1.29(0.74,1.84) |
| Kingdom of Bahrain | 0.01(0.00,0.01) | 0.05(0.03,0.09) | 0.14(0.07,0.23) | 0.19(0.10,0.31) | 5.07(4.42,5.72) |
| Kingdom of Belgium | 2.85(2.11,3.81) | 0.14(0.10,0.18) | 5.11(3.67,6.84) | 0.16(0.12,0.22) | 0.48(0.05,0.91) |
| Kingdom of Bhutan | 0.00(0.00,0.01) | 0.01(0.00,0.02) | 0.02(0.01,0.04) | 0.03(0.01,0.06) | 4.13(4.01,4.24) |
| Kingdom of Cambodia | 0.12(0.05,0.26) | 0.02(0.01,0.05) | 0.81(0.42,1.49) | 0.06(0.03,0.11) | 3.26(3.16,3.36) |
| Kingdom of Denmark | 1.20(0.91,1.55) | 0.11(0.08,0.14) | 4.14(3.02,5.56) | 0.26(0.19,0.35) | 2.05(1.56,2.53) |
| Kingdom of Eswatini | 0.02(0.01,0.03) | 0.06(0.03,0.11) | 0.08(0.05,0.13) | 0.14(0.08,0.23) | 3.02(2.66,3.39) |
| Kingdom of Lesotho | 0.04(0.02,0.08) | 0.04(0.02,0.08) | 0.12(0.07,0.20) | 0.10(0.06,0.17) | 3.75(3.49,4.02) |
| Kingdom of Morocco | 0.68(0.35,1.30) | 0.04(0.02,0.08) | 4.51(2.48,7.73) | 0.11(0.06,0.19) | 3.40(3.22,3.57) |
| Kingdom of Norway | 1.71(1.52,1.90) | 0.18(0.16,0.20) | 2.80(2.33,3.27) | 0.21(0.17,0.24) | -0.54(-0.99,-0.08) |
| Kingdom of Saudi Arabia | 0.24(0.07,0.43) | 0.04(0.01,0.07) | 1.46(0.65,2.40) | 0.09(0.04,0.15) | 2.90(1.96,3.85) |
| Kingdom of Spain | 11.40(8.78,14.42) | 0.16(0.12,0.20) | 23.19(16.44,31.40) | 0.18(0.13,0.24) | 0.37(0.06,0.68) |
| Kingdom of Sweden | 2.28(1.74,2.94) | 0.11(0.09,0.14) | 5.10(3.65,6.89) | 0.17(0.12,0.23) | 1.16(-0.01,2.34) |
| Kingdom of Thailand | 2.93(1.78,4.63) | 0.08(0.05,0.12) | 20.41(12.56,32.77) | 0.15(0.09,0.24) | 1.69(1.53,1.84) |
| Kingdom of the Netherlands | 6.27(4.80,7.93) | 0.24(0.18,0.30) | 11.87(8.65,15.80) | 0.25(0.18,0.33) | -0.28(-0.67,0.10) |
| Kingdom of Tonga | 0.00(0.00,0.00) | 0.02(0.01,0.05) | 0.00(0.00,0.01) | 0.03(0.01,0.05) | 0.83(0.61,1.05) |
| Kyrgyz Republic | 0.08(0.05,0.11) | 0.02(0.01,0.03) | 0.67(0.47,0.92) | 0.13(0.09,0.17) | 8.48(7.23,9.74) |
| Lao People's Democratic Republic | 0.05(0.02,0.10) | 0.02(0.01,0.04) | 0.26(0.13,0.47) | 0.05(0.03,0.10) | 3.55(3.45,3.65) |
| Lebanese Republic | 0.11(0.06,0.20) | 0.05(0.03,0.08) | 0.68(0.39,1.09) | 0.09(0.05,0.14) | 2.91(2.64,3.19) |
| Malaysia | 1.14(0.63,2.01) | 0.11(0.06,0.20) | 8.29(5.14,12.59) | 0.24(0.15,0.37) | 2.33(2.06,2.61) |
| Mongolia | 0.10(0.05,0.17) | 0.08(0.04,0.15) | 0.44(0.27,0.68) | 0.19(0.12,0.29) | 2.69(2.56,2.81) |
| Montenegro | 0.06(0.04,0.09) | 0.09(0.05,0.12) | 0.20(0.14,0.28) | 0.16(0.11,0.22) | 2.38(2.15,2.61) |
| New Zealand | 1.05(0.81,1.34) | 0.20(0.16,0.26) | 2.32(1.76,3.02) | 0.21(0.16,0.27) | 0.17(-0.46,0.81) |
| North Macedonia | 0.15(0.11,0.20) | 0.07(0.05,0.09) | 0.58(0.41,0.79) | 0.14(0.10,0.19) | 2.44(2.01,2.87) |
| Northern Mariana Islands | 0.00(0.00,0.00) | 0.01(0.00,0.02) | 0.00(0.00,0.00) | 0.01(0.01,0.02) | 1.49(0.50,2.49) |
| Palestine | 0.06(0.02,0.11) | 0.06(0.03,0.12) | 0.33(0.20,0.50) | 0.13(0.08,0.19) | 3.00(2.81,3.18) |
| People's Democratic Republic of Algeria | 0.41(0.22,0.73) | 0.03(0.02,0.06) | 2.23(1.25,3.91) | 0.06(0.03,0.10) | 1.90(1.84,1.96) |
| People's Republic of Bangladesh | 0.70(0.25,1.57) | 0.01(0.00,0.03) | 5.32(2.43,9.86) | 0.03(0.02,0.06) | 2.58(2.36,2.80) |
| People's Republic of China | 48.18(32.74,71.96) | 0.05(0.04,0.08) | 518.98(354.83,656.37) | 0.20(0.14,0.25) | 5.00(4.68,5.33) |
| Plurinational State of Bolivia | 0.29(0.17,0.46) | 0.08(0.05,0.13) | 1.92(1.11,3.20) | 0.18(0.11,0.30) | 2.68(2.57,2.80) |
| Portuguese Republic | 2.63(2.00,3.37) | 0.15(0.11,0.19) | 4.93(3.49,6.67) | 0.14(0.10,0.20) | -0.17(-0.49,0.14) |
| Principality of Andorra | 0.01(0.01,0.02) | 0.14(0.07,0.24) | 0.03(0.02,0.05) | 0.15(0.08,0.26) | 0.73(0.49,0.96) |
| Principality of Monaco | 0.00(0.00,0.00) | 0.00(0.00,0.00) | 0.00(0.00,0.00) | 0.00(0.00,0.01) | 0.66(0.59,0.73) |
| Puerto Rico | 0.36(0.26,0.49) | 0.08(0.06,0.11) | 1.27(0.88,1.78) | 0.13(0.09,0.18) | 1.71(1.27,2.15) |
| Republic of Albania | 0.05(0.03,0.07) | 0.02(0.01,0.03) | 0.22(0.13,0.36) | 0.04(0.02,0.06) | 2.45(2.23,2.66) |
| Republic of Angola | 0.08(0.03,0.18) | 0.02(0.01,0.05) | 0.55(0.22,1.15) | 0.05(0.02,0.10) | 2.70(2.50,2.91) |
| Republic of Armenia | 0.30(0.16,0.50) | 0.09(0.05,0.15) | 2.23(1.47,3.31) | 0.39(0.25,0.57) | 5.83(5.16,6.49) |
| Republic of Austria | 2.52(1.95,3.20) | 0.16(0.12,0.20) | 3.52(2.58,4.58) | 0.15(0.11,0.19) | -0.14(-0.50,0.22) |
| Republic of Azerbaijan | 0.53(0.27,0.99) | 0.10(0.05,0.18) | 1.44(0.82,2.39) | 0.13(0.07,0.22) | 1.80(1.39,2.23) |
| Republic of Belarus | 1.92(1.32,2.69) | 0.12(0.08,0.16) | 8.93(5.89,12.95) | 0.41(0.27,0.60) | 3.66(3.20,4.12) |
| Republic of Benin | 0.01(0.01,0.03) | 0.01(0.00,0.01) | 0.05(0.03,0.09) | 0.01(0.01,0.02) | 1.34(1.21,1.46) |
| Republic of Botswana | 0.04(0.02,0.07) | 0.06(0.03,0.12) | 0.22(0.12,0.37) | 0.15(0.08,0.24) | 2.88(2.59,3.16) |
| Republic of Bulgaria | 0.93(0.60,1.32) | 0.06(0.04,0.09) | 2.59(1.72,3.69) | 0.13(0.09,0.19) | 1.49(0.83,2.16) |
| Republic of Burundi | 0.04(0.02,0.08) | 0.02(0.01,0.03) | 0.10(0.04,0.23) | 0.02(0.01,0.05) | 0.75(0.56,0.94) |
| Republic of Cabo Verde | 0.00(0.00,0.00) | 0.00(0.00,0.00) | 0.00(0.00,0.01) | 0.01(0.00,0.01) | 4.52(4.31,4.74) |
| Republic of Cameroon | 0.05(0.03,0.08) | 0.01(0.01,0.02) | 0.20(0.11,0.33) | 0.02(0.01,0.03) | 1.28(1.15,1.40) |
| Republic of Chad | 0.01(0.00,0.02) | 0.00(0.00,0.01) | 0.03(0.02,0.07) | 0.01(0.00,0.01) | 1.84(1.74,1.93) |
| Republic of Chile | 0.53(0.40,0.69) | 0.04(0.03,0.06) | 4.99(3.60,6.66) | 0.15(0.11,0.20) | 4.25(3.10,5.41) |
| Republic of Colombia | 1.51(1.13,1.95) | 0.08(0.06,0.10) | 10.48(7.28,14.37) | 0.15(0.11,0.21) | 1.43(0.73,2.14) |
| Republic of Costa Rica | 0.18(0.13,0.23) | 0.09(0.06,0.11) | 1.32(0.94,1.77) | 0.19(0.14,0.26) | 1.78(1.23,2.34) |
| Republic of Croatia | 1.65(1.22,2.16) | 0.22(0.17,0.29) | 5.65(3.94,7.88) | 0.46(0.32,0.64) | 1.97(1.61,2.33) |
| Republic of Cuba | 1.31(0.96,1.74) | 0.10(0.08,0.14) | 3.67(2.65,4.96) | 0.15(0.11,0.20) | 2.21(1.68,2.73) |
| Republic of Cyprus | 0.21(0.10,0.37) | 0.23(0.12,0.41) | 0.67(0.42,1.03) | 0.25(0.15,0.38) | 0.06(-0.19,0.31) |
| The Republic of Côte d'Ivoire | 0.02(0.01,0.04) | 0.01(0.00,0.01) | 0.10(0.05,0.18) | 0.01(0.00,0.02) | 1.28(1.13,1.43) |
| Republic of Djibouti | 0.00(0.00,0.01) | 0.03(0.01,0.05) | 0.04(0.02,0.07) | 0.06(0.03,0.10) | 2.72(2.68,2.76) |
| Republic of Ecuador | 0.46(0.33,0.61) | 0.08(0.06,0.10) | 4.54(3.11,6.51) | 0.23(0.16,0.33) | 4.19(3.63,4.75) |
| Republic of El Salvador | 0.12(0.07,0.17) | 0.03(0.02,0.05) | 0.51(0.35,0.73) | 0.07(0.05,0.10) | 2.05(1.86,2.25) |
| Republic of Equatorial Guinea | 0.00(0.00,0.01) | 0.02(0.01,0.05) | 0.06(0.03,0.10) | 0.12(0.06,0.20) | 5.88(5.73,6.03) |
| Republic of Estonia | 0.38(0.26,0.53) | 0.14(0.10,0.20) | 1.82(1.22,2.59) | 0.51(0.34,0.72) | 2.97(2.45,3.50) |
| Republic of Fiji | 0.04(0.02,0.07) | 0.11(0.05,0.22) | 0.08(0.04,0.21) | 0.10(0.04,0.25) | -1.41(-2.00,-0.82) |
| Republic of Finland | 1.07(0.78,1.41) | 0.11(0.08,0.15) | 4.61(3.23,6.36) | 0.26(0.19,0.36) | 2.68(2.26,3.10) |
| Republic of Ghana | 0.06(0.01,0.12) | 0.01(0.00,0.02) | 0.13(0.07,0.23) | 0.01(0.00,0.01) | -2.47(-3.61,-1.32) |
| Republic of Guatemala | 0.09(0.07,0.12) | 0.03(0.02,0.03) | 0.38(0.30,0.46) | 0.03(0.02,0.04) | -0.07(-0.33,0.20) |
| Republic of Guinea | 0.03(0.01,0.05) | 0.01(0.00,0.01) | 0.08(0.04,0.15) | 0.01(0.01,0.03) | 2.06(1.99,2.13) |
| Republic of Guinea-Bissau | 0.00(0.00,0.01) | 0.01(0.00,0.01) | 0.01(0.00,0.01) | 0.01(0.01,0.02) | 1.25(1.15,1.35) |
| Republic of Guyana | 0.00(0.00,0.00) | 0.00(0.00,0.01) | 0.05(0.03,0.08) | 0.07(0.05,0.10) | 7.59(5.36,9.88) |
| Republic of Haiti | 0.08(0.04,0.17) | 0.02(0.01,0.05) | 0.32(0.15,0.61) | 0.04(0.02,0.08) | 2.15(1.96,2.34) |
| Republic of Honduras | 0.09(0.04,0.19) | 0.04(0.02,0.08) | 1.04(0.61,1.69) | 0.14(0.08,0.23) | 4.44(4.13,4.75) |
| Republic of Iceland | 0.06(0.04,0.07) | 0.16(0.12,0.20) | 0.18(0.13,0.24) | 0.23(0.16,0.31) | 1.47(1.23,1.72) |
| Republic of India | 10.84(6.54,16.17) | 0.02(0.01,0.03) | 76.84(62.87,94.48) | 0.06(0.04,0.07) | 2.57(2.26,2.89) |
| Republic of Indonesia | 3.72(2.38,5.25) | 0.04(0.02,0.05) | 28.21(19.78,37.43) | 0.11(0.08,0.14) | 3.48(3.33,3.62) |
| Republic of Iraq | 0.20(0.09,0.39) | 0.02(0.01,0.04) | 1.62(0.91,2.66) | 0.07(0.04,0.11) | 3.52(3.33,3.70) |
| Republic of Italy | 15.36(13.57,16.93) | 0.13(0.11,0.14) | 35.65(29.20,41.37) | 0.18(0.15,0.21) | 1.32(0.99,1.65) |
| Republic of Kazakhstan | 0.92(0.52,1.36) | 0.06(0.03,0.09) | 2.45(1.50,3.73) | 0.12(0.07,0.18) | 1.81(1.59,2.02) |
| Republic of Kenya | 0.10(0.04,0.20) | 0.01(0.00,0.02) | 0.85(0.54,1.25) | 0.04(0.02,0.05) | 3.79(3.70,3.89) |
| Republic of Kiribati | 0.00(0.00,0.00) | 0.00(0.00,0.01) | 0.00(0.00,0.00) | 0.01(0.00,0.01) | 1.30(1.22,1.37) |
| Republic of Korea | 3.52(2.12,5.42) | 0.11(0.07,0.17) | 11.97(7.53,18.10) | 0.10(0.06,0.15) | -0.97(-1.23,-0.70) |
| Republic of Latvia | 0.47(0.31,0.69) | 0.10(0.07,0.15) | 1.22(0.79,1.83) | 0.23(0.15,0.35) | 3.26(2.97,3.55) |
| Republic of Liberia | 0.01(0.00,0.02) | 0.01(0.00,0.01) | 0.02(0.01,0.03) | 0.01(0.00,0.02) | 0.89(0.63,1.15) |
| Republic of Lithuania | 0.53(0.35,0.75) | 0.09(0.06,0.13) | 2.88(1.93,4.10) | 0.38(0.25,0.54) | 4.86(4.60,5.12) |
| Republic of Madagascar | 0.10(0.05,0.18) | 0.02(0.01,0.03) | 0.32(0.17,0.57) | 0.03(0.02,0.05) | 1.58(1.26,1.91) |
| Republic of Malawi | 0.16(0.08,0.28) | 0.04(0.02,0.07) | 0.65(0.37,1.03) | 0.08(0.05,0.13) | 2.53(2.42,2.65) |
| Republic of Maldives | 0.01(0.00,0.02) | 0.12(0.05,0.26) | 0.10(0.06,0.16) | 0.30(0.18,0.48) | 2.69(2.22,3.16) |
| Republic of Mali | 0.02(0.01,0.03) | 0.00(0.00,0.01) | 0.06(0.03,0.12) | 0.01(0.00,0.01) | 1.98(1.87,2.09) |
| Republic of Malta | 0.16(0.12,0.20) | 0.29(0.22,0.37) | 0.43(0.31,0.57) | 0.31(0.23,0.42) | 0.14(-0.23,0.51) |
| Republic of Mauritius | 0.06(0.05,0.07) | 0.07(0.05,0.08) | 0.44(0.37,0.52) | 0.19(0.15,0.22) | 3.32(2.35,4.29) |
| Republic of Moldova | 0.33(0.24,0.46) | 0.06(0.04,0.08) | 1.38(1.12,1.68) | 0.17(0.14,0.21) | 4.02(3.49,4.56) |
| Republic of Mozambique | 0.08(0.03,0.19) | 0.01(0.00,0.03) | 0.35(0.16,0.74) | 0.03(0.01,0.07) | 3.25(3.05,3.44) |
| Republic of Namibia | 0.03(0.02,0.06) | 0.04(0.02,0.08) | 0.12(0.07,0.24) | 0.08(0.04,0.17) | 2.04(1.84,2.25) |
| Republic of Nauru | 0.00(0.00,0.00) | 0.04(0.02,0.09) | 0.00(0.00,0.00) | 0.04(0.02,0.08) | 0.18(-0.10,0.45) |
| Republic of Nicaragua | 0.14(0.08,0.21) | 0.08(0.05,0.13) | 0.81(0.58,1.12) | 0.14(0.10,0.20) | 1.85(1.51,2.20) |
| Republic of Niue | 0.00(0.00,0.00) | 0.03(0.02,0.07) | 0.00(0.00,0.00) | 0.05(0.02,0.08) | 0.80(0.60,0.99) |
| Republic of Palau | 0.00(0.00,0.00) | 0.01(0.01,0.02) | 0.00(0.00,0.00) | 0.01(0.01,0.02) | 0.09(0.02,0.16) |
| Republic of Panama | 0.19(0.16,0.23) | 0.11(0.09,0.13) | 0.76(0.56,0.97) | 0.14(0.10,0.18) | 0.53(0.32,0.74) |
| Republic of Paraguay | 0.10(0.05,0.17) | 0.04(0.02,0.06) | 0.59(0.34,0.92) | 0.09(0.05,0.13) | 2.72(2.60,2.84) |
| Republic of Peru | 1.15(0.72,1.78) | 0.09(0.05,0.13) | 6.26(3.78,9.99) | 0.16(0.09,0.25) | 2.04(1.92,2.16) |
| Republic of Poland | 5.68(5.18,6.18) | 0.10(0.09,0.11) | 18.88(16.63,21.04) | 0.19(0.17,0.21) | 1.44(0.38,2.51) |
| Republic of Rwanda | 0.08(0.04,0.13) | 0.02(0.01,0.04) | 0.29(0.15,0.51) | 0.04(0.02,0.07) | 1.65(1.33,1.98) |
| Republic of San Marino | 0.00(0.00,0.00) | 0.04(0.02,0.06) | 0.00(0.00,0.00) | 0.03(0.01,0.05) | -0.32(-0.76,0.12) |
| Republic of Senegal | 0.03(0.01,0.04) | 0.01(0.00,0.01) | 0.11(0.06,0.19) | 0.01(0.01,0.02) | 2.02(1.84,2.21) |
| Republic of Serbia | 2.70(1.52,4.47) | 0.21(0.12,0.35) | 6.46(4.07,9.92) | 0.29(0.18,0.44) | 0.91(0.79,1.02) |
| Republic of Seychelles | 0.00(0.00,0.00) | 0.01(0.00,0.03) | 0.00(0.00,0.00) | 0.02(0.00,0.04) | 1.41(1.23,1.60) |
| Republic of Sierra Leone | 0.01(0.01,0.02) | 0.01(0.00,0.01) | 0.03(0.02,0.06) | 0.01(0.00,0.02) | 1.69(1.45,1.94) |
| Republic of Singapore | 0.34(0.26,0.44) | 0.13(0.10,0.17) | 1.33(0.93,1.84) | 0.12(0.09,0.17) | -0.54(-1.02,-0.06) |
| Republic of Slovenia | 0.34(0.26,0.45) | 0.11(0.08,0.14) | 0.88(0.62,1.24) | 0.15(0.10,0.21) | 0.78(0.09,1.49) |
| Republic of South Africa | 2.31(1.17,3.29) | 0.10(0.05,0.14) | 9.41(6.08,11.50) | 0.18(0.11,0.22) | 1.82(1.57,2.07) |
| Republic of South Sudan | 0.03(0.01,0.09) | 0.01(0.00,0.03) | 0.08(0.03,0.18) | 0.02(0.01,0.05) | 1.97(1.86,2.08) |
| Republic of Sudan | 0.03(0.01,0.17) | 0.00(0.00,0.02) | 0.27(0.12,0.65) | 0.01(0.01,0.03) | 5.68(5.32,6.04) |
| Republic of Suriname | 0.01(0.00,0.01) | 0.02(0.01,0.05) | 0.03(0.02,0.05) | 0.04(0.02,0.07) | 2.55(2.16,2.94) |
| Republic of Tajikistan | 0.01(0.00,0.02) | 0.00(0.00,0.01) | 0.02(0.01,0.03) | 0.00(0.00,0.01) | 0.12(-0.17,0.41) |
| Republic of the Congo | 0.06(0.03,0.10) | 0.05(0.03,0.09) | 0.22(0.12,0.36) | 0.09(0.05,0.14) | 1.50(1.32,1.68) |
| Republic of the Gambia | 0.00(0.00,0.01) | 0.01(0.01,0.02) | 0.02(0.01,0.05) | 0.02(0.01,0.04) | 2.13(1.94,2.32) |
| Republic of the Marshall Islands | 0.00(0.00,0.00) | 0.02(0.01,0.05) | 0.00(0.00,0.00) | 0.03(0.01,0.06) | 1.12(1.05,1.20) |
| Republic of the Niger | 0.01(0.00,0.02) | 0.00(0.00,0.01) | 0.03(0.01,0.08) | 0.00(0.00,0.01) | 0.78(0.67,0.89) |
| Republic of the Philippines | 2.18(1.21,2.93) | 0.07(0.04,0.10) | 13.00(8.99,16.21) | 0.14(0.10,0.17) | 2.21(2.14,2.29) |
| Republic of the Union of Myanmar | 0.62(0.32,1.22) | 0.02(0.01,0.05) | 3.41(1.92,5.66) | 0.06(0.03,0.10) | 3.35(3.27,3.43) |
| Republic of Trinidad and Tobago | 0.16(0.13,0.20) | 0.16(0.13,0.20) | 0.71(0.52,0.94) | 0.29(0.21,0.38) | 2.15(2.01,2.29) |
| Republic of Tunisia | 0.34(0.17,0.62) | 0.06(0.03,0.11) | 2.17(1.14,3.71) | 0.14(0.07,0.23) | 2.54(2.37,2.72) |
| Republic of Turkey | 3.34(1.63,6.11) | 0.09(0.04,0.16) | 27.13(16.73,41.46) | 0.24(0.15,0.37) | 3.54(3.16,3.93) |
| Republic of Uganda | 0.08(0.05,0.14) | 0.01(0.01,0.02) | 0.47(0.26,0.79) | 0.03(0.02,0.05) | 3.04(2.90,3.17) |
| Republic of Uzbekistan | 1.75(0.61,3.10) | 0.13(0.04,0.23) | 6.86(4.34,9.96) | 0.23(0.15,0.34) | 2.19(1.99,2.40) |
| Republic of Vanuatu | 0.00(0.00,0.00) | 0.01(0.00,0.03) | 0.00(0.00,0.01) | 0.02(0.01,0.04) | 0.85(0.75,0.94) |
| Republic of Yemen | 0.01(0.00,0.06) | 0.00(0.00,0.01) | 0.16(0.06,0.43) | 0.01(0.00,0.03) | 6.33(5.95,6.71) |
| Republic of Zambia | 0.07(0.04,0.12) | 0.02(0.01,0.04) | 0.56(0.26,1.08) | 0.08(0.04,0.15) | 4.47(3.98,4.96) |
| Republic of Zimbabwe | 0.17(0.10,0.27) | 0.04(0.02,0.06) | 0.41(0.22,0.70) | 0.06(0.03,0.10) | 0.52(0.10,0.95) |
| Romania | 3.64(2.33,5.44) | 0.11(0.07,0.16) | 8.21(5.60,11.58) | 0.16(0.11,0.23) | 1.31(1.13,1.48) |
| Russian Federation | 39.52(26.78,54.14) | 0.17(0.11,0.23) | 63.10(54.88,71.11) | 0.19(0.17,0.22) | -1.39(-2.28,-0.48) |
| Saint Kitts and Nevis | 0.00(0.00,0.00) | 0.04(0.03,0.07) | 0.01(0.00,0.01) | 0.09(0.05,0.15) | 3.16(2.61,3.72) |
| Saint Lucia | 0.01(0.00,0.01) | 0.06(0.03,0.08) | 0.03(0.02,0.04) | 0.10(0.06,0.15) | 1.60(1.49,1.72) |
| Saint Vincent and the Grenadines | 0.00(0.00,0.00) | 0.00(0.00,0.00) | 0.02(0.01,0.02) | 0.10(0.07,0.12) | 9.34(5.77,13.04) |
| Slovak Republic | 0.99(0.63,1.46) | 0.13(0.08,0.19) | 2.46(1.49,3.77) | 0.19(0.12,0.30) | 1.32(1.00,1.64) |
| Socialist Republic of Viet Nam | 2.05(1.20,3.49) | 0.04(0.02,0.07) | 11.66(6.51,20.97) | 0.10(0.06,0.18) | 2.74(2.56,2.92) |
| Solomon Islands | 0.00(0.00,0.00) | 0.01(0.00,0.03) | 0.01(0.00,0.01) | 0.01(0.01,0.04) | 0.75(0.57,0.94) |
| State of Eritrea | 0.02(0.01,0.03) | 0.02(0.01,0.03) | 0.12(0.06,0.20) | 0.04(0.02,0.08) | 3.04(2.76,3.32) |
| State of Israel | 0.95(0.68,1.29) | 0.15(0.11,0.20) | 2.67(1.89,3.62) | 0.16(0.12,0.22) | 0.04(-0.36,0.44) |
| State of Kuwait | 0.03(0.02,0.04) | 0.05(0.04,0.07) | 0.24(0.16,0.35) | 0.09(0.06,0.13) | 4.42(2.69,6.17) |
| State of Libya | 0.13(0.05,0.28) | 0.06(0.02,0.13) | 0.76(0.40,1.33) | 0.14(0.07,0.25) | 2.90(2.37,3.42) |
| State of Qatar | 0.00(0.00,0.00) | 0.02(0.01,0.04) | 0.02(0.01,0.04) | 0.04(0.02,0.07) | 3.27(2.58,3.95) |
| Sultanate of Oman | 0.02(0.01,0.05) | 0.04(0.01,0.08) | 0.15(0.08,0.25) | 0.08(0.05,0.14) | 2.50(2.08,2.93) |
| Swiss Confederation | 2.33(1.76,2.99) | 0.17(0.13,0.22) | 6.91(4.83,9.40) | 0.28(0.20,0.39) | 1.10(0.64,1.57) |
| Syrian Arab Republic | 0.07(0.02,0.15) | 0.01(0.00,0.03) | 0.41(0.20,0.73) | 0.03(0.01,0.05) | 2.78(2.45,3.10) |
| Taiwan (Province of China) | 1.93(1.49,2.45) | 0.10(0.08,0.13) | 10.95(7.77,15.12) | 0.20(0.14,0.27) | 1.52(1.12,1.92) |
| Togolese Republic | 0.01(0.01,0.02) | 0.01(0.00,0.01) | 0.04(0.02,0.07) | 0.01(0.01,0.02) | 1.05(0.90,1.19) |
| Tokelau | 0.00(0.00,0.00) | 0.03(0.01,0.06) | 0.00(0.00,0.00) | 0.04(0.02,0.07) | 0.67(0.44,0.90) |
| Turkmenistan | 0.21(0.12,0.32) | 0.09(0.05,0.15) | 0.84(0.47,1.38) | 0.19(0.11,0.31) | 2.42(2.17,2.68) |
| Tuvalu | 0.00(0.00,0.00) | 0.02(0.01,0.04) | 0.00(0.00,0.00) | 0.03(0.01,0.05) | 0.84(0.54,1.15) |
| Ukraine | 14.23(8.27,21.77) | 0.15(0.08,0.23) | 21.10(13.09,31.58) | 0.20(0.12,0.30) | 1.00(0.82,1.19) |
| Union of the Comoros | 0.00(0.00,0.01) | 0.02(0.01,0.04) | 0.03(0.02,0.05) | 0.05(0.03,0.09) | 3.15(3.01,3.30) |
| United Arab Emirates | 0.02(0.01,0.05) | 0.07(0.03,0.15) | 0.33(0.19,0.54) | 0.16(0.10,0.27) | 4.14(3.41,4.86) |
| United Kingdom of Great Britain and Northern Ireland | 26.26(24.46,27.64) | 0.21(0.20,0.23) | 32.73(29.28,35.05) | 0.18(0.17,0.20) | -0.48(-0.81,-0.16) |
| United Mexican States | 2.81(2.61,3.00) | 0.06(0.06,0.06) | 22.41(19.31,25.59) | 0.15(0.13,0.17) | 2.41(1.50,3.33) |
| United Republic of Tanzania | 0.23(0.10,0.45) | 0.02(0.01,0.04) | 1.12(0.60,1.95) | 0.04(0.02,0.07) | 2.66(2.50,2.82) |
| United States of America | 62.38(55.46,68.25) | 0.15(0.13,0.16) | 123.81(107.87,136.52) | 0.16(0.14,0.17) | 0.04(-0.33,0.42) |
| United States Virgin Islands | 0.00(0.00,0.01) | 0.04(0.02,0.07) | 0.01(0.01,0.02) | 0.05(0.03,0.10) | 1.11(0.55,1.68) |

EAPC: Estimated Annual Percentage Change
