# Supplementary material for: Global, regional, and national burden of neuroblastoma and peripheral nervous system tumours in individuals aged over 60 from 1990 to 2021: a trend analysis of global burden of disease study
Source: J Health Popul Nutr. 2025 Mar 17;44:78. doi: 10.1186/s41043-025-00810-9 (PMC11916991; doi:10.1186/s41043-025-00810-9)
Supplement: Supplementary file 14 — Supplementary Material 14 [file 41043_2025_810_MOESM14_ESM.docx]

Supplement 7. The age-standardized DALYs rate, number of DALYs, and EAPC of neuroblastoma and peripheral nervous system tumours among individuals aged 60 and above across 204 countries from 1990 to 2021

| Nation | DALYs (95% UI) | | | | |
| --- | --- | --- | --- | --- | --- |
|  | Cases in 1990 (million) | Age-standardised rate in 1990 (per 100 000) | Cases in 2021(million) | Age-standardised rate in 2021 (per 100 000) | EAPC (95% CI) |
|  |  |  |  |  |  |
| American Samoa | 0.06(0.02,0.11) | 2.01(0.82,3.78) | 0.10(0.04,0.20) | 1.52(0.70,3.07) | -1.47(-2.41,-0.51) |
| Antigua and Barbuda | 0.07(0.05,0.10) | 1.06(0.68,1.58) | 0.23(0.15,0.35) | 1.70(1.12,2.57) | 1.66(1.38,1.94) |
| Arab Republic of Egypt | 18.68(8.39,37.62) | 0.62(0.28,1.26) | 89.52(50.99,168.32) | 1.22(0.70,2.31) | 1.79(1.42,2.16) |
| Argentine Republic | 111.95(79.55,149.46) | 2.67(1.90,3.57) | 270.34(196.32,360.85) | 3.79(2.75,5.06) | 1.53(1.25,1.81) |
| Australia | 85.97(66.37,107.65) | 3.33(2.57,4.17) | 196.40(141.38,262.15) | 3.35(2.42,4.46) | -0.35(-0.59,-0.10) |
| Barbados | 1.70(1.41,2.02) | 4.61(3.84,5.46) | 4.61(3.41,6.03) | 6.67(4.94,8.72) | 1.69(1.50,1.87) |
| Belize | 0.12(0.08,0.16) | 1.09(0.71,1.44) | 0.89(0.70,1.08) | 2.48(1.97,3.04) | 2.71(1.85,3.58) |
| Bermuda | 0.09(0.07,0.13) | 1.21(0.89,1.65) | 0.24(0.17,0.33) | 1.31(0.93,1.83) | 0.44(-0.01,0.89) |
| Bolivarian Republic of Venezuela | 31.33(25.87,37.96) | 2.78(2.29,3.37) | 145.09(104.19,194.74) | 3.81(2.74,5.10) | 0.95(0.48,1.43) |
| Bosnia and Herzegovina | 7.42(4.09,13.26) | 1.53(0.84,2.76) | 24.34(14.69,37.16) | 2.88(1.74,4.39) | 2.62(2.33,2.91) |
| Brunei Darussalam | 0.24(0.13,0.42) | 2.19(1.16,3.82) | 0.87(0.56,1.31) | 2.11(1.35,3.18) | -0.06(-0.44,0.33) |
| Burkina Faso | 0.46(0.19,0.98) | 0.09(0.04,0.19) | 1.39(0.68,2.67) | 0.14(0.07,0.27) | 1.34(1.16,1.51) |
| Canada | 140.85(107.59,180.43) | 3.33(2.55,4.27) | 284.05(209.15,373.15) | 2.96(2.18,3.88) | -0.41(-0.64,-0.16) |
| Central African Republic | 0.53(0.22,1.32) | 0.40(0.16,1.01) | 1.19(0.51,2.87) | 0.51(0.22,1.23) | 0.72(0.64,0.81) |
| Commonwealth of Dominica | 0.09(0.05,0.16) | 1.19(0.61,2.11) | 0.26(0.14,0.45) | 2.41(1.26,4.09) | 2.34(2.10,2.59) |
| Commonwealth of the Bahamas | 0.28(0.22,0.35) | 1.60(1.26,1.99) | 1.15(0.87,1.49) | 2.27(1.72,2.92) | 1.40(1.24,1.57) |
| Cook Islands | 0.01(0.00,0.02) | 0.58(0.25,1.14) | 0.03(0.02,0.06) | 0.90(0.45,1.57) | 1.39(1.29,1.49) |
| Czech Republic | 67.96(49.36,90.51) | 3.70(2.68,4.94) | 165.54(112.36,236.74) | 5.93(4.01,8.48) | 1.26(0.88,1.63) |
| Democratic People's Republic of Korea | 18.85(10.42,32.76) | 1.02(0.56,1.78) | 57.12(28.81,102.94) | 1.42(0.72,2.58) | 1.60(1.38,1.82) |
| Democratic Republic of Sao Tome and Principe | 0.01(0.01,0.02) | 0.16(0.08,0.27) | 0.04(0.02,0.06) | 0.32(0.18,0.52) | 2.53(2.31,2.75) |
| Democratic Republic of the Congo | 8.50(3.40,19.34) | 0.47(0.18,1.06) | 24.18(9.66,55.35) | 0.60(0.24,1.38) | 0.77(0.18,1.36) |
| Democratic Republic of Timor-Leste | 0.09(0.02,0.21) | 0.30(0.09,0.75) | 0.91(0.43,1.78) | 0.83(0.39,1.61) | 3.37(3.22,3.51) |
| Democratic Socialist Republic of Sri Lanka | 27.19(15.16,45.22) | 2.17(1.21,3.61) | 132.76(69.25,223.00) | 3.68(1.93,6.16) | 1.83(1.60,2.05) |
| Dominican Republic | 1.02(0.49,2.78) | 0.24(0.11,0.64) | 17.89(10.92,28.24) | 1.47(0.90,2.33) | 5.40(4.61,6.19) |
| Eastern Republic of Uruguay | 16.44(11.30,23.33) | 3.18(2.18,4.52) | 33.54(22.82,47.20) | 4.81(3.25,6.79) | 1.45(1.30,1.60) |
| Federal Democratic Republic of Ethiopia | 9.11(2.76,30.03) | 0.39(0.12,1.28) | 42.03(16.70,96.60) | 0.87(0.34,1.99) | 2.53(2.24,2.82) |
| Federal Democratic Republic of Nepal | 2.15(0.64,5.07) | 0.20(0.06,0.47) | 14.96(6.99,27.99) | 0.52(0.24,0.97) | 3.29(3.06,3.51) |
| Federal Republic of Germany | 556.88(428.65,703.77) | 3.44(2.64,4.36) | 983.95(729.10,1298.21) | 4.03(2.99,5.32) | 0.30(-0.09,0.68) |
| Federal Republic of Nigeria | 52.66(27.43,93.64) | 1.02(0.54,1.80) | 257.46(162.72,349.14) | 2.70(1.76,3.64) | 3.56(3.36,3.76) |
| Federal Republic of Somalia | 0.38(0.12,1.16) | 0.16(0.05,0.49) | 1.33(0.39,4.96) | 0.20(0.06,0.73) | 0.70(0.65,0.74) |
| Federated States of Micronesia | 0.03(0.01,0.06) | 0.48(0.19,1.02) | 0.05(0.02,0.10) | 0.54(0.24,1.08) | 0.04(-0.10,0.18) |
| Federative Republic of Brazil | 187.17(164.29,210.00) | 1.75(1.54,1.97) | 1093.02(947.78,1245.40) | 3.46(3.00,3.95) | 1.85(1.34,2.36) |
| French Republic | 301.21(234.79,383.46) | 2.81(2.19,3.60) | 565.57(389.38,792.40) | 3.23(2.22,4.50) | 0.38(0.16,0.60) |
| Gabonese Republic | 0.82(0.37,1.61) | 1.16(0.51,2.28) | 2.88(1.57,4.77) | 2.39(1.32,3.96) | 2.11(1.99,2.23) |
| Georgia | 3.48(2.26,5.32) | 0.42(0.27,0.65) | 66.28(43.30,95.79) | 8.38(5.48,12.09) | 13.26(11.81,14.73) |
| Grand Duchy of Luxembourg | 1.95(1.64,2.28) | 2.74(2.30,3.21) | 3.46(2.80,4.17) | 2.61(2.11,3.14) | 0.03(-0.30,0.35) |
| Greenland | 0.08(0.04,0.11) | 2.14(1.20,3.11) | 0.19(0.06,0.34) | 2.22(0.71,3.93) | 0.43(0.30,0.56) |
| Grenada | 0.09(0.06,0.14) | 0.97(0.61,1.49) | 0.34(0.23,0.49) | 2.56(1.75,3.67) | 3.23(2.92,3.54) |
| Guam | 0.05(0.03,0.08) | 0.59(0.36,0.86) | 0.17(0.12,0.25) | 0.68(0.46,0.97) | 0.76(-0.22,1.76) |
| Hashemite Kingdom of Jordan | 2.05(1.13,3.54) | 1.44(0.79,2.47) | 17.84(10.13,29.52) | 2.19(1.25,3.62) | 1.44(1.22,1.65) |
| Hellenic Republic | 28.01(23.95,32.68) | 1.40(1.20,1.63) | 55.78(46.94,65.62) | 1.89(1.60,2.22) | 0.95(0.71,1.20) |
| Hungary | 64.14(47.86,82.71) | 3.26(2.42,4.20) | 168.36(114.06,243.73) | 6.62(4.52,9.52) | 1.97(1.40,2.54) |
| Independent State of Papua New Guinea | 0.40(0.07,1.47) | 0.20(0.04,0.66) | 1.36(0.36,3.90) | 0.26(0.07,0.70) | 0.65(0.56,0.75) |
| Independent State of Samoa | 0.13(0.07,0.43) | 1.33(0.68,4.89) | 0.33(0.15,1.10) | 1.90(0.88,6.78) | 1.12(1.08,1.15) |
| Ireland | 19.87(15.47,25.06) | 3.67(2.86,4.63) | 31.06(22.58,41.44) | 3.05(2.22,4.07) | -1.05(-1.46,-0.63) |
| Islamic Republic of Afghanistan | 0.52(0.07,3.30) | 0.06(0.01,0.37) | 1.93(0.68,6.89) | 0.24(0.09,0.87) | 5.09(4.79,5.38) |
| Islamic Republic of Iran | 7.42(1.68,14.50) | 0.25(0.05,0.49) | 93.71(15.21,136.63) | 1.04(0.17,1.51) | 5.64(5.02,6.26) |
| Islamic Republic of Mauritania | 0.28(0.16,0.44) | 0.23(0.14,0.37) | 0.84(0.46,1.39) | 0.35(0.19,0.57) | 0.95(0.71,1.18) |
| Islamic Republic of Pakistan | 40.52(24.33,63.39) | 0.61(0.36,0.95) | 197.57(125.88,294.95) | 1.46(0.93,2.18) | 2.60(2.45,2.75) |
| Jamaica | 3.35(2.45,4.45) | 1.47(1.07,1.95) | 17.23(11.35,25.31) | 4.44(2.93,6.53) | 3.36(2.59,4.13) |
| Japan | 359.31(333.80,379.68) | 1.64(1.52,1.74) | 1237.55(1083.00,1351.17) | 2.94(2.63,3.19) | 1.29(0.71,1.87) |
| Kingdom of Bahrain | 0.19(0.10,0.32) | 1.05(0.58,1.80) | 3.14(1.58,5.37) | 3.50(1.79,5.85) | 4.79(4.20,5.38) |
| Kingdom of Belgium | 55.45(41.29,73.54) | 2.73(2.04,3.62) | 94.97(68.92,126.10) | 3.23(2.35,4.27) | 0.49(0.07,0.92) |
| Kingdom of Bhutan | 0.06(0.01,0.14) | 0.21(0.05,0.49) | 0.45(0.18,0.87) | 0.62(0.24,1.20) | 3.94(3.83,4.05) |
| Kingdom of Cambodia | 2.65(1.18,5.93) | 0.50(0.22,1.12) | 18.12(9.22,33.45) | 1.21(0.62,2.22) | 3.11(3.02,3.21) |
| Kingdom of Denmark | 23.53(17.88,30.34) | 2.25(1.71,2.90) | 76.93(56.25,103.28) | 5.05(3.68,6.79) | 1.88(1.40,2.37) |
| Kingdom of Eswatini | 0.38(0.18,0.71) | 1.19(0.58,2.25) | 1.81(1.03,2.91) | 2.84(1.61,4.57) | 2.98(2.60,3.35) |
| Kingdom of Lesotho | 0.75(0.34,1.63) | 0.74(0.33,1.60) | 2.65(1.51,4.41) | 2.02(1.14,3.37) | 3.77(3.51,4.04) |
| Kingdom of Morocco | 14.63(7.37,28.35) | 0.85(0.43,1.64) | 98.05(54.40,168.72) | 2.29(1.27,3.94) | 3.40(3.22,3.57) |
| Kingdom of Norway | 32.61(29.18,36.01) | 3.61(3.24,3.99) | 51.13(42.85,59.54) | 3.95(3.32,4.60) | -0.64(-1.09,-0.19) |
| Kingdom of Saudi Arabia | 5.20(1.48,9.37) | 0.83(0.24,1.50) | 34.14(15.39,56.21) | 1.88(0.84,3.08) | 2.93(1.98,3.89) |
| Kingdom of Spain | 223.82(172.50,282.10) | 3.07(2.37,3.86) | 415.66(297.67,561.92) | 3.44(2.48,4.65) | 0.35(0.04,0.65) |
| Kingdom of Sweden | 43.19(32.95,55.70) | 2.24(1.70,2.90) | 89.22(64.07,120.83) | 3.28(2.35,4.46) | 1.01(-0.15,2.18) |
| Kingdom of Thailand | 64.36(39.27,101.69) | 1.59(0.97,2.52) | 431.34(264.36,696.38) | 3.04(1.87,4.91) | 1.72(1.57,1.87) |
| Kingdom of the Netherlands | 122.68(94.43,154.91) | 4.75(3.66,5.99) | 216.82(158.93,288.16) | 4.68(3.43,6.22) | -0.42(-0.81,-0.04) |
| Kingdom of Tonga | 0.03(0.01,0.07) | 0.42(0.15,0.97) | 0.05(0.02,0.11) | 0.55(0.25,1.09) | 0.67(0.47,0.88) |
| Kyrgyz Republic | 1.66(1.17,2.29) | 0.44(0.31,0.61) | 15.24(10.75,20.92) | 2.64(1.86,3.63) | 8.50(7.30,9.71) |
| Lao People's Democratic Republic | 1.04(0.42,2.40) | 0.41(0.16,0.95) | 5.72(2.82,10.45) | 1.07(0.53,1.96) | 3.39(3.29,3.49) |
| Lebanese Republic | 2.44(1.37,4.26) | 0.93(0.52,1.61) | 12.50(7.22,20.14) | 1.69(0.98,2.73) | 2.75(2.47,3.02) |
| Malaysia | 25.10(13.88,43.78) | 2.38(1.31,4.16) | 179.35(110.84,272.58) | 5.02(3.10,7.63) | 2.22(1.98,2.47) |
| Mongolia | 2.18(1.13,3.83) | 1.75(0.90,3.08) | 10.23(6.32,15.92) | 3.93(2.44,6.07) | 2.66(2.54,2.79) |
| Montenegro | 1.36(0.86,1.94) | 1.76(1.11,2.53) | 4.13(2.91,5.57) | 3.12(2.19,4.21) | 2.19(2.01,2.36) |
| New Zealand | 21.54(16.77,27.37) | 4.14(3.22,5.26) | 45.31(34.71,58.79) | 4.11(3.15,5.32) | 0.05(-0.56,0.67) |
| North Macedonia | 3.21(2.34,4.27) | 1.39(1.01,1.85) | 12.33(8.58,16.77) | 2.78(1.94,3.79) | 2.31(1.93,2.70) |
| Northern Mariana Islands | 0.00(0.00,0.00) | 0.17(0.07,0.33) | 0.01(0.01,0.02) | 0.24(0.13,0.39) | 0.99(0.07,1.91) |
| Palestine | 1.17(0.53,2.43) | 1.13(0.50,2.33) | 7.11(4.25,10.90) | 2.53(1.52,3.89) | 2.97(2.79,3.15) |
| People's Democratic Republic of Algeria | 8.41(4.60,15.21) | 0.60(0.33,1.08) | 44.91(25.04,79.98) | 1.09(0.61,1.93) | 1.82(1.74,1.90) |
| People's Republic of Bangladesh | 15.34(5.48,34.08) | 0.28(0.10,0.63) | 110.94(49.88,205.68) | 0.65(0.30,1.21) | 2.47(2.24,2.70) |
| People's Republic of China | 1051.98(721.44,1562.48) | 1.05(0.71,1.57) | 10464.64(7140.82,13312.97) | 3.89(2.66,4.94) | 4.97(4.63,5.30) |
| Plurinational State of Bolivia | 6.31(3.76,10.15) | 1.66(0.99,2.68) | 41.02(23.61,68.54) | 3.67(2.11,6.11) | 2.56(2.45,2.68) |
| Portuguese Republic | 52.17(39.38,67.22) | 2.80(2.11,3.62) | 88.22(62.10,120.66) | 2.82(1.98,3.90) | -0.14(-0.44,0.17) |
| Principality of Andorra | 0.20(0.10,0.36) | 2.73(1.41,4.80) | 0.54(0.29,0.96) | 2.84(1.50,5.00) | 0.63(0.39,0.87) |
| Principality of Monaco | 0.00(0.00,0.01) | 0.05(0.03,0.09) | 0.01(0.00,0.01) | 0.06(0.03,0.10) | 0.53(0.47,0.59) |
| Puerto Rico | 7.30(5.12,10.01) | 1.60(1.12,2.20) | 23.87(16.34,33.58) | 2.63(1.80,3.72) | 1.69(1.25,2.14) |
| Republic of Albania | 0.97(0.62,1.50) | 0.40(0.26,0.62) | 4.36(2.51,7.15) | 0.73(0.42,1.20) | 2.28(2.05,2.51) |
| Republic of Angola | 1.82(0.61,4.21) | 0.42(0.14,1.00) | 12.52(5.14,26.20) | 0.99(0.40,2.08) | 2.60(2.39,2.81) |
| Republic of Armenia | 6.84(3.62,11.46) | 1.97(1.03,3.29) | 48.15(31.47,71.43) | 8.04(5.27,11.90) | 5.71(5.06,6.37) |
| Republic of Austria | 48.67(37.95,61.82) | 3.13(2.45,3.98) | 64.56(47.58,84.43) | 2.85(2.10,3.74) | -0.18(-0.54,0.18) |
| Republic of Azerbaijan | 11.29(6.05,20.45) | 1.96(1.03,3.60) | 32.43(18.65,54.17) | 2.65(1.51,4.41) | 1.69(1.31,2.07) |
| Republic of Belarus | 40.01(27.29,55.83) | 2.37(1.63,3.30) | 195.09(128.15,284.16) | 8.83(5.80,12.83) | 3.83(3.35,4.31) |
| Republic of Benin | 0.30(0.16,0.54) | 0.13(0.07,0.23) | 1.02(0.55,1.79) | 0.19(0.10,0.33) | 1.08(0.93,1.23) |
| Republic of Botswana | 0.81(0.37,1.59) | 1.22(0.56,2.40) | 4.68(2.61,8.01) | 2.87(1.62,4.86) | 2.79(2.50,3.08) |
| Republic of Bulgaria | 20.29(13.07,28.86) | 1.21(0.79,1.72) | 52.58(34.65,75.68) | 2.77(1.81,4.01) | 1.62(0.98,2.26) |
| Republic of Burundi | 0.90(0.41,1.82) | 0.32(0.15,0.66) | 2.34(0.90,5.21) | 0.43(0.17,0.95) | 0.67(0.47,0.86) |
| Republic of Cabo Verde | 0.01(0.00,0.02) | 0.03(0.01,0.05) | 0.05(0.02,0.09) | 0.10(0.04,0.18) | 4.49(4.26,4.73) |
| Republic of Cameroon | 1.03(0.60,1.67) | 0.21(0.12,0.34) | 4.07(2.22,6.70) | 0.31(0.17,0.52) | 1.08(0.94,1.22) |
| Republic of Chad | 0.23(0.09,0.48) | 0.07(0.03,0.14) | 0.71(0.31,1.37) | 0.12(0.05,0.23) | 1.65(1.55,1.76) |
| Republic of Chile | 11.18(8.40,14.49) | 0.91(0.68,1.18) | 99.49(72.13,132.10) | 3.02(2.19,4.00) | 4.14(3.00,5.29) |
| Republic of Colombia | 32.32(24.16,41.70) | 1.59(1.19,2.04) | 210.83(145.62,290.48) | 3.07(2.12,4.22) | 1.32(0.62,2.03) |
| Republic of Costa Rica | 3.58(2.60,4.72) | 1.72(1.25,2.27) | 26.89(19.09,36.24) | 3.88(2.75,5.22) | 1.76(1.23,2.30) |
| Republic of Croatia | 34.66(25.53,45.60) | 4.47(3.31,5.84) | 108.77(75.59,151.64) | 9.14(6.36,12.73) | 1.98(1.60,2.36) |
| Republic of Cuba | 26.20(19.30,34.89) | 2.05(1.51,2.74) | 73.66(53.10,99.42) | 3.10(2.23,4.18) | 2.30(1.77,2.82) |
| Republic of Cyprus | 3.92(2.00,7.01) | 3.95(2.02,7.04) | 12.63(7.81,19.34) | 4.63(2.85,7.08) | 0.37(0.10,0.65) |
| The Republic of Côte d'Ivoire | 0.47(0.24,0.83) | 0.11(0.06,0.20) | 2.16(1.17,3.84) | 0.18(0.10,0.33) | 1.22(1.06,1.39) |
| Republic of Djibouti | 0.08(0.03,0.14) | 0.54(0.24,1.00) | 0.85(0.44,1.50) | 1.24(0.66,2.16) | 2.66(2.62,2.69) |
| Republic of Ecuador | 9.41(6.83,12.56) | 1.53(1.11,2.05) | 91.48(62.54,132.05) | 4.55(3.11,6.57) | 4.10(3.53,4.67) |
| Republic of El Salvador | 2.46(1.55,3.53) | 0.70(0.44,1.00) | 10.17(6.92,14.60) | 1.35(0.92,1.94) | 2.00(1.80,2.19) |
| Republic of Equatorial Guinea | 0.11(0.04,0.25) | 0.47(0.19,1.11) | 1.24(0.64,2.22) | 2.35(1.22,4.16) | 5.70(5.56,5.83) |
| Republic of Estonia | 7.80(5.32,11.12) | 2.93(2.00,4.16) | 36.61(24.32,52.18) | 10.74(7.11,15.37) | 2.97(2.41,3.54) |
| Republic of Fiji | 0.77(0.34,1.56) | 2.14(0.93,4.28) | 1.68(0.71,4.46) | 1.87(0.82,4.83) | -1.49(-2.09,-0.89) |
| Republic of Finland | 20.98(15.55,27.50) | 2.23(1.66,2.92) | 85.48(60.79,117.23) | 5.23(3.74,7.16) | 2.68(2.26,3.10) |
| Republic of Ghana | 1.27(0.24,2.44) | 0.20(0.03,0.38) | 2.59(1.36,4.33) | 0.15(0.08,0.26) | -2.59(-3.74,-1.42) |
| Republic of Guatemala | 1.95(1.51,2.50) | 0.50(0.39,0.65) | 7.86(6.16,9.68) | 0.59(0.46,0.72) | 0.13(-0.13,0.38) |
| Republic of Guinea | 0.57(0.31,1.01) | 0.14(0.07,0.25) | 1.70(0.84,3.16) | 0.26(0.13,0.50) | 2.02(1.95,2.09) |
| Republic of Guinea-Bissau | 0.07(0.04,0.11) | 0.15(0.09,0.25) | 0.16(0.09,0.25) | 0.22(0.13,0.36) | 0.99(0.88,1.11) |
| Republic of Guyana | 0.04(0.03,0.05) | 0.09(0.06,0.12) | 1.25(0.82,1.86) | 1.51(0.99,2.24) | 7.64(5.39,9.93) |
| Republic of Haiti | 1.92(0.83,4.00) | 0.48(0.21,1.03) | 7.34(3.45,14.01) | 0.87(0.41,1.68) | 2.10(1.91,2.29) |
| Republic of Honduras | 1.95(0.84,4.06) | 0.82(0.35,1.71) | 22.29(13.07,36.47) | 2.93(1.71,4.79) | 4.44(4.15,4.73) |
| Republic of Iceland | 1.14(0.86,1.45) | 3.11(2.34,3.95) | 3.28(2.33,4.42) | 4.34(3.08,5.85) | 1.26(1.02,1.50) |
| Republic of India | 245.85(148.43,368.13) | 0.46(0.28,0.69) | 1640.06(1343.61,2014.31) | 1.12(0.92,1.37) | 2.45(2.12,2.78) |
| Republic of Indonesia | 85.21(54.50,120.32) | 0.75(0.48,1.06) | 636.18(446.01,844.92) | 2.22(1.55,2.94) | 3.34(3.20,3.48) |
| Republic of Iraq | 4.30(1.97,8.37) | 0.47(0.22,0.92) | 36.11(20.08,59.19) | 1.35(0.75,2.21) | 3.40(3.23,3.57) |
| Republic of Italy | 310.29(276.60,340.72) | 2.64(2.35,2.90) | 635.40(530.44,731.31) | 3.55(2.99,4.06) | 1.21(0.88,1.53) |
| Republic of Kazakhstan | 20.20(11.66,29.75) | 1.27(0.73,1.89) | 55.17(33.59,84.44) | 2.45(1.50,3.74) | 1.76(1.54,1.98) |
| Republic of Kenya | 2.11(0.83,4.31) | 0.22(0.09,0.45) | 19.06(12.12,28.03) | 0.72(0.46,1.06) | 3.86(3.78,3.94) |
| Republic of Kiribati | 0.00(0.00,0.01) | 0.09(0.04,0.20) | 0.01(0.00,0.02) | 0.13(0.06,0.27) | 1.20(1.13,1.28) |
| Republic of Korea | 77.38(47.00,118.32) | 2.27(1.37,3.50) | 236.90(148.95,359.21) | 1.90(1.19,2.88) | -1.14(-1.40,-0.88) |
| Republic of Latvia | 9.71(6.43,14.27) | 2.09(1.38,3.06) | 25.21(16.23,37.79) | 5.02(3.23,7.54) | 3.36(3.08,3.65) |
| Republic of Liberia | 0.18(0.09,0.33) | 0.13(0.06,0.24) | 0.31(0.15,0.58) | 0.15(0.07,0.29) | 0.67(0.40,0.96) |
| Republic of Lithuania | 10.95(7.31,15.71) | 1.89(1.26,2.71) | 59.17(39.34,84.41) | 8.15(5.40,11.65) | 5.00(4.73,5.27) |
| Republic of Madagascar | 2.30(1.11,4.09) | 0.38(0.18,0.68) | 7.44(3.90,13.22) | 0.61(0.32,1.08) | 1.55(1.22,1.87) |
| Republic of Malawi | 3.67(1.89,6.33) | 0.80(0.41,1.37) | 14.44(8.29,23.18) | 1.70(0.98,2.72) | 2.51(2.41,2.62) |
| Republic of Maldives | 0.26(0.11,0.54) | 2.51(1.05,5.38) | 2.07(1.22,3.27) | 5.97(3.51,9.45) | 2.43(1.95,2.91) |
| Republic of Mali | 0.35(0.20,0.61) | 0.07(0.04,0.14) | 1.35(0.73,2.41) | 0.14(0.07,0.25) | 1.90(1.78,2.02) |
| Republic of Malta | 3.13(2.39,4.01) | 5.70(4.36,7.30) | 8.01(5.85,10.59) | 6.17(4.50,8.15) | 0.08(-0.28,0.45) |
| Republic of Mauritius | 1.27(1.08,1.49) | 1.38(1.17,1.62) | 9.71(8.04,11.39) | 3.91(3.24,4.60) | 3.22(2.24,4.21) |
| Republic of Moldova | 7.66(5.55,10.66) | 1.31(0.95,1.82) | 31.46(25.53,38.65) | 3.85(3.13,4.73) | 4.15(3.61,4.70) |
| Republic of Mozambique | 1.81(0.63,4.23) | 0.26(0.09,0.62) | 7.88(3.57,16.53) | 0.64(0.29,1.34) | 3.24(3.06,3.43) |
| Republic of Namibia | 0.71(0.35,1.30) | 0.87(0.43,1.63) | 2.71(1.46,5.25) | 1.70(0.92,3.35) | 1.99(1.77,2.20) |
| Republic of Nauru | 0.00(0.00,0.01) | 0.81(0.31,1.84) | 0.01(0.00,0.01) | 0.91(0.47,1.61) | 0.29(0.04,0.55) |
| Republic of Nicaragua | 2.89(1.72,4.48) | 1.65(0.98,2.57) | 17.48(12.52,24.11) | 3.00(2.15,4.13) | 1.94(1.61,2.27) |
| Republic of Niue | 0.00(0.00,0.00) | 0.72(0.31,1.40) | 0.00(0.00,0.00) | 0.90(0.45,1.58) | 0.58(0.40,0.76) |
| Republic of Palau | 0.00(0.00,0.01) | 0.30(0.13,0.60) | 0.01(0.00,0.02) | 0.29(0.14,0.53) | -0.10(-0.18,-0.03) |
| Republic of Panama | 3.90(3.30,4.57) | 2.21(1.87,2.60) | 15.13(11.18,19.36) | 2.79(2.06,3.58) | 0.53(0.33,0.74) |
| Republic of Paraguay | 2.07(1.14,3.51) | 0.78(0.43,1.33) | 12.43(7.19,19.60) | 1.75(1.01,2.76) | 2.68(2.56,2.81) |
| Republic of Peru | 24.02(15.18,37.16) | 1.73(1.09,2.68) | 126.71(75.68,202.66) | 3.14(1.87,5.01) | 1.98(1.85,2.11) |
| Republic of Poland | 120.62(110.67,131.26) | 2.10(1.92,2.29) | 383.86(339.79,426.78) | 3.91(3.47,4.35) | 1.41(0.36,2.47) |
| Republic of Rwanda | 1.73(0.97,2.93) | 0.51(0.29,0.86) | 6.46(3.36,11.53) | 0.88(0.46,1.55) | 1.51(1.19,1.85) |
| Republic of San Marino | 0.03(0.02,0.05) | 0.75(0.45,1.18) | 0.05(0.02,0.08) | 0.52(0.26,0.90) | -0.31(-0.73,0.11) |
| Republic of Senegal | 0.53(0.26,0.94) | 0.14(0.07,0.25) | 2.21(1.24,3.73) | 0.26(0.15,0.43) | 1.80(1.59,2.00) |
| Republic of Serbia | 58.24(32.60,95.76) | 4.07(2.29,6.72) | 129.99(81.55,199.72) | 5.81(3.65,8.93) | 1.00(0.88,1.12) |
| Republic of Seychelles | 0.02(0.00,0.04) | 0.27(0.00,0.53) | 0.05(0.00,0.10) | 0.39(0.01,0.72) | 1.33(1.14,1.52) |
| Republic of Sierra Leone | 0.27(0.13,0.49) | 0.11(0.05,0.20) | 0.67(0.36,1.17) | 0.17(0.09,0.29) | 1.53(1.26,1.79) |
| Republic of Singapore | 7.63(5.86,9.91) | 2.90(2.23,3.77) | 28.19(19.84,38.69) | 2.52(1.76,3.46) | -0.69(-1.16,-0.22) |
| Republic of Slovenia | 7.12(5.34,9.30) | 2.28(1.71,2.97) | 16.80(11.71,23.72) | 2.92(2.04,4.12) | 0.62(-0.08,1.32) |
| Republic of South Africa | 47.53(24.58,67.43) | 1.97(1.01,2.80) | 198.61(131.39,241.25) | 3.57(2.33,4.35) | 1.83(1.57,2.10) |
| Republic of South Sudan | 0.77(0.22,1.90) | 0.24(0.07,0.60) | 1.80(0.67,4.09) | 0.44(0.16,0.99) | 1.91(1.78,2.05) |
| Republic of Sudan | 0.68(0.15,3.71) | 0.06(0.01,0.34) | 5.91(2.64,14.25) | 0.28(0.12,0.66) | 5.67(5.31,6.02) |
| Republic of Suriname | 0.15(0.08,0.28) | 0.48(0.27,0.91) | 0.66(0.36,1.09) | 0.83(0.46,1.39) | 2.44(2.04,2.85) |
| Republic of Tajikistan | 0.19(0.08,0.37) | 0.06(0.02,0.11) | 0.44(0.25,0.69) | 0.07(0.04,0.10) | 0.20(-0.07,0.47) |
| Republic of the Congo | 1.39(0.78,2.34) | 1.09(0.60,1.85) | 5.01(2.69,8.17) | 1.75(0.94,2.87) | 1.41(1.23,1.59) |
| Republic of the Gambia | 0.08(0.05,0.15) | 0.21(0.12,0.39) | 0.48(0.22,0.91) | 0.44(0.20,0.86) | 2.08(1.88,2.29) |
| Republic of the Marshall Islands | 0.01(0.00,0.02) | 0.42(0.16,1.01) | 0.02(0.01,0.04) | 0.60(0.28,1.11) | 0.91(0.81,1.00) |
| Republic of the Niger | 0.19(0.06,0.41) | 0.06(0.02,0.14) | 0.65(0.21,1.53) | 0.07(0.02,0.18) | 0.49(0.36,0.62) |
| Republic of the Philippines | 47.46(26.45,63.71) | 1.46(0.81,1.97) | 291.85(203.58,364.67) | 2.95(2.04,3.68) | 2.34(2.29,2.40) |
| Republic of the Union of Myanmar | 14.37(7.44,27.92) | 0.50(0.26,0.99) | 74.81(42.06,124.61) | 1.25(0.71,2.08) | 3.21(3.12,3.29) |
| Republic of Trinidad and Tobago | 3.46(2.77,4.26) | 3.38(2.70,4.16) | 15.44(11.17,20.36) | 5.96(4.32,7.85) | 2.09(1.96,2.21) |
| Republic of Tunisia | 7.28(3.59,13.29) | 1.19(0.59,2.16) | 44.80(23.26,77.05) | 2.66(1.39,4.57) | 2.51(2.35,2.68) |
| Republic of Turkey | 71.91(35.22,131.02) | 1.76(0.86,3.21) | 557.06(341.38,850.61) | 4.76(2.92,7.26) | 3.43(3.07,3.79) |
| Republic of Uganda | 1.84(1.04,3.03) | 0.24(0.14,0.40) | 10.28(5.60,17.35) | 0.64(0.35,1.08) | 2.96(2.81,3.11) |
| Republic of Uzbekistan | 36.83(13.79,64.72) | 2.64(0.98,4.65) | 158.62(100.04,230.57) | 4.95(3.14,7.15) | 2.31(2.11,2.50) |
| Republic of Vanuatu | 0.02(0.00,0.05) | 0.24(0.06,0.72) | 0.07(0.03,0.16) | 0.33(0.13,0.79) | 0.80(0.69,0.91) |
| Republic of Yemen | 0.24(0.03,1.40) | 0.04(0.01,0.25) | 3.47(1.39,9.45) | 0.22(0.09,0.59) | 6.31(5.92,6.69) |
| Republic of Zambia | 1.52(0.78,2.61) | 0.48(0.25,0.82) | 12.75(5.74,24.83) | 1.71(0.78,3.27) | 4.51(4.01,5.02) |
| Republic of Zimbabwe | 3.63(2.08,5.96) | 0.76(0.43,1.25) | 9.21(5.06,15.68) | 1.16(0.64,1.98) | 0.60(0.17,1.02) |
| Romania | 78.84(50.22,117.94) | 2.16(1.38,3.21) | 169.94(115.72,239.91) | 3.47(2.36,4.89) | 1.40(1.21,1.58) |
| Russian Federation | 913.41(610.08,1256.85) | 3.74(2.52,5.13) | 1394.93(1213.29,1577.49) | 4.18(3.65,4.73) | -1.55(-2.47,-0.62) |
| Saint Kitts and Nevis | 0.05(0.03,0.07) | 0.94(0.61,1.41) | 0.18(0.11,0.30) | 1.91(1.13,3.15) | 3.31(2.71,3.92) |
| Saint Lucia | 0.12(0.08,0.18) | 1.11(0.69,1.65) | 0.60(0.35,0.92) | 1.99(1.15,3.04) | 1.60(1.47,1.73) |
| Saint Vincent and the Grenadines | 0.00(0.00,0.01) | 0.04(0.04,0.05) | 0.36(0.28,0.46) | 1.95(1.52,2.45) | 9.18(5.59,12.90) |
| Slovak Republic | 21.06(13.45,30.96) | 2.67(1.70,3.93) | 50.95(30.89,78.21) | 3.96(2.40,6.08) | 1.22(0.90,1.54) |
| Socialist Republic of Viet Nam | 44.20(25.80,75.16) | 0.88(0.51,1.49) | 251.43(138.56,455.35) | 2.09(1.16,3.76) | 2.68(2.52,2.85) |
| Solomon Islands | 0.03(0.01,0.11) | 0.21(0.04,0.68) | 0.11(0.04,0.28) | 0.30(0.11,0.73) | 0.74(0.54,0.94) |
| State of Eritrea | 0.42(0.21,0.84) | 0.37(0.18,0.74) | 2.68(1.43,4.54) | 0.92(0.50,1.56) | 2.92(2.64,3.19) |
| State of Israel | 18.82(13.54,25.57) | 2.95(2.12,4.01) | 49.42(35.23,67.13) | 3.12(2.23,4.22) | -0.07(-0.46,0.32) |
| State of Kuwait | 0.66(0.48,0.90) | 1.12(0.81,1.53) | 5.05(3.33,7.32) | 1.80(1.19,2.60) | 4.18(2.46,5.93) |
| State of Libya | 2.80(1.07,6.07) | 1.29(0.49,2.78) | 16.52(8.53,28.91) | 2.97(1.54,5.20) | 2.84(2.32,3.36) |
| State of Qatar | 0.04(0.02,0.06) | 0.44(0.24,0.74) | 0.57(0.30,0.97) | 0.81(0.42,1.38) | 3.26(2.58,3.95) |
| Sultanate of Oman | 0.55(0.17,1.23) | 0.75(0.23,1.68) | 3.39(1.83,5.55) | 1.71(0.93,2.82) | 2.31(1.89,2.74) |
| Swiss Confederation | 44.33(33.53,57.12) | 3.35(2.53,4.32) | 124.20(87.60,169.44) | 5.49(3.88,7.50) | 0.97(0.51,1.43) |
| Syrian Arab Republic | 1.49(0.51,3.39) | 0.24(0.08,0.55) | 9.14(4.42,16.38) | 0.56(0.28,1.00) | 2.69(2.37,3.01) |
| Taiwan (Province of China) | 42.51(32.81,53.96) | 2.09(1.61,2.66) | 221.05(158.31,302.81) | 3.92(2.80,5.38) | 1.40(1.00,1.80) |
| Togolese Republic | 0.20(0.12,0.34) | 0.15(0.09,0.26) | 0.83(0.44,1.40) | 0.22(0.12,0.36) | 0.84(0.68,1.01) |
| Tokelau | 0.00(0.00,0.00) | 0.62(0.25,1.29) | 0.00(0.00,0.00) | 0.77(0.36,1.44) | 0.52(0.30,0.75) |
| Turkmenistan | 4.57(2.67,7.14) | 1.97(1.14,3.09) | 19.00(10.58,31.53) | 3.91(2.19,6.45) | 2.45(2.20,2.69) |
| Tuvalu | 0.00(0.00,0.01) | 0.38(0.14,0.97) | 0.01(0.00,0.01) | 0.53(0.24,1.06) | 0.61(0.31,0.90) |
| Ukraine | 333.70(193.96,510.29) | 3.37(1.95,5.17) | 487.84(299.34,738.38) | 4.59(2.82,6.94) | 1.01(0.81,1.21) |
| Union of the Comoros | 0.10(0.04,0.19) | 0.41(0.17,0.79) | 0.62(0.34,1.05) | 1.09(0.59,1.85) | 3.05(2.90,3.20) |
| United Arab Emirates | 0.55(0.24,1.13) | 1.54(0.66,3.16) | 7.95(4.50,13.02) | 3.07(1.77,4.98) | 3.46(2.75,4.17) |
| United Kingdom of Great Britain and Northern Ireland | 502.22(471.87,527.67) | 4.20(3.95,4.42) | 578.69(527.69,617.04) | 3.46(3.18,3.69) | -0.62(-0.94,-0.30) |
| United Mexican States | 57.75(53.94,61.31) | 1.18(1.10,1.26) | 470.16(404.14,539.31) | 3.03(2.61,3.48) | 2.50(1.56,3.45) |
| United Republic of Tanzania | 5.02(2.21,10.04) | 0.38(0.17,0.76) | 24.31(12.75,42.76) | 0.85(0.45,1.49) | 2.61(2.45,2.78) |
| United States of America | 1230.18(1103.68,1342.60) | 2.95(2.64,3.22) | 2426.96(2148.32,2662.63) | 3.11(2.76,3.42) | -0.01(-0.37,0.36) |
| United States Virgin Islands | 0.09(0.05,0.15) | 0.85(0.47,1.45) | 0.27(0.14,0.50) | 1.11(0.55,2.04) | 1.03(0.48,1.59) |

EAPC: Estimated Annual Percentage Change; DALYs: Disability-Adjusted Life Years
